# A peripheral subpopulation of retinal pigment epithelium resists oxidative damage through SERPINE3-mediated caspase-1 inhibition

**DOI:** 10.1172/JCI191272

**Published:** 2026-07-07

**Authors:** Huirong Li, Takerra Johnson-Stephenson, Vincent P. Kunze, Wei Yan, David M. McGaughey, Temesgen D. Fufa, Koray Dogan Kaya, Ashley M. Rasys, Davide Ortolan, Dominik Reichert, Congxiao Zhang, Ruchi Sharma, Lijin Dong, Bin Guan, Brian P. Brooks, Tiansen Li, Wei Li, Wencan Wu, Kapil Bharti, Robert B. Hufnagel

**Affiliations:** 1 Ophthalmic Genetics and Visual Function Branch, National Eye Institute, NIH, Bethesda, Maryland, USA.; 2 Zhejiang Key Laboratory of Key Technologies for Visual Pathway Reconstruction, Eye Hospital, Wenzhou Medical University, Wenzhou, China.; 3 Retinal Neurophysiology Section, National Eye Institute, NIH, Bethesda, Maryland, USA.; 4 Sanya Institute, China Agricultural University, Sanya, China.; 5 State Key Laboratory of Animal Biotech Breeding, National Engineering Laboratory for Animal Breeding, College of Animal Science and Technology, China Agricultural University, Beijing, China.; 6 Bioinformatics Group, National Eye Institute, NIH, Bethesda, Maryland, USA.; 7 Division of Genome Sciences, National Human Genome Research Institute, NIH, Bethesda, Maryland, USA.; 8 Genetic Engineering Core Facility and; 9 Retinal Cell Biology and Degeneration, National Eye Institute, NIH, Bethesda, Maryland, USA.; 10 SANS Institute for Neuroscience and Vision Research, State Key Laboratory of Eye Health, Shanghai Jiao Tong University School of Medicine, Shanghai, China.; 11 Center for Integrated Healthcare Research, Kaiser Permanente Hawaii, Honolulu, Hawaii, USA.

**Keywords:** Cell biology, Ophthalmology, Retinopathy

## Abstract

Heterogeneous degeneration of the retinal pigment epithelium (RPE) leads to irreversible blindness in diseases associated with macular atrophy. However, the underlying mechanisms of regional RPE degeneration remain poorly understood. To address this gap, this study identified a peripheral RPE subpopulation through spatial, transcriptomic, and functional analyses, thereby contributing to the understanding of the heterogeneity of degenerative RPE cells. Specifically, omics analyses in human and macaque RPE revealed a peripheral RPE cell population with high SERPINE3 expression, while SERPINE3-GFP–knockin mice showed comparable expression patterns. SMART RNA-seq2 analysis further distinguished transcriptomic profiles between GFP^+^ and GFP^–^ RPE cells. Under oxidative stress, SERPINE3 expression increased, and GFP^+^ cells exhibited improved survival and reentry into the cell cycle. Notably, genetic studies indicated that SERPINE3 is essential for the oxidative stress resistance of GFP^+^ cells. Moreover, loss of SERPINE3 resulted in regional RPE degeneration and increased microglial accumulation in aged mice. Mechanistically, proteinase screening and co-IP indicated that SERPINE3 targets caspase-1. Importantly, delivery of SERPINE3 via AAV-Serpine3 partially reduced RPE degeneration in an oxidative damage model. These findings advance the understanding of RPE heterogeneous degeneration and highlight SERPINE3 as a protective factor with therapeutic potential for macular atrophy.

## Abstract 2

A peripheral RPE subpopulation contributes to the degenerative retinal pigment eipthelium heterogeneity

## Graphical abstract

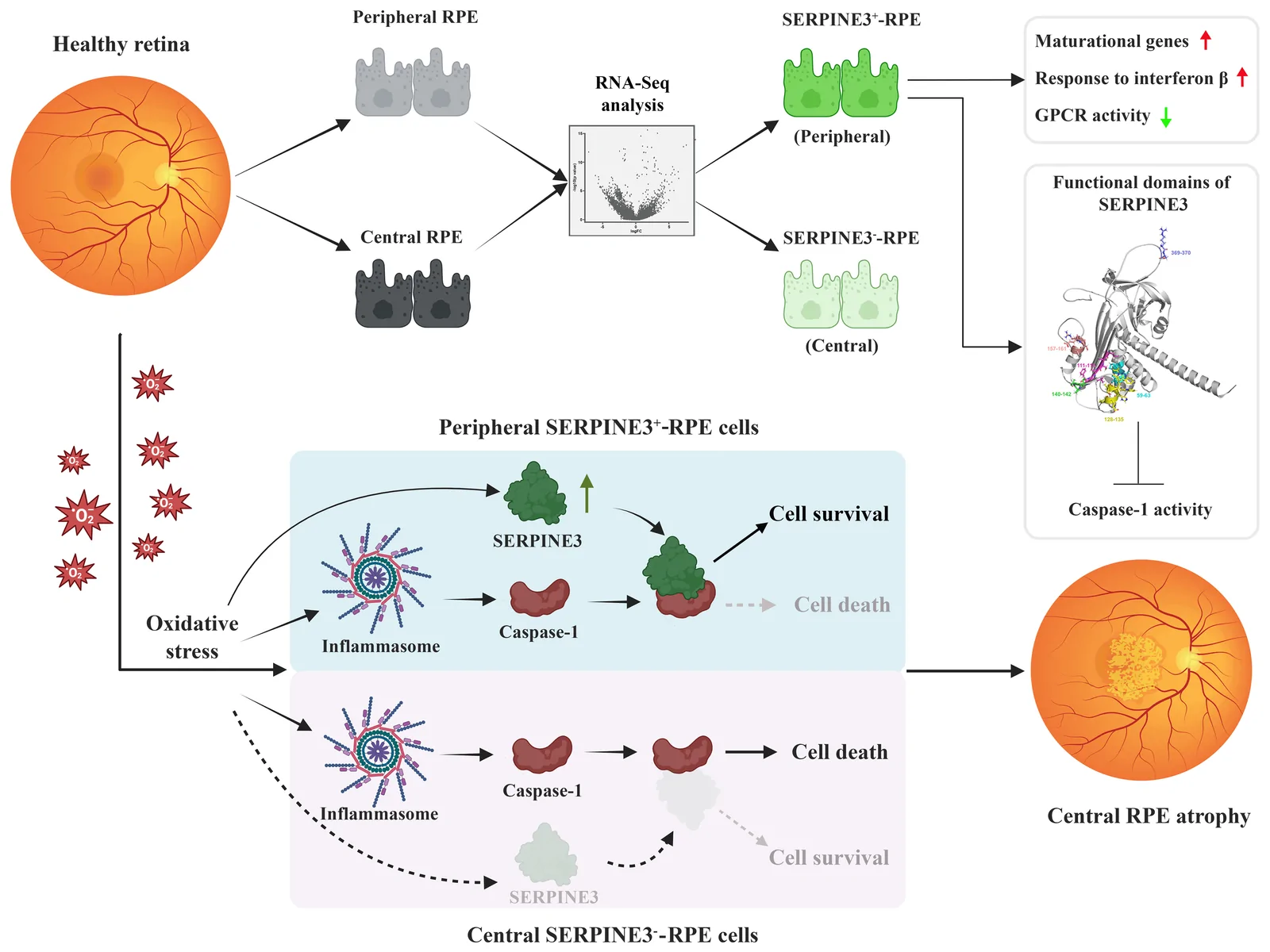

## Introduction

Macular degeneration is the main cause of irreversible blindness that features central vision loss. It often presents in macular dystrophies and age-related macular degeneration (AMD), a leading cause of blindness among the aged people in the developed world, and the treatments are limited (1, 2). The most common pathogenic phenotype among these diseases is the marked loss of retinal pigment epithelium (RPE) in the macula (3). RPE is the outermost layer of the retina, situated between photoreceptors and the choriocapillaris. It consists of a monolayer of pigmented cells with a typical hexagonal structure and polarization and supports the neural retina by transporting nutrients to the overlaying photoreceptor cells, phagocytizing photoreceptor outer segments, and replenishing the 11-cis-retinal for visual cycling (4–6). Based on its important function, macular RPE degeneration undoubtedly leads to central vision loss in the macular atrophy–associated diseases, but the peripheral RPE often remains relatively healthy and preserves the peripheral vision (3, 7–9). This degenerative RPE heterogeneity provides an avenue to understand how peripheral RPE cells survive under the pathological condition, which could be applied as a treatment of macular RPE degeneration. Presently, it is still unclear why macular RPE cells are more susceptible in AMD or if peripheral RPE cells are more resistant to age-related degeneration.

Given the difference in light exposure between macular and peripheral RPE, photooxidative stress primarily affects the macular region, making it more susceptible to damage in AMD (10–12). In addition, to match the higher demand of high-density photoreceptors in the macula, associated RPE cells exhibit higher lipid metabolism compared with the peripheral RPE cells, likely leading to excessive lipofuscin deposits in the macular RPE (10, 13, 14). It is thus not surprising that macular RPE tends to have higher susceptibility toward degeneration (14, 15). Supplements and treatments that improve RPE cell health or reduce degeneration have been shown to slow the progression of AMD (16–18). However, these supplements and treatments are limited in preventing macular RPE degeneration and do not improve visual function in later stages of AMD. This inefficiency probably can be attributed to our incomplete understanding of RPE ROS and metabolic processes. Although excessive generation of ROS is considered to be harmful, controlled ROS generation can have beneficial effects and underscores the cell’s ability to mount relative resistance to ROS levels (19–21). Intriguingly, the relative resistance of peripheral RPE to oxidative stress is observed not only in AMD patients (15) but also in AMD disease models generated by chemical oxidants (22–24). This suggests that the peripheral RPE cells may have endogenous antioxidant pathways that are either missing or insufficient in macular RPE cells. Understanding heterogeneity between macular and peripheral RPE antioxidant responses will help uncover the endogenous antioxidant pathways.

Although RPE was once thought to be a homogeneous tissue, it has been shown to have regional differences in cell morphology, pigmentation, and multiple nuclei between the central and peripheral areas (25–28). Recently, using artificial intelligence, we categorized human RPE cells into 5 subpopulations along the central to the peripheral axis according to cell size at single-cell resolution (7). The specific RPE subpopulations are differentially susceptible to aging and retinal degenerative diseases, such as choroideremia, late-onset retinal degeneration, and AMD (7), suggesting that each RPE subpopulation has different molecular features that change its susceptibility to different diseases. scRNA-seq analysis further supported transcriptomic differences between macular and peripheral RPE cells (29–33). These transcriptomic changes are associated with different pathways, including the lipid metabolism, visual cycle, and oxidative stress pathways. However, it is still challenging to distinguish the peripheral RPE subpopulation due to lack of specific biomarkers, and how the peripheral RPE resists oxidative stress remains ambiguous.

To characterize the features of peripheral RPE cells, we used bulk and scRNA-seq data to further define RPE subpopulations at the transcriptomic level. We discovered that a minor subpopulation of RPE cells express *SERPINE3*, a serine protease reported to be expressed in Müller glial cells in zebrafish (34). However, its expression and function in RPE or in mammalian eyes has not been explored. This study uncovered a conserved peripheral location of *SERPINE3*-expressing RPE cells in humans, macaques, and mice. We further showed that this subpopulation of peripheral RPE cells has specific transcriptomic features and is resistant to oxidative stress in a SERPINE3-dependent manner. Furthermore, deletion of Serpine3 led to RPE regional degeneration and inflammation in aged mice. Using a proteinase screen and co-IP, we found that SERPINE3 can inhibit caspase-1 activity and bind caspase-1. Our findings indicate that a subpopulation of peripheral RPE cells exhibits increased resistance to oxidative stress mediated by SERPINE3. This highlights functionally significant differences between macular and peripheral RPE that may have important pathophysiological implications and suggests a potential strategy for protecting macular RPE from oxidative injury.

## Results

### A minor SERPINE3^+^-RPE subpopulation resides at the periphery.

**Figure 1:**
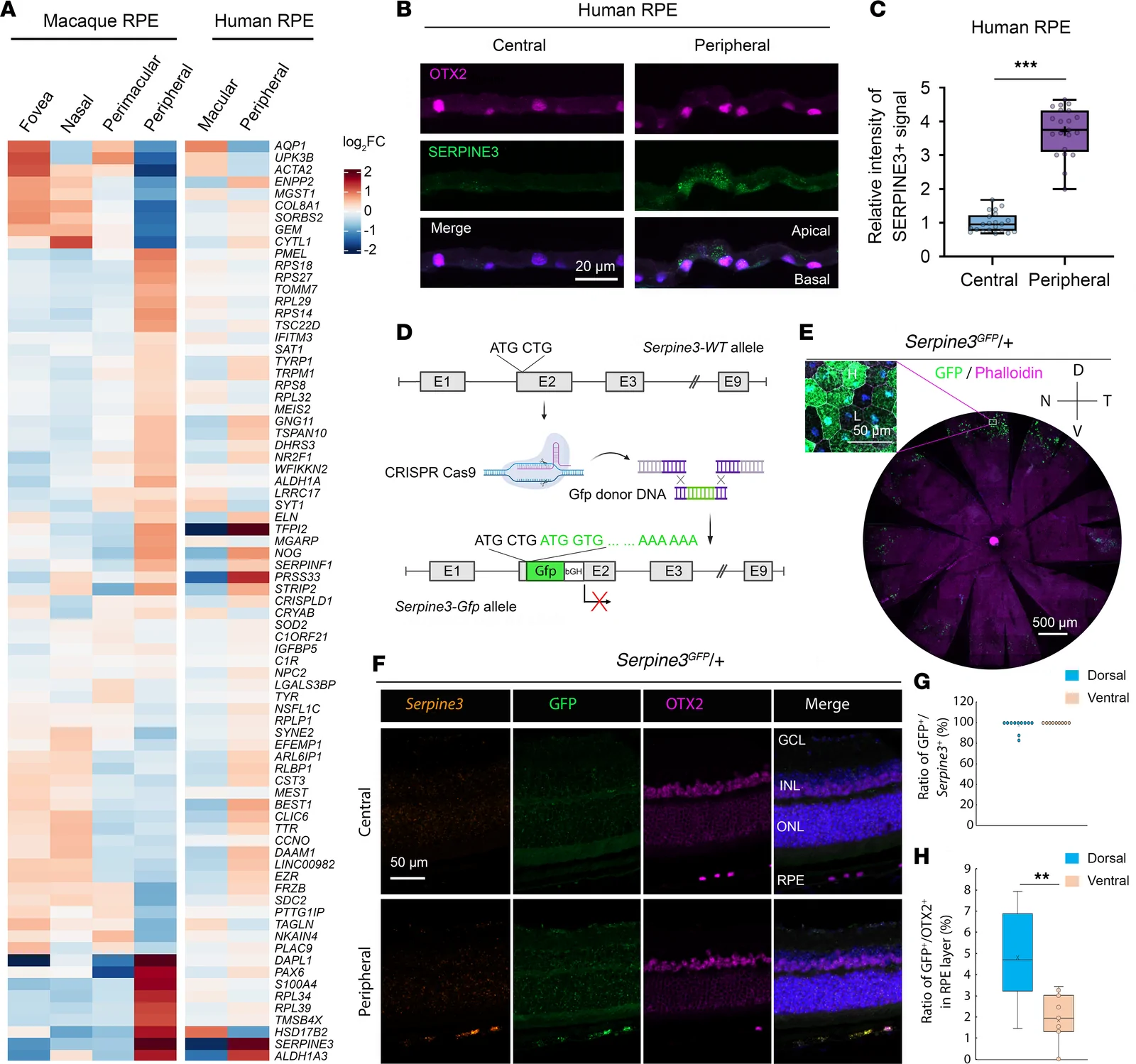
SERPINE3 is highly expressed in the peripheral RPE cells. (**A**) The heatmap of bulk RNA-seq data from different regions of the macaque (left 4 lanes; GSE194285) and human RPE (right 2 lanes; plae.nei.nih.gov). (**B** and **C**) Sectional immunostaining of anti-OTX2 and anti-SERPINE3 in healthy human RPE and the quantification of SERPINE3^+^ signal intensity in the central and peripheral RPE cells (*n* = 5). Scale bar: 20 μm. (**D**) Schematic for generating Serpine3-GFP–knockin mouse model. (**E**) RPE flat-mount immunostaining of anti-GFP combined with phalloidin staining in the *Serpine3^GFP/+^* mice at 2 months (*n* = 5). Scale bars: 500 μm, 50 μm (zoom). H, high level of GFP signal; L, low level of GFP signal. (**F**) Sectional immunostaining of anti-GFP and anti-OTX2 combined with RNAscope of *Serpine3* probe in the central and peripheral RPE of *Serpine3^GFP/+^* mice at 2 months (*n* = 5). Scale bar: 50 μm. (**G** and **H**) Bar graphs show the ratio of GFP^+^ cells among the *Serpine3^+^* cells (**G**) and the ratio of GFP^+^ cells among the OTX2^+^-RPE cells (**H**) in the retinas of *Serpine3^GFP/+^* mice at 2 months. The box-and-whisker plots depict the minimum and maximum values (whiskers), the upper and lower quartiles, and the median. Data are presented as the mean ± SD. Statistics were calculated by unpaired 2-tailed Student’s *t* test (**C**, **G**, and **H**). **P < 0.01, ***P < 0.001. D, dorsal; N, nasal; V, ventral; T, temporal. GCL, ganglion cell layer; INL, inner nuclear layer; ONL, outer nuclear layer.

To understand RPE heterogeneity at the molecular level, we previously conducted a transcriptomic analysis of adult macaque RPE cells and found significant differences in location-specific RPE gene signatures (35). To capture spatial dimensions, in this study, we analyzed the transcriptomic data along the peripheral to the central axis. We discovered that over 20 genes were enriched in the peripheral RPE cells from macaques and humans (Figure 1A). In macaques, *TSPAN10*, *NOG*, *ALDH1A3*, *PMEL17*, *DAPL1*, and *SERPINE3* genes were consistently upregulated, whereas *ELN*, *NOG*, *ALDH1A3*, and *SERPINE3* were found to be enriched in human peripheral RPE cells (plae.nei.nih.gov). Among these genes, SERPINE3 is highly expressed in the peripheral RPE of both humans and macaques. To confirm this expression profile, we analyzed additional omics data of human RPE (plae.nei.nih.gov, SRP080886 and SRP098761) and found that SERPINE3 is consistently enriched in the peripheral RPE (Supplemental Figure 1A; supplemental material available online with this article; https://doi.org/10.1172/JCI191272DS1) (32, 36), underscoring a regional and heterogeneous expression pattern in RPE subpopulations.

To determine if SERPINE3-expressing cells are localized to the peripheral retina, we analyzed SERPINE3 expression in human eye sections. Immunostaining data revealed that the RPE transcription factor OTX2 was expressed in nuclei of both central and peripheral RPE cells (Figure 1, B and C), while SERPINE3 was detected mainly in the cytoplasm of peripheral RPE cells (Figure 1, B and C). Furthermore, to trace SERPINE3^+^ cells, we established a GFP reporter by inserting it mono-allelically just downstream of the start codon in exon 2 of the *SERPINE3^+^* gene in mice (Figure 1D). Reporter knockin was confirmed using GFP-specific and *Serpine3* gene sequence–specific PCR primer pairs (Supplemental Figure 1, B and C). In agreement with RNA-seq data, GFP was detected in the *Serpine3^GFP/+^* RPE but not in the neuroretina at 2 months by Western blot analysis (Supplemental Figure 1D). This pattern of expression was consistent with GFP immunostaining on mouse eye sections showing GFP expression in RPE cells marked with RPE65 immunostaining (Supplemental Figure 1, E and F). *Serpine3^GFP/+^* mice RPE flat-mount analysis showed an enrichment of GFP signal predominantly in zones 4 and 5, located 1.5 mm from the center of the flat mounts in the dorsal peripheral region (Figure 1E and Supplemental Figure 1, G and H), but the level of GFP expression in peripheral RPE cells is highly variable, encompassing high, low, and undetectable levels (Supplemental Figure 1, I and J). The RNAscope data confirmed that *Serpine3* is highly expressed in the peripheral GFP^+^-RPE cells in *Serpine3^GFP/+^* mice (Figure 1, F and G), and the Serpine3^+^ RPE cells account for approximately 5% of total OTX2^+^ RPE cells (Figure 1, F–H).

Collectively, these data reveal Serpine3 as a gene specific to an RPE subpopulation, with predominant expression in a portion of peripheral RPE cells, particularly in the dorsal periphery of the mouse eye. Using a GFP reporter gene, we successfully labeled this Serpine3-expressing RPE subpopulation (hereafter termed S3^+^-RPE) for cell type–specific mapping and in vivo phenotyping.

### The S3^+^-RPE subpopulation has specific transcriptomic features.

**Figure 2:**
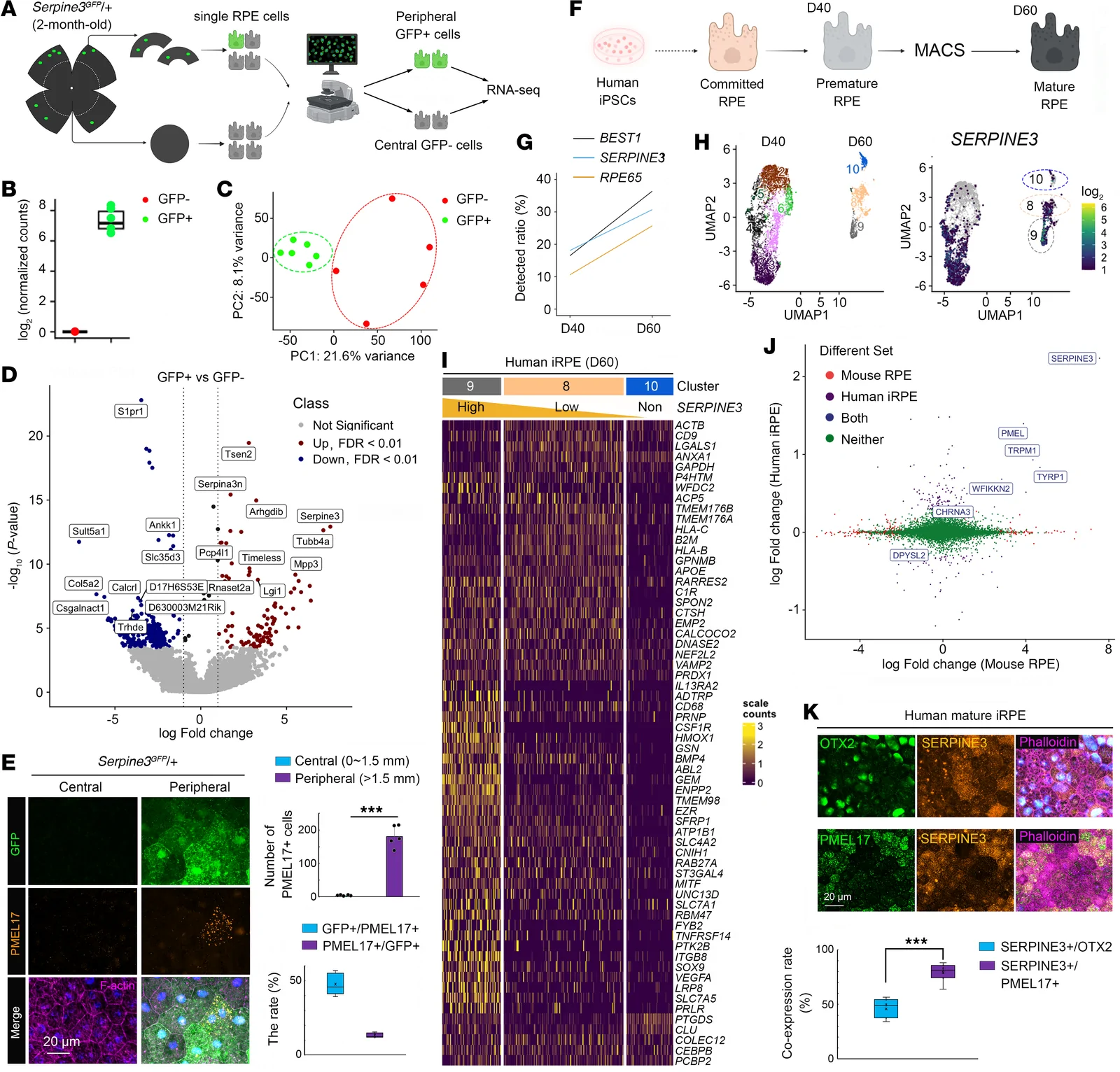
Identifying the transcriptomic features of SERPINE3^+^-RPE cells from 2-month-old *Serpine3^GFP/+^* mice and human iRPE cells. (**A**) The schematic diagram shows the smart scRNA-seq analysis process for the GFP^+^-RPE cells. The RPE cells were divided into peripheral GFP^+^-RPE cells and the central GFP^–^-RPE cells. (**B**) The expression of *Serpine3* in the central GFP^–^-RPE cells and peripheral GFP^+^-RPE cells. (**C**) Principal component analysis dot plots for the smart RNA-seq data from the central GFP^–^-RPE cells and peripheral GFP^+^-RPE cells. (**D**) Volcano dot plots of DEGs show the global transcriptomic changes between central GFP^–^-RPE cells and peripheral GFP^+^-RPE cells. (**E**) Flat-mount immunostaining of anti-PMEL17 and anti-GFP in the RPE from 2-month-old *Serpine3^GFP/+^* mice (left panels), the quantification of PMEL17^+^ cells in the peripheral and central RPE (right upper panel), and the rate of GFP^+^ cells and PMEL17^+^ cells (right lower panel). Scale bar: 20 μm. (**F**) The schematic shows a human iRPE culture system. The premature (day 40) and mature (day 60) iRPE cells were subjected to scRNA-seq analysis. (**G**) The bar graph shows the detection ratio of RPE-specific markers and SERPINE3 in the premature and mature iRPE cells. (**H**) UMAP dot plots show 10 different clusters and SERPINE3 expression in clusters of iRPE cells. (**I**) Heatmap of DEGs in clusters 8–10 of mature iRPE cells. (**J**) Dot plots of DEGs from the combination analysis of human iRPE cells and the GFP^+^-RPE cells. (**K**) Immunostaining images of anti-SERPINE3 and anti-OTX2 or anti-PMEL17 in the mature iRPE cells (upper row) and the coexpression rate of SERPINE3 with OTX2 or PMEL17 in iRPE cells (lower row). Scale bar: 20 μm. The box-and-whisker plots depict the minimum and maximum values (whiskers), the upper and lower quartiles, and the median. Data are presented as mean ± SD. Statistics were calculated by unpaired 2-tailed Student’s *t* test (**B** and **E**) or 2-way ANOVA followed by Tukey’s test (**K**). ***P < 0.001.

To characterize the S3^+^-RPE cells, we utilized smart RNA-seq as an unbiased approach to identify the transcriptome of the S3^+^-RPE cells. Single GFP-positive RPE (GFP^+^-RPE) cells were manually isolated from dissociated peripheral RPE of *Serpine3^GFP/+^* mice at 2 months and then subjected to smart RNA-seq analysis; central GFP-negative RPE (GFP^–^-RPE) cells were used as control (Figure 2A). The RNA-seq data further confirmed high *Serpine3* expression in GFP^+^-RPE cells but not in the central non-GFP group, confirming their *Serpine3* identity (Figure 2B). Principal component analysis revealed that the transcriptome of GFP^+^-RPE cells is significantly distinguishable and less variable compared with the central non-GFP samples (Figure 2C), supporting that GFP^+^-RPE cells represent a peripheral RPE subpopulation characterized by a distinct transcriptomic profile.

Subsequently, we analyzed the enriched genes either in the peripheral GFP^+^-RPE cells or GFP^–^-RPE cells. As shown in Figure 2D, hundreds of genes were highly expressed in the peripheral GFP^+^-RPE cells, including RPE maturational genes *Pmel17* and *Tyrp1* (Figure 2D). Immunostaining of RPE flat mounts confirmed that GFP signal in the far periphery overlaps with the PMEL17 signal in 2-month-old *Serpine3^GFP/+^* mice (Figure 2E). Immunostaining of mouse eye sections showed that while PMEL17 was detectable in the central and peripheral RPE cells at E14.5, it could only be detected in the peripheral RPE at 2 months (Supplemental Figure 2A), suggesting a different maturation state of peripheral RPE cells. GSEA of the transcriptome data revealed signaling pathways that were enriched in GFP^+^-RPE cells, for instance, ionotropic glutamate G protein–coupled receptor signaling pathways had higher expression of suppressors, whereas the interferon-related signaling pathway had higher expression of activators in GFP^+^-RPE cells (Supplemental Figure 2B). In addition, CNet plots of clusters of differentially expressed genes (DEGs) showed alterations in genes associated with signal receptor activity, including the IL-17RE–mediated inflammation pathway and Trpm1-mediated metal ion transmembrane transporter pathway, which are predominantly expressed in GFP^+^-RPE cells (Supplemental Figure 2C). These results indicate that S3^+^-RPE cells, a peripheral RPE subpopulation, possess a transcriptomic profile distinct from that of central RPE cells and may exhibit differential responses to inflammatory stimuli.

To identify transcriptomic features of this subpopulation in human RPE, we used our induced pluripotent stem cells–derived RPE (iRPE) system. Our previously published iRPE differentiation protocol (36, 37) generates a mixed population of RPE cells, within which we assumed SERPINE3^+^ cells would form 1 subpopulation. To investigate this, we conducted scRNA-seq analysis at different stages of differentiation (Figure 2F). Notably, we found that the detected ratio of SERPINE3, similar to known mature RPE markers *BEST1* and *RPE65*, increased in mature iRPE cells compared with premature iRPE. This suggests high SERPINE3 expression in an RPE subpopulation at a later stage (Figure 2G). UMAP data revealed 6 population clusters in premature iRPE cells and 3 in mature iRPE cells. SERPINE3 expression was highest in cluster 9 of mature iRPE cells (Figure 2H). The expression profile of SERPINE3^+^ iRPE cells was closer to published human peripheral RPE cells than to macular RPE cells (Supplemental Figure 2D). This suggests that SERPINE3^+^ cells in our iRPE population are likely candidates for peripheral RPE cells. We compared clusters 9, 8, and 10, which ranged from the highest to the lowest SERPINE3 expression. Cluster 9 was enriched in antioxidant and inflammation genes, such as *NEF2L2*, *HMOX1*, and *TNFRSF14* (Figure 2I). This result suggests that SERPINE3^+^-iRPE cells may have distinctive oxidative stress and inflammatory responses compared with other iRPE subpopulations. Combined analysis of RNA-seq data from human cluster 9 iRPE cells and mouse GFP^+^-RPE cells revealed genes enriched in both, including *SERPINE3*, *PMEL17*, and *TYRP1* (Figure 2J). This further supports the similarity of their lineages. Immunostaining showed that SERPINE3^+^ cells were coexpressed more frequently with PMEL17 than with OTX2 in mature human iRPE cells (Figure 2K). Additionally, nuclear NRF2 enrichment was observed in the peripheral RPE of both WT and *Serpine3^GFP/+^* mice, but not in the central RPE of either genotype (Supplemental Figure 3), indicating that nuclear NRF2 enrichment is a feature of the peripheral RPE, a region where SERPINE3-expressing cells reside.

Collectively, based on the transcriptomic analysis in mouse and human RPE cells, SERPINE3 is expressed at a later differentiation stage and is coexpressed with PMEL17 and NRF2 in a small subpopulation of RPE cells that may have differential sensitivity to oxidative stress and inflammatory responses.

### The S3^+^-RPE subpopulation resists oxidative stress.

**Figure 3:**
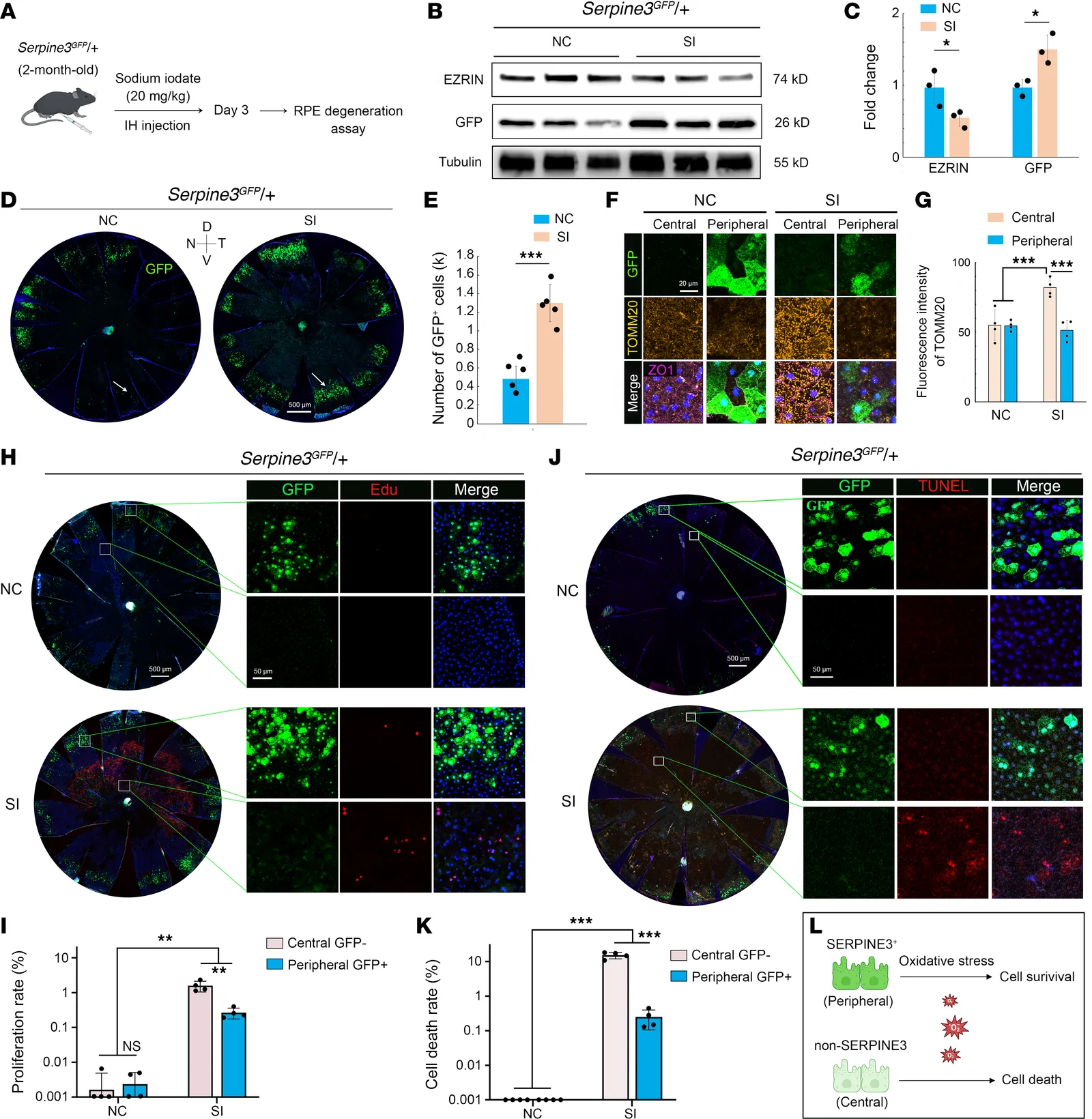
The responses of GFP^+^-RPE cells to SI injury in 2-month-old *Serpine3^GFP/+^* mice. (**A**) Schematic of SI-induced RPE degeneration in *Serpine3^GFP/+^* mice at 2 months. (**B** and **C**) Western blotting (**B**) and quantification (**C**) show EZRIN and GFP expression in the RPE with or without SI treatment in *Serpine3^GFP/+^* mice at 2 months (*n* = 3). (**D**) RPE flat-mount immunostaining images of anti-GFP in the *Serpine3^GFP/+^* mice under normal (left panel) or SI-treated conditions (right panel). Scale bar: 500 μm. (**E**) Bar graphs show increased GFP^+^-RPE cells in the *Serpine3^GFP/+^* mice after SI injury (*n* = 5). (**F** and **G**) RPE flat-mount immunostaining images of anti-TOMM20 in the central and peripheral RPE of *Serpine3^GFP/+^* mice with or without SI treatment (**F**) and the quantification of fluorescence intensity of TOMM20 (**G**). Note: the central RPE was analyzed at the transition zone under the SI treatment condition. Scale bar: 20 μm. (**H** and **I**) RPE flat-mount immunostaining images of anti-GFP with EdU staining in the *Serpine3^GFP/+^* mice under indicated conditions (**H**) and quantification of proliferation rate in the central GFP^–^-RPE cells or peripheral GFP^+^-RPE cells (**I**). Scale bars: 500 μm, 50 μm (zoom). (**J** and **K**) Immunostaining images of anti-GFP with TUNEL staining in the *Serpine3^GFP/+^* mice (**J**) and quantification of cell death rate (**K**) under indicated conditions (*n* = 5). Scale bars: 500 μm, 50 μm (zoom). (**L**) Schematic summarizes the different sensitivity between SERPINE3^+^ and SERPINE3^–^-RPE cells to oxidative stress. D, dorsal; N, nasal; V, ventral; T, temporal. Data are presented as mean ± SD. Statistics were calculated by unpaired 2-tailed Student’s *t* test (**C** and **E**) or 2-way ANOVA followed by Tukey’s test (**G**, **I**, and **K**). *P < 0.05, **P < 0.01, ***P < 0.001.

To understand how S3^+^-RPE cells respond to inflammation and oxidative stress, we used a 3-day sodium iodate–induced (SI-induced) RPE injury model to detect early RPE damaged events (Figure 3A). After 3 days of SI treatment in *Serpine3^GFP/+^* 2-month-old mice, we found by Western blot analysis that the GFP protein levels increased by 40%, whereas expression of the RPE-specific marker EZRIN decreased by 30% (Figure 3, B and C). Consistently, in iRPE cells, SERPINE3 expression increased by 5-fold, whereas EZRIN decreased to about half after SI (10 mM) treatment for 1 day (Supplemental Figure 4, A–C). Interestingly, analysis of SI-treated mouse RPE flat mounts showed an approximately 3-fold increase in GFP signal, forming a relatively complete ring in the peripheral RPE (Figure 3, D and E). In contrast, SI significantly altered RPE tight junctions (as analyzed by ZO1 immunostaining) and typical RPE hexagonal morphology in the dorsal central area (Supplemental Figure 4D). It also reduced the number of RPE cells by approximately 25% that could be counted in this region (Supplemental Figure 4E). Furthermore, under normal physiological conditions, the oxidative stress marker 4-hydroxynonenal is highly expressed in the central RPE, but upon SI-induced injury, it becomes barely detectable in the central RPE, whereas the lower expression of 4-hydroxynonenal in the peripheral GFP^+^-RPE remains largely unaffected by the SI injury (Supplemental Figure 4, F and G), suggesting that peripheral GFP^+^-RPE cells are less sensitive to oxidative stress. Consistently, the protein level of TOMM20, an indicator of mitochondrial abundance and network structure, was increased by approximately 50% in the transition zone RPE but not in the GFP^+^-RPE cells after treatment with SI (Figure 3, F and G). These results suggest that concomitant with increased *Serpine3* expression in response to SI injury, the S3^+^-RPE subpopulation is resistant to this injury.

Given the expansion of GFP signal after SI treatment, we next wondered whether the increase of GFP^+^ cells was partially due to GFP^+^-RPE cell proliferation. To answer this question, we used EdU to analyze RPE cell proliferation and found that the EdU signal was almost undetectable in the entire RPE monolayer in 2-month-old *Serpine3^GFP/+^* mice without SI injury (Figure 3H). After treatment with SI, both GFP^+^ and GFP^–^-RPE cells became EdU^+^ in the dorsal peripheral region, suggesting that RPE cells can reenter the cell cycle (Figure 3, H and I). Further analysis revealed that GFP^–^-RPE cells that were EdU^+^ did not express the RPE marker OTX2 but rather expressed the key epithelial-to-mesenchymal transition marker Vimentin, whereas GFP^+^-RPE cells robustly expressed OTX2 and not Vimentin (Supplemental Figure 4, H and I), suggesting that central RPE cells may be losing their RPE fate as they proliferate in response to injury, whereas peripheral SERPINE3^+^-RPE cells continue to retain their RPE fate. To understand why the peripheral RPE cells are preserved after SI treatment, we next assessed if GFP^+^-RPE cells evade cell death. We found that the apoptotic rate of GFP^+^-RPE cells was slightly increased due to the injury of SI, whereas the apoptotic rate of central GFP^–^-RPE cells was elevated by about 50-fold (Figure 3, J and K). These results suggest that S3^+^-RPE cells reenter the cell cycle and avoid apoptosis in response to SI injury (Figure 3L).

### SERPINE3 is required for S3^+^-RPE cells to resist oxidative stress.

**Figure 4:**
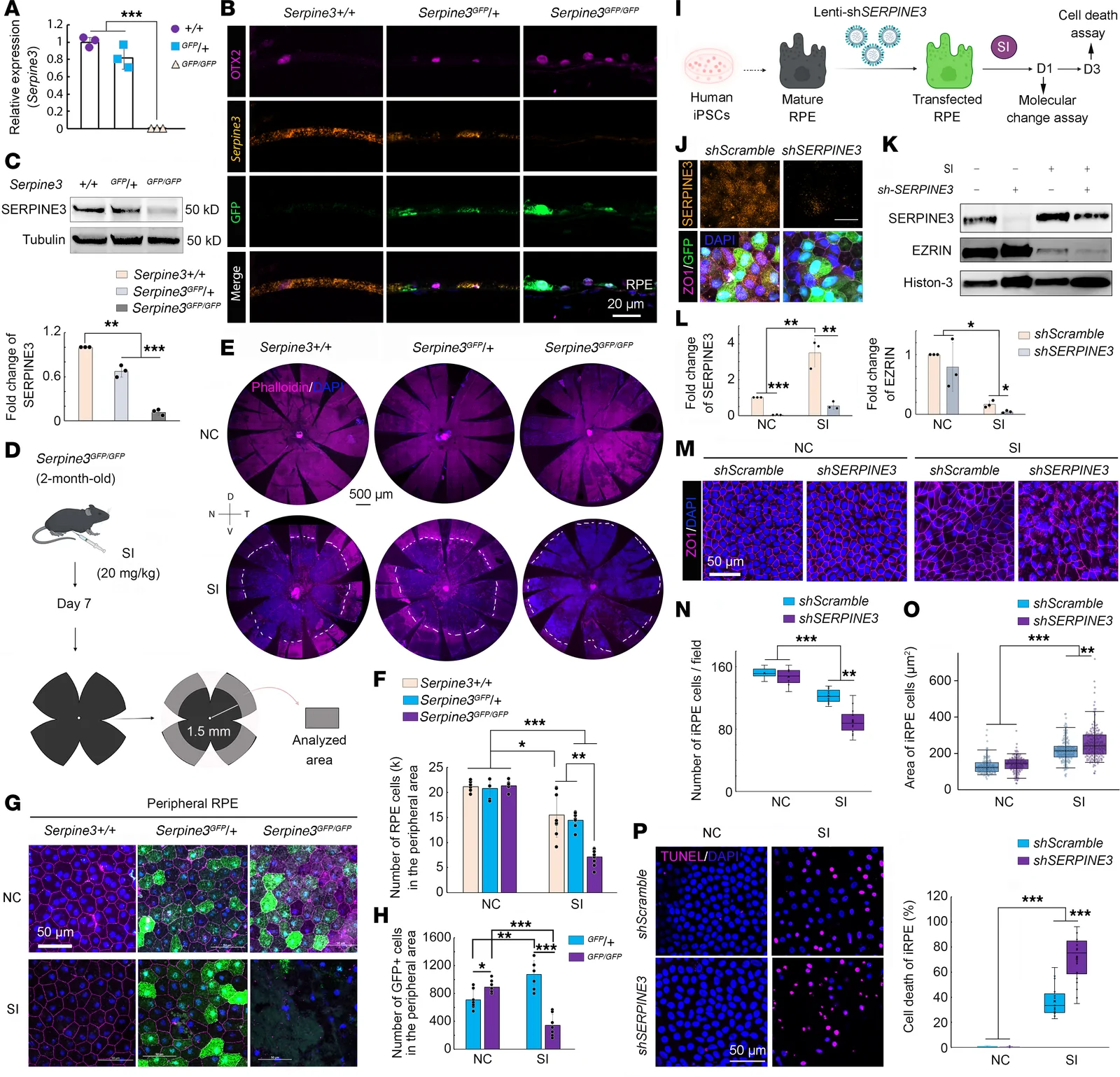
Loss of SERPINE3 accelerates SI-induced RPE degeneration. (**A**) qPCR shows *Serpine3* levels in the RPE/choroid tissue of WT, *Serpine3^GFP/+^*, and *Serpine3^GFP/GFP^* mice at 2 months. (**B**) Sectional RNAscope of *Serpine3* probe with immunostaining of anti-OTX2 in the RPE from the 3 genotypes at 2 months (*n* = 5). Scale bar: 20 μm. (**C**) Western blotting (upper panels) and corresponding quantification (lower panels) reveal decreased SERPINE3 in the RPE of *Serpine3^GFP/GFP^* mice (*n* = 3). (**D**) Schematic of SI-induced RPE degeneration in the *Serpine3^GFP/GFP^* mice at 2 months. (**E** and **F**) Phalloidin staining images of flat-mount RPE (**E**) and quantification of peripheral RPE cell counts in all genotypes of mice, with or without SI treatment (**F**). Note: the area of RPE damage is delineated by the white dashed line. Scale bar: 500 μm. (**G** and **H**) RPE flat-mount immunostaining images of anti-ZO1 and anti-GFP (**G**) and quantification of GFP^+^ peripheral RPE cells (**H**) under the indicated conditions (*n* = 5). Scale bar: 50 μm. (**I**) Schematic of SI injury in human iRPE cells with lenti-shSERPINE3. (**J**) Immunostaining of anti-SERPINE3 in iRPE cells with lenti-shSERPINE3. Scale bar: 20 μm. (**K** and **L**) Western blotting (**K**) and quantification (**L**) of SERPINE3 and EZRIN expression in iRPE cells under the indicated conditions. (**M**–**O**) Immunostaining images of anti-ZO1 in the iRPE cells (**M**) and the quantification of total (**N**) and single-area (**O**) RPE cells number under the indicated conditions. Scale bar: 50 μm. (**P**) TUNEL staining (left panels) and quantification of TUNEL^+^ iRPE cells under the indicated conditions (right panel). Scale bar: 50 μm. The box-and-whisker plots depict the minimum and maximum values (whiskers), the upper and lower quartiles, and the median. Data are presented as mean ± SD. Statistics were calculated by 2-way ANOVA followed by Tukey’s test (**A**, **C**, **F**, **G**, **L**, and **N**–**P**). *P < 0.05, **P < 0.01, ***P < 0.001.

To understand whether S3^+^-RPE cells resist SI injury through SERPINE3, we crossed *Serpine3^GFP/+^* mice to obtain homozygous *Serpine3^GFP/GFP^* mice in which the Serpine3 transcript is expected to be replaced by the GFP transcript due to the bovine growth hormone terminator inserted after the GFP coding sequence (Figure 1C). Indeed, qPCR, RNAscope, Western blot analysis, and immunostaining consistently showed that the mRNA and protein levels of SERPINE3 were both markedly decreased in the RPE of *Serpine3^GFP/GFP^* mice at 2 months (Figure 4, A–C, and Supplemental Figure 5A). Expectedly, the GFP^+^-RPE cells were enriched in the dorsal periphery RPE in *Serpine3^GFP/GFP^* mice similar to *Serpine3^GFP/+^* mice (Supplemental Figure 5B). Additionally, retinal vasculature (Supplemental Figure 5C), RPE pigmentation, retinal thickness (Supplemental Figure 5, D and E), and retinal function (Supplemental Figure 5, F–I) of *Serpine3^GFP/GFP^* mice were all similar to age-matched WT or *Serpine3^GFP/+^* mice. These data indicate that Serpine3 is either not required or its low level is sufficient for retina development and maintenance under normal physiological conditions in young mice.

Next, we injected SI into the *Serpine3^GFP/GFP^* mice and analyzed late events of RPE damage at day 7 (Figure 4D). After treatment with SI, phalloidin signal was almost undetectable in the central RPE in WT, *Serpine3^GFP/+^*, and *Serpine3^GFP/GFP^* mice, while the peripheral phalloidin signal was mainly preserved in WT and *Serpine3^GFP/+^* mice but almost undetectable in *Serpine3^GFP/GFP^* mice (Figure 4E), suggesting that deletion of Serpine3 accelerates SI injury in the peripheral RPE. Quantification of these data showed an approximately 5% decrease in the number of RPE cells in the injured WT and *Serpine3^GFP/+^* RPE but a 3-fold greater decrease in the *Serpine3^GFP/GFP^* RPE (Figure 4F). Consistently, in the peripheral area, GFP^+^-RPE cells maintained normal hexagon shape in the *Serpine3^GFP/+^* mice but had lost their hexagonal morphology and presented an enlarged fibrous shape in the *Serpine3^GFP/GFP^* mice after SI treatment (Figure 4G). Quantification of GFP^+^ cells showed 2-fold higher cell death in *Serpine3^GFP/GFP^* peripheral RPE compared with *Serpine3^GFP/+^* peripheral RPE (Figure 4H). Collectively, these data suggest that the S3^+^-RPE subpopulation resists oxidative stress in a SERPINE3-dependent manner.

To determine the role of SERPINE3 in human RPE cells, we knocked it down using the lenti-shSERPINE3 virus in iRPE cells (Figure 4I). Immunostaining and Western blotting confirmed efficient knockdown (Figure 4, J–L). After SI treatment, compared with controls, SERPINE3 was upregulated and EZRIN was downregulated in lenti-shSERPINE3–transfected iRPE, as observed in mouse RPE cells (Figure 4, K and L). Again, ZO1 immunostaining showed that SI-treated, SERPINE3-deficient iRPE cells lost typical hexagonal morphology and had increased cell size, suggesting epithelial-to-mesenchymal transition initiation, consistent with mouse RPE cells (Figure 4, M–O). Under SI treatment, SERPINE3-knockdown iRPE cells displayed 2-fold higher cell death than controls (Figure 4P). These data indicate SERPINE3 knockdown accelerates oxidative stress–induced RPE damage in human cells.

### Deletion of Serpine3 leads to age-related RPE degeneration and inflammation.

**Figure 5:**
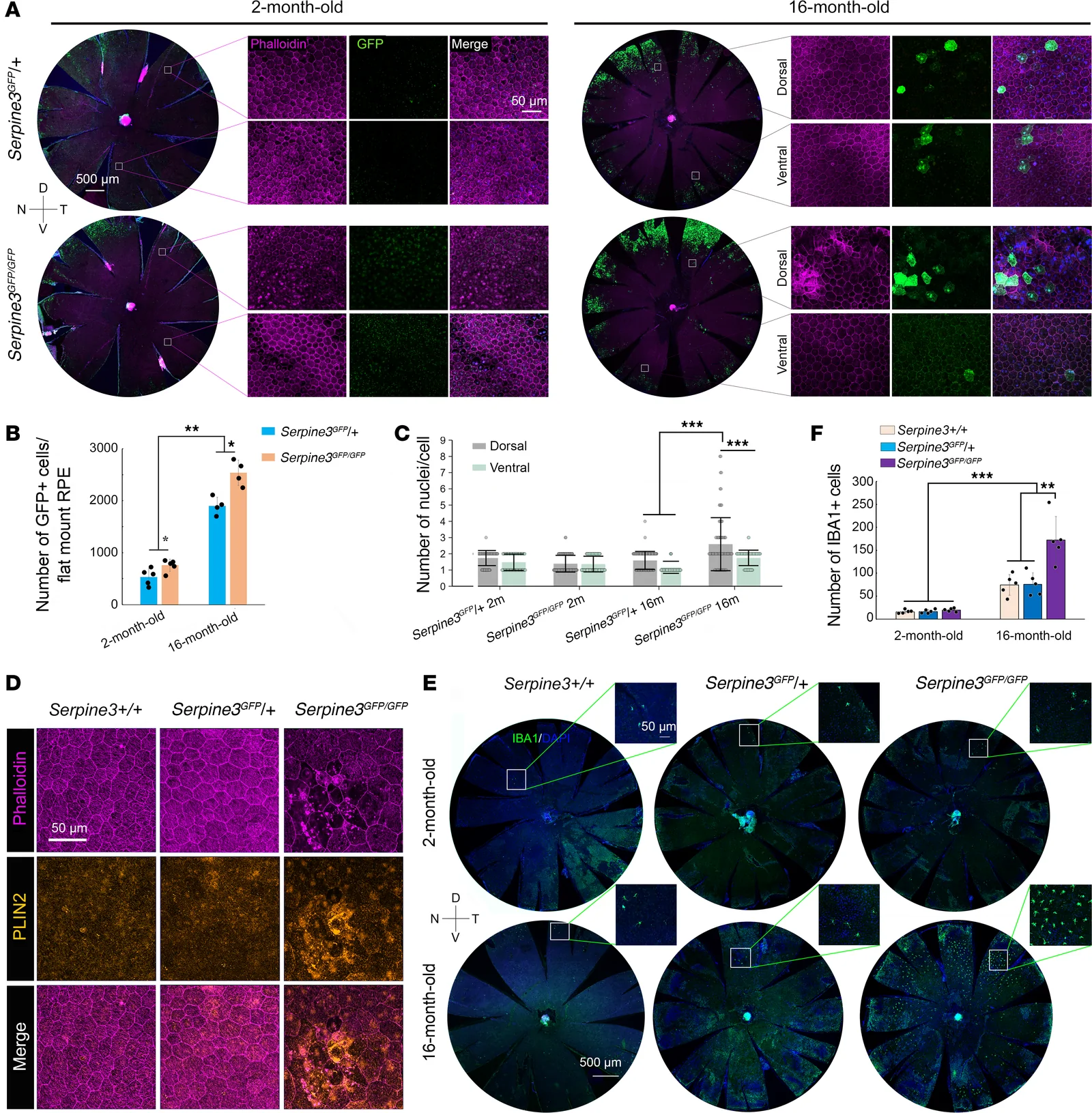
RPE degeneration assay in young and aged *Serpine3^GFP/GFP^* mice. (**A**) RPE flat-mount immunostaining of anti-GFP with phalloidin staining in *Serpine3^GFP/+^* (upper panels) and *Serpine3^GFP/GFP^* (lower panels) mice at 2 months (left panels) or 16 months (right panels). Scale bars: 500 μm, 50 μm (zoom). (**B**) Quantification of GFP^+^ cells shows more GFP^+^-RPE cells in aged *Serpine3^GFP/+^* and *Serpine3^GFP/GFP^* mice (*n* ≥ 4). (**C**) Quantification of the nucleus in each RPE cell from the dorsal retina of *Serpine3^GFP/+^* and *Serpine3^GFP/GFP^* (lower panels) mice at the 2 ages (*n* ≥ 4). (**D**) RPE flat-mount immunostaining of anti-PLIN2 with phalloidin staining in aged WT, *Serpine3^GFP/+^*, and *Serpine3^GFP/GFP^* mice (*n* = 5). Scale bar: 50 μm. (**E**) RPE flat-mount immunostaining of anti-IBA1 in all 3 strains at 2 months (upper panels) or 16 months (lower panels). Scale bars: 500 μm, 50 μm (zoom). (**F**) Quantification shows more IBA1^+^ microglial cells in *Serpine3^GFP/GFP^* aged retinas (*n* = 5). Data are presented as mean ± SD. Statistics were calculated by 2-way ANOVA followed by Tukey’s test (**B**, **C**, and **F**). *P < 0.05, **P < 0.01, ***P < 0.001.

To further understand the roles of Serpine3 in RPE cells, we explored whether Serpine3 plays a role in RPE cells during aging. First, we examined GFP expression in 16-month-old *Serpine3^GFP/+^* mice. We found that GFP was upregulated approximately 6-fold in the aged RPE compared with younger mice (Supplemental Figure 6). We also discovered that IBA1, a marker of microglia, was also slightly upregulated in the RPE tissues of the aged *Serpine3^GFP/+^* mice (Supplemental Figure 6), suggesting their increased migration to the subretinal space (37, 38). We then examined the RPE morphology and inflammation in the aged *Serpine3^GFP/GFP^* mice. We observed that the number of GFP^+^-RPE cells increased by more than 3-fold in aged *Serpine3^GFP/+^* and *Serpine3^GFP/GFP^* mice compared with younger mice. The proportion rose from about 1% to 4% of total RPE cells (Figure 5, A and B). In these aged mice, the GFP^+^ signal expanded to form a contiguous ring in the far peripheral RPE (Figure 5A). At 2 months, both *Serpine3^GFP/+^* and *Serpine3^GFP/GFP^* mice RPE cells displayed the typical hexagonal morphology (Figure 5A). However, the abnormal RPE morphology was observed in the aged *Serpine3^GFP/GFP^* mice but not in the *Serpine3^GFP/+^* mice (Figure 5A). Within the dorsal area of aged *Serpine3^GFP/GFP^* mice, the RPE cell size and number of nuclei increased significantly, while the ventral RPE cells maintained their normal shape and number of nuclei (Figure 5C). In addition, RPE morphometry analysis over the entire flat mount showed a significant decrease in number of neighbors for a given RPE cell, specifically in the dorsal area of aged *Serpine3^GFP/GFP^* RPE tissues (Supplemental Figure 7, A and B). In the dorsal area, PLIN2, a marker of lipid droplets whose long-term accumulation is a sign of RPE metabolic dysfunction, was abnormally deposited at the sub-RPE space only in the aged *Serpine3^GFP/GFP^* mice (Figure 5D and Supplemental Figure 7, C and D), even though phagocytosis of RPE was unchanged among aged WT, *Serpine3^GFP/+^*, and *Serpine3^GFP/GFP^* mice (Supplemental Figure 8), suggesting that deletion of SERPINE3 leads to higher metabolic dysfunction in aged RPE cells. Coincidentally with this finding, we discovered at least a 2-fold higher number of IBA1^+^ cells enriched in the subretinal space, specifically of aged *Serpine3^GFP/GFP^* mice (Figure 5, E and F). In contrast, microglial cells were only slightly increased in the aged WT and *Serpine3^GFP/+^* RPE compared with younger mice (Figure 5, E and F). These data suggested that deletion of Serpine3 leads to regional RPE degeneration and inflammation in aged mice.

### Serpine3 inhibits the activity of caspase-1.

**Figure 6:**
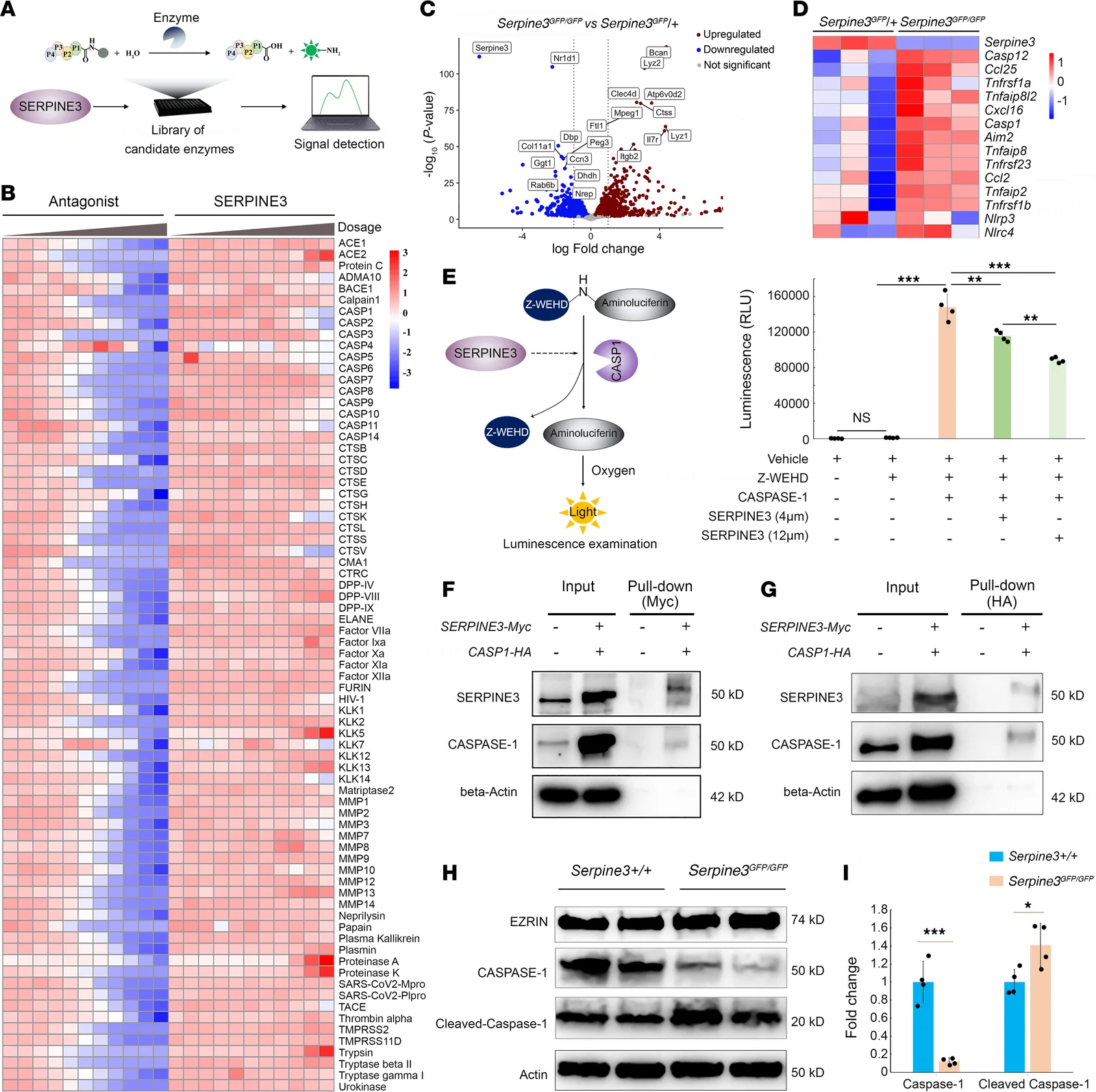
The downstream target assay of SERPINE3 by proteinase screen and RNA-seq analysis. (**A**) Schematic of proteinase screen assay. (**B**) The heatmap shows the activity of candidate enzymes treated by the antagonist (left panels) or recombinant SERPINE3 (right panels) in the proteinase screen system. (**C**) Volcano dot plots of DEGs show the global transcriptomic changes in the RPE/choroid tissue of aged *Serpine3^GFP/GFP^* mice compared with aged *Serpine3^GFP/+^* mice. (**D**) Heatmap shows changes in genes associated with caspase-1 inflammatory pathways in aged *Serpine3^GFP/GFP^* mice at 18 months. (**E**) Caspase-1 inflammasome assay combined with recombinant SERPINE3 treatment. The left panels show the processing of the CASPASE-1 activity assay. The right bar graph shows caspase-1 activity blocked by the SERPINE3. (**F** and **G**) Co-IP for SERPINE3 and CASPASE-1 after MYC (**F**) or HA (**G**) pulldown in the HEK293T cell lines transfected by both SERPINE-MYC and CASPASE-1-HA plasmids. (**H** and **I**) Immunoblotting (**H**) and quantification (**I**) of pro- and cleaved-caspase-1 in the RPE of WT and *Serpine3^GFP/GFP^* mice at postnatal 2 months (*n* = 4). Data are presented as mean ± SD. Statistics were calculated by 2-way ANOVA followed by Tukey’s test (**E** and **I**). *P < 0.05, **P < 0.01, ***P < 0.001.

To understand how SERPINE3 regulates inflammation, we utilized the serine protease inhibition screen to identify its downstream targets (Figure 6A) because SERPINE3 is a member of the SERPIN family of serine protease inhibitors (39). The commercial recombinant SERPINE3 protein was diluted into a gradient and added to an enzyme library consisting of 75 candidate proteases, including members of the chymotrypsin serine protease family, caspase, and cathepsin families of cysteine protease. The heatmap of protease screen inhibitory activity revealed that SERPINE3 significantly inhibited several caspase enzymes, including caspase-1, -2, and -5 (Figure 6B). Among these 3 enzymes, SERPINE3 inhibited the activity of caspase-1 most efficiently with an IC_50_ of 4 nM (Supplemental Figure 9A). To discover which inflammatory pathways are regulated by SERPINE3, with or without caspase-1 inhibition, we performed bulk RNA-seq on RPE cells in aged *Serpine3^GFP/+^* and *Serpine3^GFP/GFP^* mice. The volcano dot plots showed that several genes were significantly changed in the aged *Serpine3^GFP/GFP^* mice (Figure 6C), and Gene Ontology data revealed potential activation of inflammatory pathways (Supplemental Figure 9B). Specifically, in aged *Serpine3^GFP/GFP^* mice, in addition to the upregulation of caspase-1, TNF receptor genes *Tnfrsf1a* and *Tnfrsf1b* were both upregulated; the inflammasome gene *Aim2* was also significantly upregulated, but there was no change in the expression of the other 2 inflammasome genes *Nlrp3* and *Nlrc4* (Figure 6D). These results suggest that deletion of *Serpine3* leads to activation of the TNF/caspase-1/AIM2 inflammatory pathway in the RPE during aging.

Caspase-1 is a critical component of the canonical inflammasomes known to trigger RPE degeneration (40, 41). Therefore, we further analyzed the inhibitory relationship between caspase-1 and SERPINE3 by caspase-1 inflammasome assay (Figure 6E). Recombinant caspase-1 successfully catalyzed the substrate Z-WEHD-aminoluciferin to generate luminescence, and this reaction was significantly blocked by recombinant SERPINE3 (Figure 6E). These results confirmed that SERPINE3 inhibits caspase-1 activity in this context.

To better understand the mechanism of SERPINE3-mediated caspase-1 inhibition, we explored whether SERPINE3 directly binds to caspase-1. To this aim, we overexpressed SERPINE3-MYC and CASPASE-1–HA in the HEK293T cell line and performed co-IP. The data revealed that the Myc antibody successfully pulled down not only the tagged SERPINE3 protein but also the CASPASE-1 protein (Figure 6F). Similarly, when we use the HA antibody, both SERPINE3 and tagged CASPASE-1 protein were successfully pulled down (Figure 6G). These data further supported a direct interaction between SERPINE3 and caspase-1.

To understand whether SERPINE3 regulates caspase-1 in vivo, we examined the protein level of caspase-1 in the RPE of aged *Serpine3^GFP/GFP^* mice. We found that pro-caspase-1 was robustly decreased, but cleaved-caspase-1 was significantly increased in the aged *Serpine3^GFP/GFP^* RPE tissues compared with the aged-matched WT RPE tissues (Figure 6, H and I). This result supported the idea that the deletion of *Serpine3* accelerates catalysis of caspase-1 to generate cleaved-caspase-1. Furthermore, the downstream effector of the caspase-1 pathway, IL-18, was also increased 2.5-fold in the aged *Serpine3^GFP/GFP^* mice compared with aged WT or *Serpine3^GFP/+^* mice (Supplemental Figure 9, C and D). Collectively, our data suggest that the TNF/caspase-1/AIM2/IL-18 inflammation pathway is activated in Serpine3-deficient RPE cells during aging, and SERPINE3 block caspase-1 activity targets, likely by directly binding to it.

### The reactive center loop is crucial for SERPINE3 to block caspase-1.

**Figure 7:**
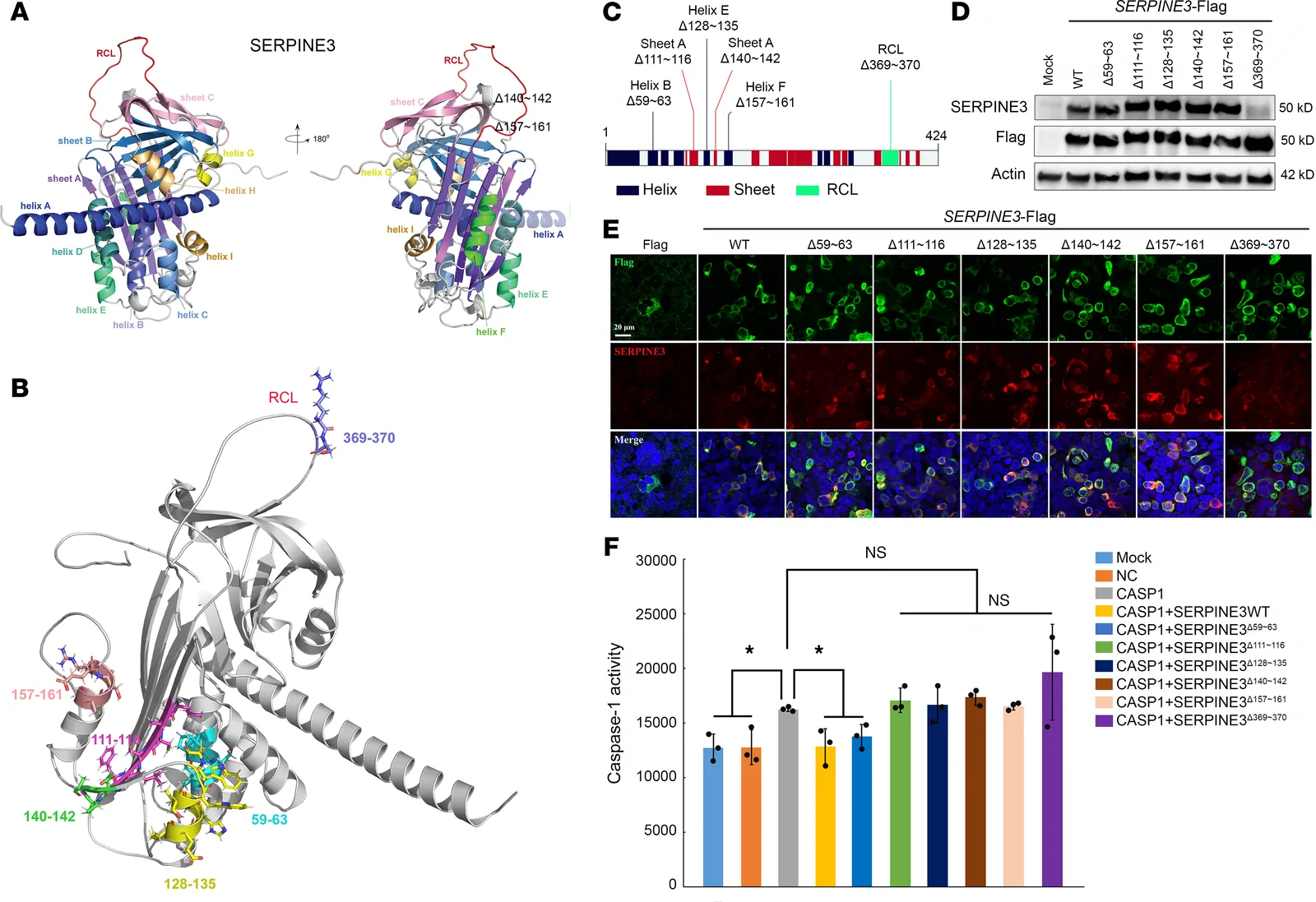
Protein functional domain assay of SERPINE3. (**A** and **B**) The predicted protein structure (**A**) and functional domains (**B**) of SERPINE3 through AlphaFold2. (**C**) Schematic of *SERPINE3* mutants in the functional domains. (**D** and **E**) Immunostaining (**D**) and immunoblotting (**E**) for SERPINE3 and Flag in the HEK293T cell lines transfected by the WT SERPINE3-Flag or 6 different mutants of SERPINE3-Flag. Scale bar: 20 μm. (**F**) CASPASE-1 activity assay in the HEK293T cells transfected by the CASPASE-1 and mutant SERPINE3-Flag plasmids (*n* = 3). Data are presented as mean ± SD. Statistics were calculated by 2-way ANOVA followed by 2-tailed Mann-Whitney *U* test (**F**). *P < 0.05.

We next sought to determine the functional domains of SERPINE3 that may be required for its inhibitory activity. The predicted protein structure of SERPINE3 was generated according to publicly available sequences (accession number NP_001094790.1) using AlphaFold2. Its predicted structure showed an evolutionarily conserved topology of serpins, consisting of 3 β-sheets, 9 α-helices, and 1 reactive center loop (RCL) (Figure 7A). The predicted protein structure of SERPINE3 was then aligned to the known protein structure of SERPINE1 (PDB: 1DVM, chain A) to further analyze its functional domains (Supplemental Figure 10). The root mean square deviation (RMSD) between the SERPINE1 protein structure and the predicted SERPINE3 structure is < 2 (1.740) when calculated using the “super” command in PyMOL, suggesting that these 2 structures are very similar and that SERPINE3 AlphaFold prediction was creditable. Based on the protein structure data of SERPINE1 (42–44), 6 regions of SERPINE3 were predicted to be putative reactive sites, including the helix B, helix E, helix F, 2 sites of sheet A, and the RCL (Figure 7B).

To confirm this prediction, we deleted the sequence of these 6 sites in the human SERPINE3 gene (Figure 7C) and transfected them into the HEK293T cell lines individually. The immunostaining data revealed that only 5 of the 6 mutant SERPINE3 proteins could be detected using our SERPINE3 antibody. The remaining modified SERPINE3 protein (Δ369~370), which had a mutation in the RCL region, was not detectable using this antibody (Figure 7D). However, the Flag-tagged protein was detectable in cells transfected with the RCL mutant SERPINE3 (Figure 7D). Consistently, our Western blot data also showed a similar result (Figure 7E). These findings suggest that all 6 mutations do not affect the protein level, but the mutation in the RCL decreases the reactivity of SERPINE3 with the specific antibody. Next, we analyzed whether the mutations affect SERPINE3’s ability to inhibit CASPASE-1. The caspase-1 activity assay showed that WT SERPINE3 successfully inhibited the activity of caspase-1, and 5 of 6 SERPINE3 mutants failed to reduce the activity of caspase-1, especially the mutation in the RCL domain (Figure 7F). These results indicate that the predicted sites are significant for the function of SERPINE3, especially the RCL site.

### Pharmacological treatment of caspase-1 inhibitor recuses SERPINE3-deficient iRPE cell death under oxidative stress conditions.

**Figure 8:**
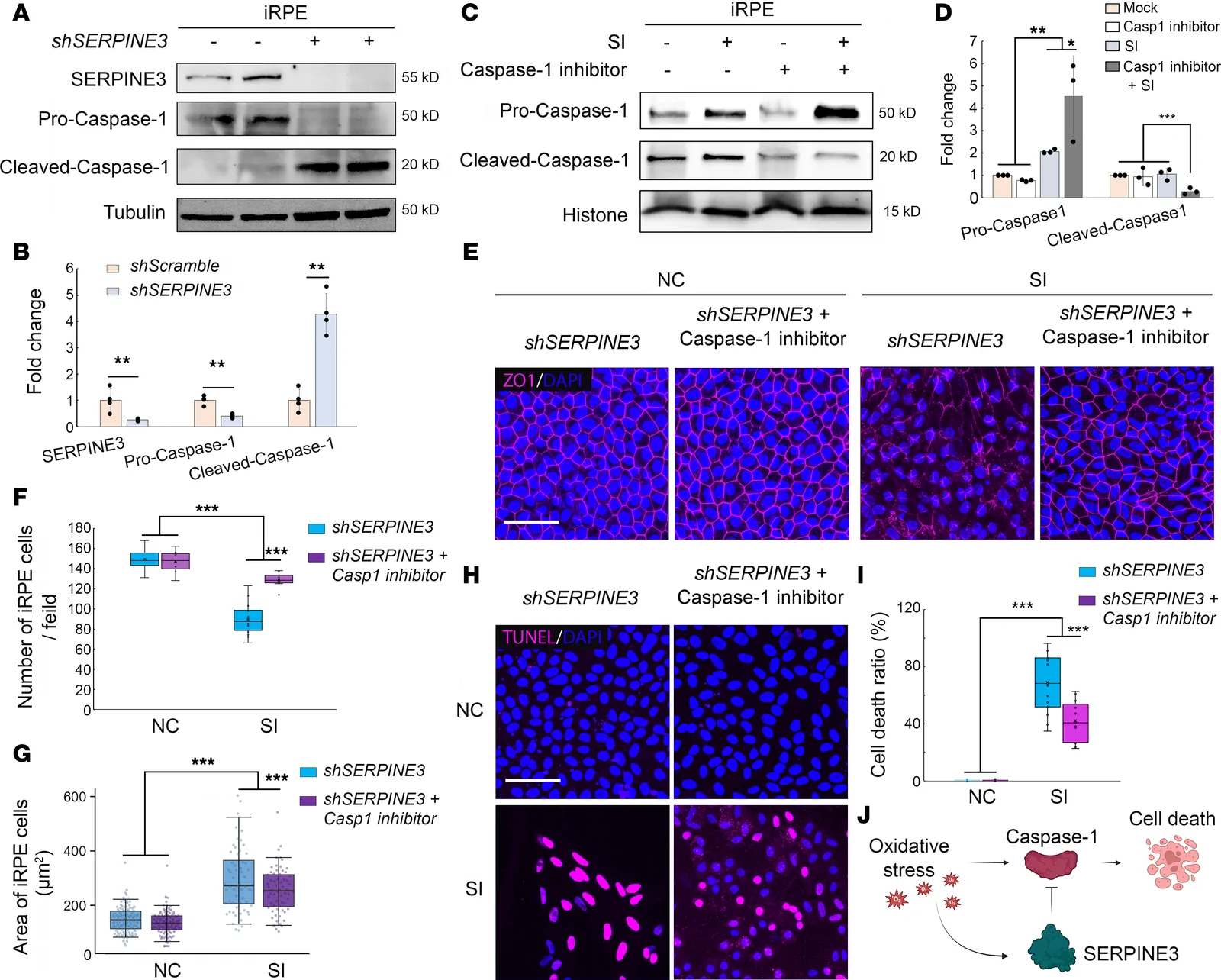
Caspase-1 inhibitor prevents SI-induced cell death in SERPINE3-deficient iRPE. (**A** and **B**) Western blotting (**A**) and quantification (**B**) show increased cleaved caspase-1 in SERPINE3-deficient iRPE cells. (**C** and **D**) Conversely, Western blotting (**C**) and quantification (**D**) indicate that the caspase-1 inhibitor reduces cleaved-caspase-1 in iRPE cells. (**E**–**G**) Anti-ZO1 immunostaining (**E**) with quantification of iRPE cell number (**F**) and single-cell area (**G**) is shown under the indicated conditions. (**E** and **H**) Scale bar: 50 μm. (**H** and **I**) TUNEL staining (**H**) with quantification of TUNEL^+^ cells (**I**) is shown under the same conditions. (**J**) Schematic summarizes how SERPINE3 may prevent oxidative stress–induced RPE death. The box-and-whisker plots depict the minimum and maximum values (whiskers), the upper and lower quartiles, and the median. Data are presented as mean ± SD. Statistics were calculated by unpaired 2-tailed Student’s *t* test (**B**) or 2-way ANOVA followed by Tukey’s test (**D**, **F**, **G**, and **I**). *P < 0.05, **P < 0.01, ***P < 0.001.

To investigate whether caspase-1 mediates the function of SERPINE3 in oxidative stress–induced RPE degeneration, we treated the SERPINE3-deficient iRPE cells with caspase-1 inhibitor under SI treatment conditions. First, we confirmed that just like the mouse model, knockdown of SERPINE3 induces cleavage of caspase-1 in iRPE cells (Figure 8, A and B), confirming that SERPINE3 regulates cleavage of caspase-1. Next, we tested the effect of AC-YVAD-CMK, a known caspase-1 inhibitor, on the activity of caspase-1 in iRPE cells. The Western blot data showed that treatment of SI increased the pro–caspase-1 level but did not affect the cleaved-caspase-1 level, suggesting that SI upregulates caspase-1 (Figure 8, C and D). Interestingly, after treatment with AC-YVAD-CMK, the pro-caspase-1 level was robustly increased, while the cleaved-caspase-1 level was significantly decreased under SI treatment conditions (Figure 8, C and D), suggesting that AC-YVAD-CMK indeed blocked caspase-1 activity. Interestingly, treatment with AC-YVAD-CMK partially blocked the degenerative phenotype of SI-treated SERPINE3-deficient iRPE cells, preserved RPE tight junctions and their morphology (Figure 8, E and F), and suppressed increase in cell size (Figure 8, E and G). Consequently, treatment of AC-YVAD-CMK also reduced the cell death in SERPINE3-deficient iRPE cells under SI treatment conditions (Figure 8, H and I). Collectively, these data suggest that oxidative stress upregulates both SERPINE3 and caspase-1 but upregulated SERPINE3 could inhibit caspase-1 to prevent RPE cell death (Figure 8J).

### AAV-Serpine3 partially reduces RPE degeneration in the SI injury model.

**Figure 9:**
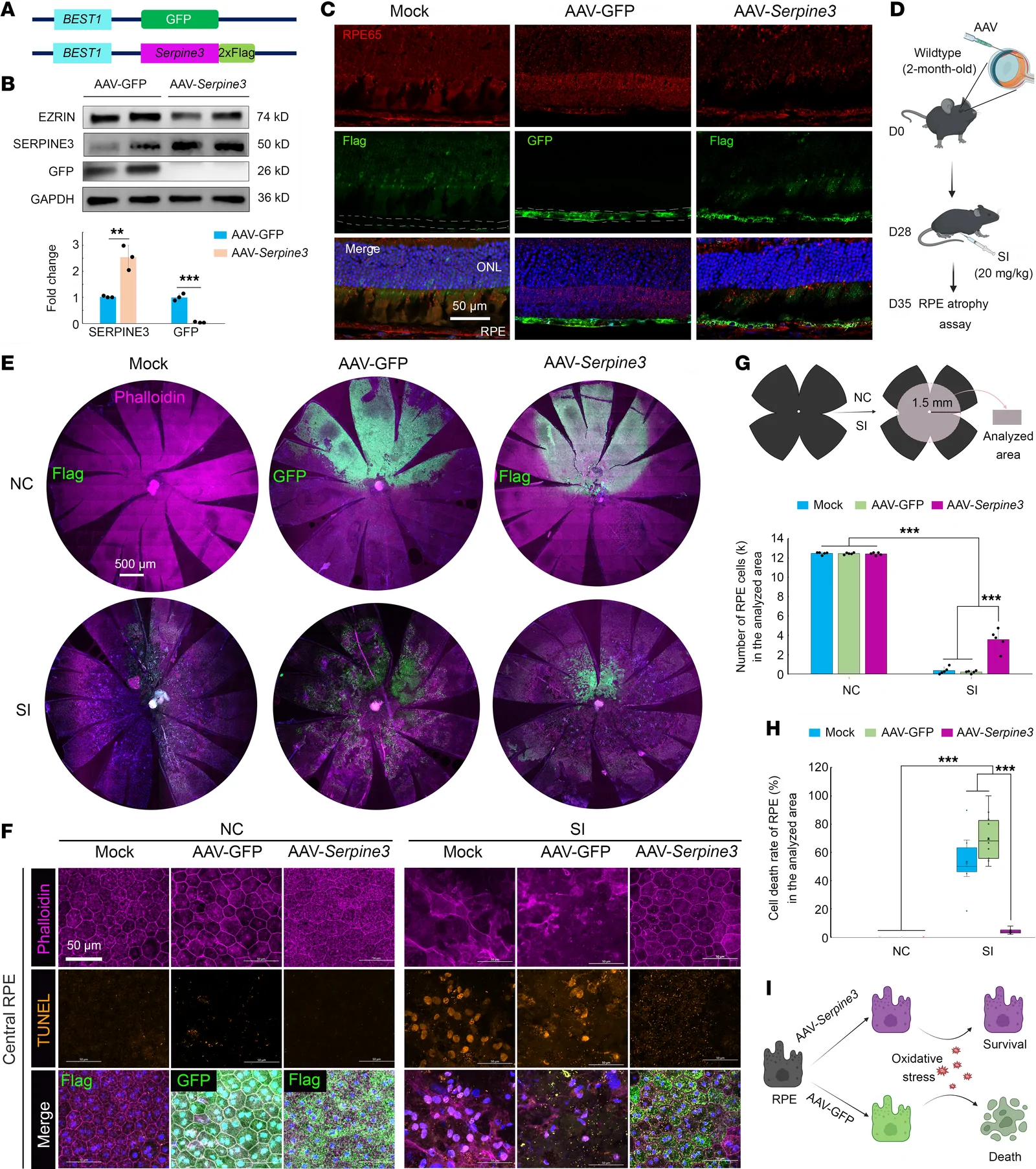
SERPINE3 overexpression partially reduces RPE degeneration after SI injury. (**A**) Schematic of AAV-GFP and AAV-Serpine3 constructs. (**B**) Western blots (upper panels) show SERPINE3 expression in the retina for 4 weeks after subretinal injection of AAV-Serpine3. The bar graph (lower panels) quantifies the relative expression of SERPINE3 based on Western blot results (*n* = 3). (**C**) Immunostaining images show the immunoreactivity of SERPINE3-Flag in 2-month-old WT retinas, 4 weeks after AAV-Serpine3. Scale bar: 50 μm. (**D**) Schematic of SI-induced RPE degeneration in mice transduced with either AAV-Serpine3 or AAV-GFP. (**E**) RPE flat-mount immunostaining of anti-GFP or anti-Flag with phalloidin staining in the WT mice under each condition (*n* = 5). Scale bar: 500 μm. (**F**) RPE flat-mount TUNEL staining with phalloidin staining in the WT mice under each condition (*n* = 5). Scale bar: 50 μm. (**G**) Quantification of RPE cell number in the central area of flat mounts from the WT mice, comparing AAV-GFP and AAV-Serpine3 under the indicated conditions (*n* = 5). (**H**) Quantification of TUNEL^+^ cells in the central RPE of WT mice under the indicated conditions. (**I**) A schematic summarizes the different SI-induced RPE degeneration between the eyes transfected by AAV-GFP and AAV-Serpine3. ONL, outer nuclear layer. Data are presented as mean ± SD. Statistics were calculated by unpaired 2-tailed Student’s *t* test (**B**) or 2-way ANOVA followed by Tukey’s test (**G** and **H**). **P < 0.01, ***P < 0.001.

Given that SERPINE3 inhibits caspase-1 activity, a key inflammasome effector that induces cell death (40), we asked whether SERPINE3 augmentation could prevent RPE degeneration in an oxidative damage model. Here, we used AAV-Serpine3 to overexpress *Serpine3* in RPE cells and used AAV-GFP as a control (Figure 9A). Expectedly, 4 weeks after subretinal delivery, GFP signal was observed in the central area of retinas transduced with AAV-GFP but not in AAV-Serpine3 transduced animals (Supplemental Figure 11A), whereas SERPINE3 was significantly increased in RPE transduced with AAV-Serpine3 and not in RPE transduced with AAV-GFP (Figure 9B). Furthermore, the RPE-specific promoter Best1 drove GFP and SERPINE3-Flag expression only in the RPE cells (Figure 9C). Neither AAV-Serpine3 nor AAV-GFP caused any significant changes to the retina anatomy (Supplemental Figure 11, B and C), suggesting that these virus vectors are safe and effective in delivering SERPINE3 to the RPE. The transduction efficiency of AAV-Serpine3 and AAV-GFP in the RPE is up to 40% and 48%, respectively (Supplemental Figure 11D).

To analyze the role of AAV-Serpine3 in RPE degeneration, we used an SI-induced RPE degeneration mouse model. Virus vector–transduced mice were subjected to SI treatment at a lower dose (20 mg/kg). After SI treatment for an additional 7 days, RPE degeneration was analyzed via RPE flat-mount immunostaining (Figure 9D). Expectedly, the central RPE cells in control mice, transduced with AAV-Serpine3 or AAV-GFP and receiving no SI treatment, displayed the typical hexagon shape. The same was true for mice transduced with AAV-Serpine3 after treatment with SI. However, the central RPE cells of non-AAV or AAV-GFP transduced mice after SI treatment lost their ability to maintain their hexagon shape (Figure 9, E and F). Consistently, in mice transduced with AAV-Serpine3 and treated with SI, the number of central RPE cells increased about 9-fold compared with control mice, representing about 30% of the central RPE cell number under normal conditions (Figure 9G). The cell death rate fell to about 4% (Figure 9, F and H). Scotopic and photopic electroretinogram (ERG) recordings revealed no significant differences in a-wave amplitudes between the SI-treated AAV-Serpine3 and AAV-GFP groups under any tested condition (Supplemental Figure 12, A and B). However, compared with the AAV-GFP group, the AAV-Serpine3 group exhibited a modest increase in scotopic b-wave amplitude at 2 stimulus intensities (Supplemental Figure 12C) and in photopic b-wave amplitude at 1 stimulus intensity (Supplemental Figure 12, D and E). These b-wave amplitudes remained significantly lower than those of uninjured controls (Supplemental Figure 12C). Overall, Serpine3 overexpression may protect RPE cells against oxidative damage and only marginally preserve retinal function in the SI injury model (Figure 9I).

## Discussion

This study uncovers that SERPINE3 is highly expressed in a minor peripheral RPE subpopulation. These S3^+^-RPE cells mainly reside in the dorsal periphery of the eye, can evade oxidative stress–induced cell death, and exhibit distinct age-associated survival, underscoring the role of RPE subpopulations in differentially regulating retinal and ocular physiology. Furthermore, this study uncovers an endogenous protective factor, Serpine3, that inhibits caspase-1 to protect RPE from oxidative damage, providing a potential therapeutic target for the treatment of macular atrophy–associated diseases.

Exploring RPE heterogeneity is critical to our better understanding of RPE biological function under different physiological and pathophysiological conditions. Omics analysis is crucial for discovering molecular differences between RPE subpopulations. Bulk RNA-seq, scRNA-seq, and ATAC-seq enabled the discovery of region-specific changes in gene expression at the transcript and chromatin levels and helped distinguish macular and peripheral RPE cells (29–32). These gene expression profile changes have been associated with the different functions of macular and peripheral RPE cells, including metabolism, visual cycle, and oxidative stress (13, 31, 45). However, in vivo, it is challenging to distinguish the subpopulations beyond the location and even more difficult to characterize their biological features. The data from our RNA-seq analysis and GFP-knockin mouse model showed that the enrichment of SERPINE3 expression in a peripheral RPE subpopulation is conserved among humans, macaques, and mice. The heterogeneous expression of SERPINE3 was also observed in the human and mouse RPE cells by other groups using scRNA-seq (46, 47). Recently, Indrischek et al. (34) found that in zebrafish, serpine3 is highly expressed in Müller glial cells compared with the RPE (34). However, they did not analyze specific RPE regions for enrichment of serpine3 expression. Our GFP reporter gene data showed that SERPINE3-GFP is expressed in peripheral RPE cells at varying levels but is absent from the neural retina, which confirms the RPE-specific expression profile of Serpine3 in mammals. In addition, this tracing system successfully distinguishes the SERPINE3^+^ subpopulation in vivo, providing an avenue to characterize the features of the S3^+^-RPE subpopulation.

Intriguingly, our results support a specific transcriptomic profile of the S3^+^-RPE subpopulation and its contribution to RPE heterogeneity during degeneration. Heterogeneous RPE degeneration is often observed in retinopathies, like AMD, where degeneration of macular and far-peripheral RPE subpopulations is seen (7). Previous studies have reported differential oxidative stress, lipid metabolism, and autophagy between macular and peripheral RPE cells (10, 13, 25, 26). Furthermore, high oxidative stress in macular RPE cells has been suggested to contribute to their higher sensitivity (15). However, why nonmacular RPE cells are resistant to oxidative stress is still unknown. Specifically, in this study, we found that the S3^+^-RPE subpopulation displays antioxidant transcriptomic signatures, including *NFE2L2* enrichment, and possesses regenerative capacity to produce new RPE cells under oxidative stress conditions. In addition, the marker gene SERPINE3 is also upregulated by acute oxidative stress and provides resistance to oxidative stress damage to RPE cells. Furthermore, SERPINE3 upregulation in the peripheral RPE of aged mice is intriguing and may suggest a response to the cumulative oxidative stress with aging. Consistently, deletion of SERPINE3 leads to a regional RPE degeneration in response to oxidative damage and results in abnormal accumulation of microglial cells in the subretinal space, suggesting increased inflammation in the subretinal environment (38, 48). Oxidative stress–induced inflammation is magnified by various TLRs and balanced by the counteracting mechanisms regulated by their inhibitors (49, 50). Our data suggest that SERPINE3 likely falls into the latter category and intracellularly inhibits cellular stress response. The predominant expression of SERPINE3 in peripheral RPE may thus be linked to the relatively higher sensitivity of central RPE to oxidative stress. In support of this argument, overexpression of SERPINE3 confers partial protection to the central RPE against oxidative stress, but achieves minimal functional recovery. The approximately 30% structural preservation is inadequate for restoring full function, so either additional factors or enhanced AAV transduction efficiency is required for robust protection. Overall, these results support a physiological difference between RPE subpopulations in their ability to sense and respond to oxidative stress, as evidenced by SERPINE3 upregulation. The generation and regenerative capacity of the S3^+^-RPE subpopulation should be further analyzed by a lineage-tracing system.

Among the many mechanisms possibly responsible for retinal degeneration, chronic inflammation is a significant factor in the pathogenesis of retinal degeneration disease (10), and the inflammatory protease caspase-1 is a crucial component of the inflammasomes (40). While the inflammasome is assembling, the bipartite adaptor protein ASC (apoptosis-associated speck-like) directly binds to and enables the proximity-induced activation of caspase-1. Upon activation, caspase-1 triggers the maturation and secretion of potent proinflammatory mediators (IL-1β and IL-18) and induces pyroptosis (51). In RPE cells, caspase-1 has been reported to mediate the inflammation caused by the accumulation of Alu, thus inducing RPE cell death (41). Interestingly, there is an intrinsic negative regulation of caspase-1 activity during inflammasome processing to deactivate caspase-1 (52). Recently, a negative feedback loop of caspase-1 activity, which occurs in a gasdermin D–dependent manner, has been uncovered (53). Furthermore, SOD1 has also been reported to negatively regulate caspase-1 activity by posttranslational glutathionylation (54). The negative feedback is critical to balance the inflammation and in turn maintain tissue homeostasis. Our study identified a negative regulation of caspase-1 activity, which occurs in a SERPINE3-dependent manner. SERPINE3 can directly bind caspase-1 and inhibit its activation, suggesting that SERPINE3 is a vital regulator in controlling caspase-1 activity. This relationship helps deepen our understanding of how SERPINE3 inhibits inflammation during aging. Given that caspase-1 is a critical component of canonical inflammasomes and a crucial pathological factor in chronic inflammation–associated diseases (55), SERPINE3 is likely to be an effective target to intervene on the pathogenesis of these diseases.

In summary, this study identified a minor subpopulation of peripheral RPE cells defined by SERPINE3 expression, which modulates regional RPE degeneration through SERPINE3-mediated inhibition of caspase-1. These findings provide mechanistic insight into the heterogeneous responses of RPE cells under degenerative conditions and highlight the potential therapeutic relevance of SERPINE3 in macular atrophy–associated diseases. Nevertheless, in the absence of lineage-tracing experiments, it remains uncertain whether SERPINE3-expressing RPE cells represent a distinct lineage or a functionally specialized state.

## Methods

### Sex as a biological variable.

Our study employed both male and female mice, except for the *Serpine3^GFP/GFP^* mice treated with SI. To reduce the variety of SI-induced RPE degeneration, administration was limited to male *Serpine3^GFP/GFP^* and to corresponding male controls, *Serpine3^GFP/+^*, and WT mice.

### Mice.

All procedures on mice with the C57BL6/J genetic background were conducted at the National Eye Institute (NEI) animal facility and were approved by the Ethical Committee (protocol NEI-680). To generate the *Serpine3^GFP^*-knockin mouse, a GFP reporter with bGH sequence was inserted immediately downstream of the start codon in exon 2 of the Serpine3 gene in mice by the CRISPR/Cas9 system. To confirm the homologous recombination, 2 pairs of primers were used to amplify the 5′ terminal (Serpine-LF1 and Serpine-LR1) and 3′ terminal (Serpine-RF1 and Serpine-RR1) arms of Serpine3-GFP. All mice were genotyped at weaning by a commercial vendor (Transnetyx). A pair of primers (GFP-F1 and GFP-R1) was used for genotyping the Serpine3-GFP allele, while another pair (WT-F2 and WT-R2) of primers was used for genotyping the Serpine3-WT allele.

For SI injection, SI (NaIO_3_, Sigma-Aldrich, S4007) was freshly dissolved in sterile saline and injected into the anesthetized mice through the tail vein (20 mg/kg) as previously reported (56). SI was administered to both male and female mice, except for the *Serpine3^GFP/GFP^* group. For aging analyses, *Serpine3^GFP/GFP^* mice at 16, 18, and 22 months of age were included.

AAV vectors were injected subretinally as previously described (57). Each animal received 1 μL of AAV vector at a dose of approximately 5 × 10^8^ genome copies per eye. AAV vectors were packaged and purified using 2 rounds of CsCl density gradient centrifugation at the NEI Gene Therapy Core.

For calculating the embryonic day, noon on the day of vaginal plug presence was defined as E0.5 for timed pregnancies.

### RNAscope.

The Serpine3 probe was purchased from Advanced Cell Diagnostics. The experiments were performed according to the standard protocol provided by the company. Briefly, eyeballs were dissected to remove the anterior segment and lens in 4% PFA and fixed in 4% PFA overnight at 4°C. An 8 μm paraffin section was deparaffinized at 60°C for 30 minutes. The retinal section was incubated by *Serpine3* probe at 40°C for 2 hours and then reacted with the R1 solution conjugated with Alexa Fluor 555 fluorescence. Next, the section was subjected to immunostaining.

### RNA-seq analysis.

For bulk RNA-seq analysis, the raw data from the human RPE samples (plae.nei.nih.gov, SRP080886 and SRP098761) and macaque samples (accession number GSE194285, Gene Expression Omnibus [GEO] database) were reanalyzed. For the aged mice, the RPE/choroid tissues were obtained from 16-month-old *Serpine3^GFP/+^* and *Serpine3^GFP/GFP^* mice (accession number GSE310804, GEO database). The total RNA was extracted using the NucleoSpin RNA kit (Macherey-Nagel), and purified RNA samples were used to generate RNA libraries for NovaSeq X Plus (PE150 sequence) (Illumina). For the heatmap visualizations, we used length-scaled transcripts per million quantification from txImport scaled by library size with the edgeR (Robinson et al., 2010) (58, 59) “calcNormFactors” function. The heatmaps were made with the R package ComplexHeatmap (Gu et al., 2016).

For smart RNA-seq analysis, single RPE cells were isolated from 2-month-old *Serpine3^GFP/+^* mice and separated into 3 groups, including peripheral GFP^+^ cells, peripheral GFP^–^ cells, and central GFP^–^ cells. The smart RNA-seq analysis was performed as previously reported. Briefly, the anterior segment tissues and lenses were removed from the eye, and then the eye cups were incubated in hyaluronidase solution (1 mg/mL) at 37°C, 5% CO_2_ for 45 minutes. Afterward, the eyecups were transferred into cold HBSS(–) and divided into peripheral and central parts at the middle line. Then, each part was transferred into a dish containing fresh HBSS(–) and put on ice for 20 minutes. The neural retinas were removed gently with forceps, and the eyecups were dissociated by repeated absorption using 100 μL peptide to generate RPE sheets. The RPE sheets were collected by 200 μL peptide under a microscope and then digested by 0.05% trypsin at 37°C for 5 minutes to generate single RPE cells. The GFP^+^-RPE cells were picked up under the fluorescence microscope using a micromanipulator (Eppendorf TransferMan 4r) and transferred into 8 μL lysis buffer (Clontech SMARTer Ultra Low Input RNA Kit for Sequencing-v3). For each sample, 6–12 RPE cells were pooled. Then, the samples were subjected to RNA extraction, double-stranded cDNA was generated, and the library was constructed (Illumina HiSeq 2500; 50 bp read length, single-end mode). All kits were used as instructed by the manufacturers. The data are available in the GEO database (accession number GSE310812).

For the scRNA-seq, iRPE cells at day 40 or 60 were dissociated and passed through cell screen cuvettes to isolate mostly healthy single cells (accession number GSE310809, GEO database). The single iRPE cells were prepared with the 10x Chromium Single Cell 5′ Library & Gel Bead Kit (PN-1000014). Sample libraries were finalized and sequenced on one HiSeq X lane (150 bp PE; Macrogen) for each. Standard Cell Ranger protocol detected sample chemistry and produced “possorted” BAM files.

### Protease screen assay.

For the protease screen, members of the chymotrypsin serine protease family and cysteine protease family were included, and the protease screen was performed by Reaction Biology. Briefly, the recombinant SERPINE3 protein (CUSABIO Technology, catalog CSB-MP021083HU) was tested in 10-dose IC_50_ singlet with a 2-fold serial dilution starting at 0.1 μM against 75 proteases, and the control agonists were tested in a 10-dose IC_50_ with a 3-fold serial dilution starting at 10 μM. The protease activities were monitored as a time-course measurement of the increase in fluorescence signal from fluorescently labeled peptide substrate, and the initial linear portion of slope (signal/min) was analyzed.

### Protein structure prediction.

The protein structure of SERPINE3 was predicted using AlphaFold2 with publicly available sequences (accession number NP_001094790.1). AlphaFold2 was run with the following parameters: --model_preset=monomer_casp14 and --max_template_date=2020-05-14. The predicted structure shows the evolutionarily conserved topology of serpins, consisting of 3 β-sheets and 9 α-helices (44). The predicted protein structure of SERPINE3 was aligned with the SERPINE1 protein structure (PDB: 1DVM, chain A). The RMSD between the SERPINE1 protein structure and the predicted SERPINE3 structure is 1.740 when calculated using the “super” command in PyMOL. An RMSD value of ≤ 2 is considered fairly good and indicates a close match between 2 protein structures (60).

A detailed description of other experimental methods is provided in the supplemental materials.

### Statistics.

Data were from at least 3 replicates for each experimental condition and are presented as mean ± SD. 2-way ANOVA with Tukey’s test was used to determine the significance between population means. 2-tailed Student’s *t* test was used to determine the significance of differences when only 2 groups were compared. DESeq2 was used to determine differential expression of genes for the RNA-seq data, and a 2-tailed paired *t* test was used to determine the significance of difference for the ERG data from the WT mice transduced by AAV-GFP or AAV-Serpine3. *P* < 0.05 was considered statistically significant.

### Study approval.

All procedures were conducted at the NEI animal facility and were approved by the Ethical Committee (protocol NEI-680).

### Data availability.

The RNA-seq datasets generated in this study have been deposited in the NCBI Gene Expression Omnibus (GEO) database under accession numbers GSE310804, GSE310809, and GSE310812, corresponding to bulk RNA-seq of aged *Serpine3^GFP/GFP^* RPE, single-cell RNA-seq of iRPE cells, and SMART RNA-seq of Serpine3-GFP+ cells from 2-month-old *Serpine3^GFP/+^* mice, respectively. The data are publicly accessible at https://www.ncbi.nlm.nih.gov/geo/ Values for all data points in graphs are reported in the Supportng Data Values file.

## Author contributions

HL and RBH provided the conceptual framework for this project and oversaw the execution of the experiments in their entirety. HL, VPK, and WL performed the smart RNA-seq experiments. WY, HL, and BG mapped the protein domains of SERPINE3. TDF performed the scRNA-seq analysis, and DMM and KDK analyzed the RNA-seq data. DO, DR, RS, and KB performed the RPE reshape experiments and analyzed the data. TL and HL performed the AAV subretinal injection. LD, TJS, and CZ generated and maintained the Serpine3-GFP mouse line. HL and AMR analyzed the retinal degeneration data. RBH and HL acquired the funding. HL wrote the original draft. RBH, KB, BPB, TL, WW, BG, and AMR edited the draft.

## Conflict of interest

The authors have declared that no conflict of interest exists.

## Funding support

This research was supported in part by the Intramural Research Program of the NIH and is subject to the NIH Public Access Policy. Through acceptance of this federal funding, the NIH has been given a right to make the work publicly available in PubMed Central.

- NIH Intramural Research Program.
- National Natural Science Foundation of China (82271097).

## Supplementary Material

Supplemental data

Unedited blot and gel images

Supporting data values

## Version 1 07/07/2026

In-Press Preview

## Version 2 09/01/2026

Electronic publication
