# Supplementary material for: A peripheral subpopulation of retinal pigment epithelium resists oxidative damage through SERPINE3-mediated caspase-1 inhibition
Source: J Clin Invest. 2026 Jul 7;136(17):e191272. doi: 10.1172/JCI191272 (PMC13528924; doi:10.1172/JCI191272)
Supplement: Supplementary file 1 [file jci-136-191272-s136.pdf]

Supplemental material and methods

Cell culture

For the HEK293T cell line, cells were cultured with DMEM base medium contained 10% FBS. The lipofectamine transfection kit was used for gene delivery in the HEK293T cells according to the protocol. Briefly, the 2 μg of plasmid DNA was mixed with 2 μl of lipofectamine solution and then added into a well of 6-well-plate with about 70% confluent of HEK293T cells. After 2 days of transfection, cells were lysed for protein extraction or fixed for the immunostainings.

The procedure of iPSC induced RPE (iRPE) cells was performed as previously report (58). Briefly, the wildtype iPSC line was seeded in 6-well-plate (200k/well) with E8 completed medium at day1. From day 2 to day 10, the cells were cultured in RDM media, and the media was changed every day. From day 11 to day 20, RMNA media was used and changed every day. Day 21–25, the cells were cultured in 5% RPE media to get the RPE precursors. At Day 25, the PRE precursors were seeded at 24–30 million in T75 flask in 20 mL of RPE plating media. At day 40, immature RPE cells were purified by the MACS using anti-CD24 (1:500) and anti-CD56 (1:500). The purified immature RPE cells were seeded on transwells and continuously cultured in 5% RPE medium. At 10 days post- seeding, change the 5% RPE maturation media to 5% RPE media + PGE2 and continue for 5 weeks with PGE2-supplemented media. For knocking down SERPINE3, the iRPE cells were cultured in the 5% RPE medium containing the lenti-shSERPINE3 virus ($1x10^{10}$ genome copies / well) for 12 hours, then the medium was switched to the 5% RPE medium without virus. In addition, 10 mM of sodium iodate was used to induce iRPE degeneration as shown in Figure 4I, and the 2 nM of AC-YVAD-CMK was used to inhibit Caspase-1 activity.

EdU and TUNEL assay

The Edu (Thermofish Scientific, A10044) was dissolved in 1xPBS and intraperitoneally injected into the mice (30 mg/kg). After 4 hours of injection, the eyeballs were dissected in 1xPBS to remove the anterior segment tissues, lenses and neural retinas, and the eyecups were then fixed in 4% PFA at 4°C for 2 hours. The Edu staining was performed according to the kit protocol (Click-iT™ EdU Cell Proliferation Kit for Imaging, Alexa Fluor™ 594 dye, C10339,

Thermofish Scientific). For the TUNEL assay, the eyecups were fixed in 4% PFA 4°C overnight and then subjected to TUNEL examination. The TUNEL staining was performed according to the kit protocol (Click-iT™ Plus TUNEL Assay Kits for In Situ Apoptosis Detection, C10618, ThermoFisher).

Immunostaining

Mouse eyeballs were marked at noon and dissected in cold 1xPBS for RPE flat-mount immunostaining. The anterior segment tissues, lenses and neural retinas were removed, and the eyecups were then cut radially to make RPE flat. The dissected eyecups were fixed in 4% PFA at 4°C overnight and then subjected to immunostaining. When mounting samples, the RPE side was made sure to be apical. For retinal sectional immunostaining, eyeballs were dissected in 4% PFA, and the anterior segment tissues and lenses were removed. The eyecups were fixed again in 4% PFA at room temperature for 2 hours followed by dehydration with 20% sucrose at 4°C and being embedded in OCT compound. Then 14 μm of frozen sections were used for immunostaining. Human eyecups were obtained from the Eye Bank of Wenzhou Medical University & Wenzhou Eye Bank and fixed in 4% PFA for 12 hours at 4°C. 5 μm paraffin sections were used for immunostaining. For cell immunostaining, cells were fixed in 4% PFA at RT for 1 hour and then subjected to immunostaining. For human retinal immunostaining, the paraffin section was deparaffined at room temperature and then treated by TrueBlack to remove the autofluorescence of RPE. The primary and secondary antibodies are listed in Table 1. The images were taken by the microscope Zeiss Air scanner 800.

Caspase-1 inflammasome activity assay

The activity of Caspase1 was measured with the Caspase-Glo® 1 Inflammasome Assay kit (Promega, G9951) according to the protocol. Briefly, 25μl of the recombinant Caspase1 (Sigma, C5482) was mixed with the same volume of recombinant SERPINE3 solution (Cedarlane, 116484) and incubated for 5min at room temperature. Then, the 50μl of reconstituted Z-WEHD Substrate was added into the mixed solution. Luminescence was recorded after 30 minutes.

Co-Immunoprecipitation

The protein binding ability was analyzed by the co-immunoprecipitation kit (Thermo Scientific, 26149), and the co-IP was performed according to the standard protocol. Briefly, the IP Lysis buffer contained protease inhibitors cocktails (Sigma, P1860) was applicated to extract the protein of HEK293T cells transfected by the *SERPINE3-Myc* (Origene, RC225688) and *CASPASE1-HA* plasmids in which the Myc tag of Human CASP1 Clone (Origene, RC218364) was replaced with HA tag). In addition, 100μg of primary antibody was coupled with 100μl of the Resin in the reaction hood at room temperature for 1.5 hours. The protein samples (400μl) were added into the hood with the Resin conjugated the antibody and shook overnight at 4°C. The target proteins recognized by the antibody was separated by the filter and subjected to the western blot assay.

Western blot

For protein extraction, RPE tissue or RPE cells were lysed by RAPI buffer containing cocktail proteinase inhibitor on ice for 10 min. Samples were separated by 10% SDS-PAGE. Table 1 shows information on antibodies used in western blot. Primary antibodies were incubated at 4°C overnight. The quantification of the protein bands was done using Image J software.

Supplemental figures and legends

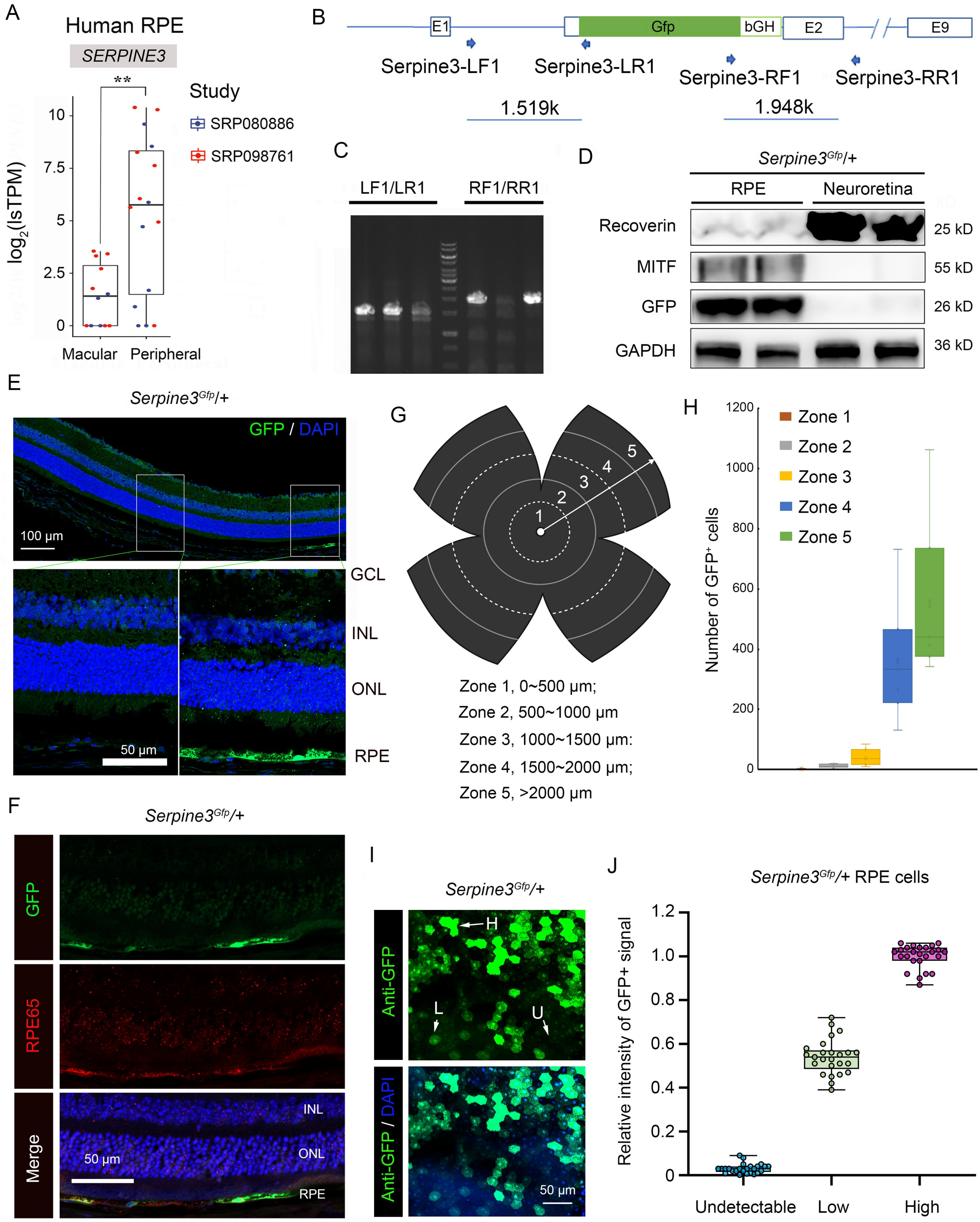

Supplemental Figure 1. Serpine3-GFP^{+}-RPE cells are concentrated at the peripheral area in *Serpine3^{Gfp}/+* mice at postnatal 2 months. (A) Dot plots of SERPINE3 expression in the human macular and peripheral RPE from the RNA-seq

data (SRP080886 and SRP098761). (B) Schematic diagram of primer design for genotyping. (C) PCR images amplifying the Serpine3-Gfp sequences from gene-edited mice. (D) Western blotting shows GFP expression in RPE and neuroretina (n=4). (E) Images of anti-GFP immunostaining in *Serpine3*^{Gfp}/+ mice at 2 months; GFP signal appears only in the RPE of *Serpine3*^{Gfp}/+ mice. (F) Sectional immunostaining of anti-GFP and anti-RPE65 in retina (n=4). (G) Schematic diagram of flat-mount RPE shows 5 analyzed zones. (H) Quantification of GFP+ cells localized at these zones (n=7). (I, J) RPE flat mount immunostaining of anti-GFP in in *Serpine3*^{Gfp}/+ mice at 2 months and quantification of GFP^{+} signal intensity in the peripheral RPE with varying levels (n=5). GCL, ganglion cell line; INL, inner nuclear layer; ONL, outer nuclear layer; RPE, retinal pigment epithelium. Data presented as mean ± SD (G). DESeq2 was used to determine the different expression of *SERPINE3* (B). **<0.01.

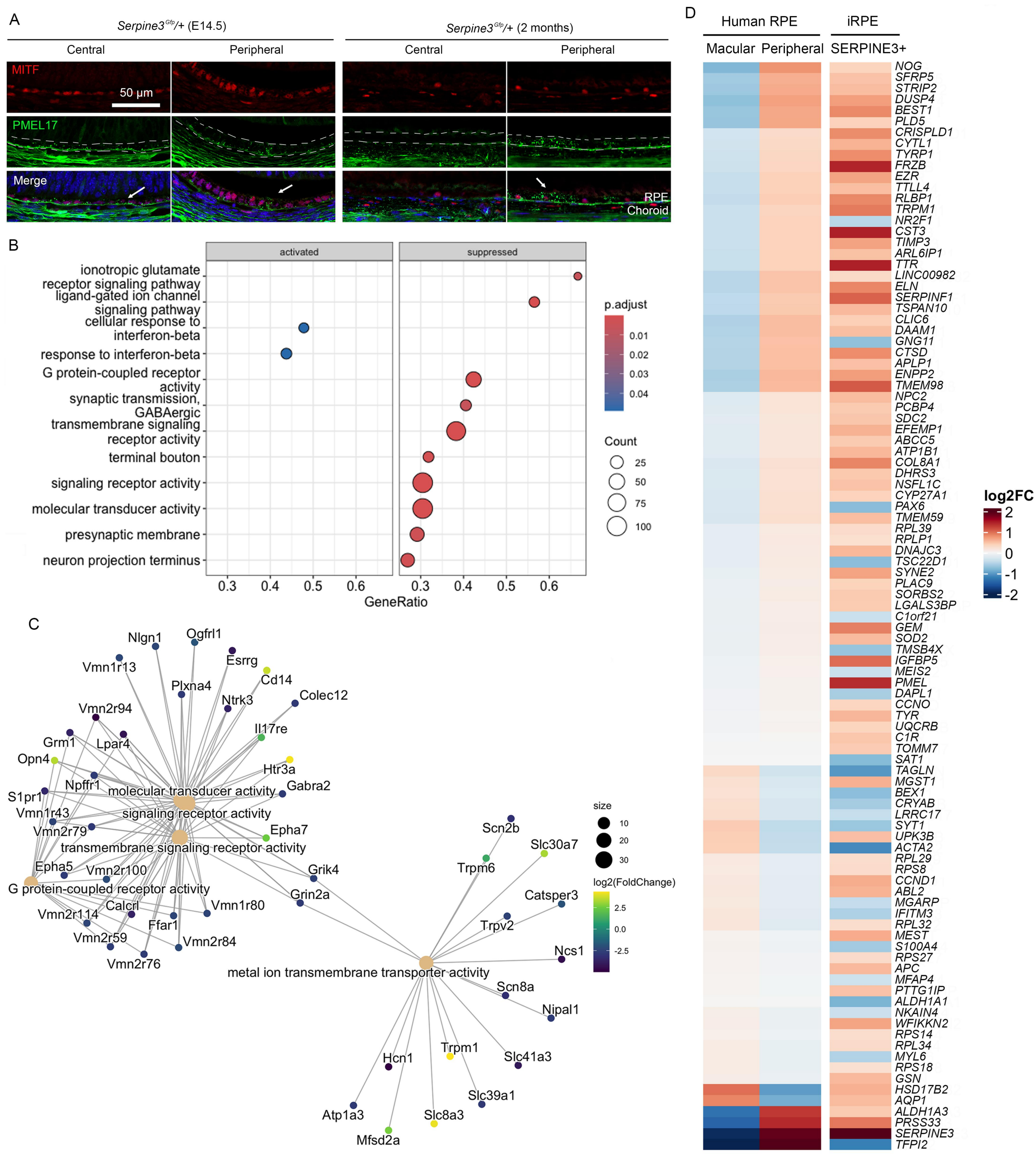

Supplemental Figure 2. Transcriptomic analysis of GFP+ RPE cells from 2-month-old *Serpine3*^{Gfp}/+ mice and SERPINE3^{+} RPE cells derived from human iPSCs. (A) Immunostaining for anti-PEML17 and anti-MITF in *Serpine3*^{Gfp}/+ mouse retinal sections at embryonic day 14.5 (left) and 2 months (right), showing marker localization in RPE cells. (B) Dot plots from Gene Set Enrichment Analysis (GSEA) presenting the top 12 signaling pathways with differential activation or inactivation in GFP^{+} RPE cells. (C) CNet plots visualize differentially expressed genes (DEGs),

grouped by pathway and colored by up- or downregulation relative to central GFP^{-}RPE control cells. (D) A heat map compares the gene expression profiles of $SERPINE3^{+}$-iRPE cells with those of human macular and peripheral RPE cells, highlighting similarities and differences.

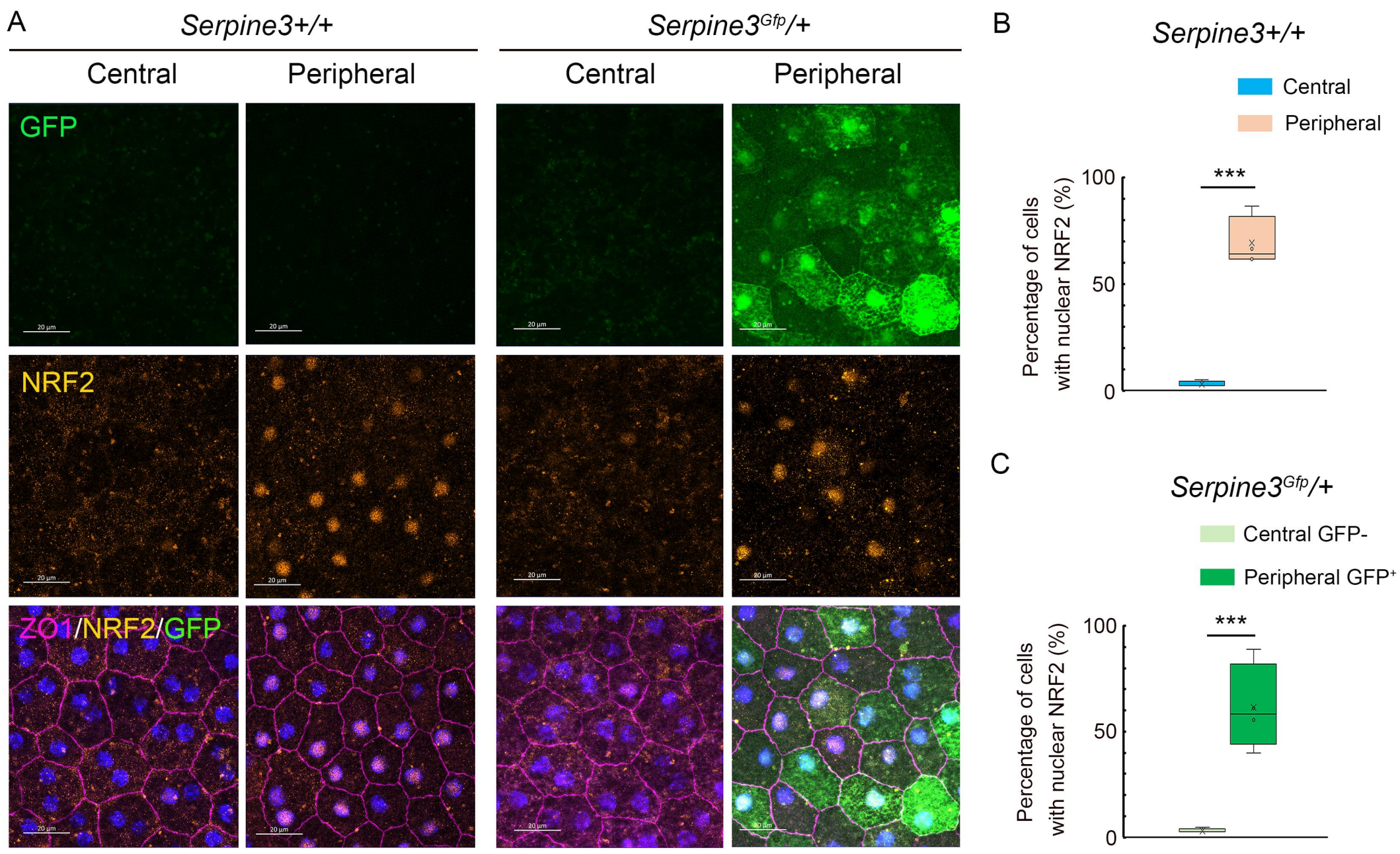

Supplemental Figure 3. Nuclear localization of NRF2 is enriched in peripheral GFP^{+} cells from 2-month-old *Serpine3^{Gfp}/+* mice. (A) RPE flat-mount immunostaining of anti-GFP, anti-NRF2 and anti-ZO1 in the *Serpine3+/+* and *Serpine3^{Gfp}/+* mice (n=4). (B, C) Quantification of the percentage of cells with nuclear NRF2 in the peripheral and central RPE cells from *Serpine3+/+* (B) and *Serpine3^{Gfp}/+* mice (C). Data indicate the mean ± SD (B, C). Statistics were calculated by unpaired 2-tailed Student's *t* test (B, C). ***P<0.001.

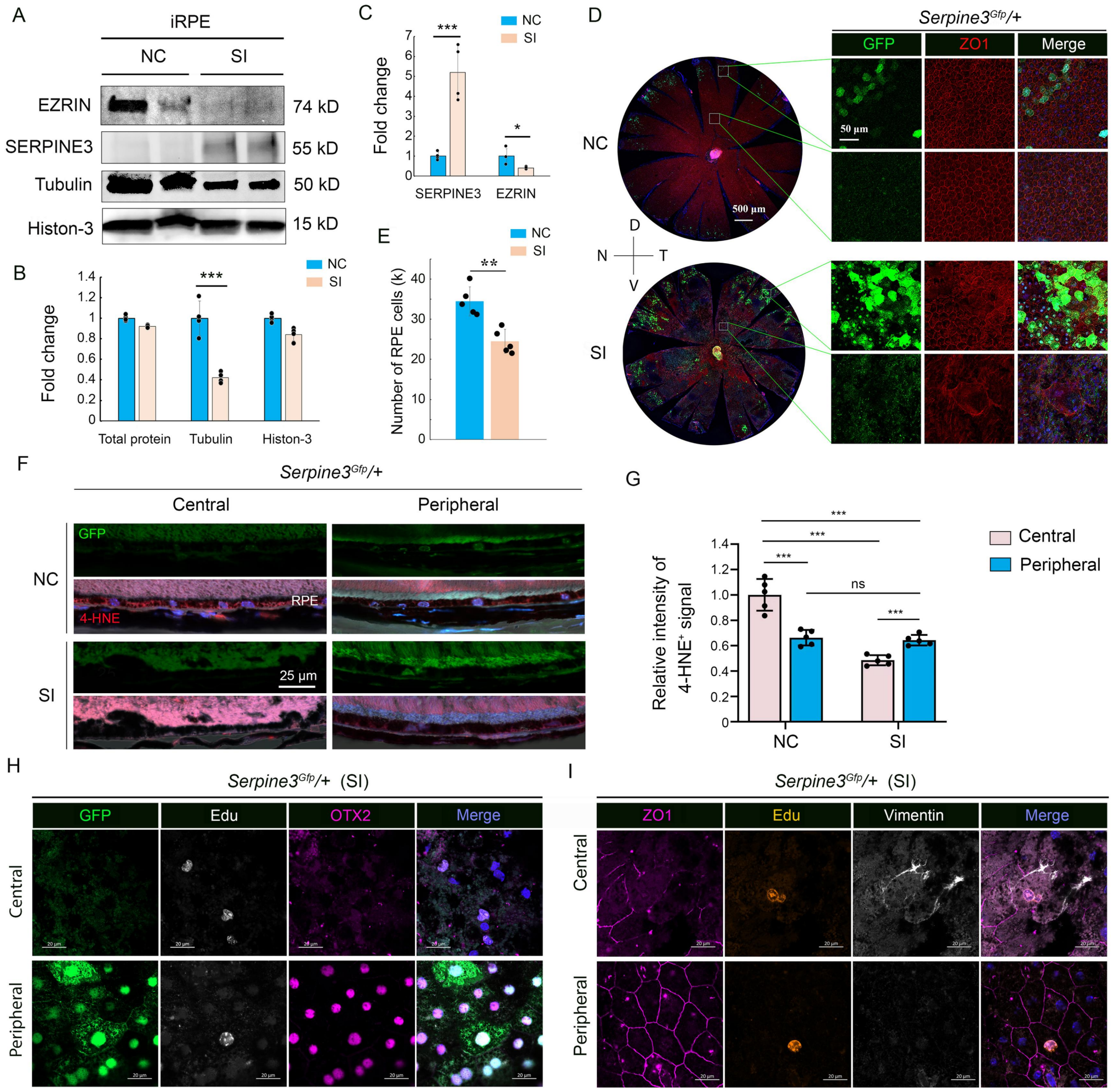

Supplemental Figure 4. GFP$^+$-RPE cells can survive and regenerate new RPE cells in *Serpine3*^{Gfp}/+ mice after sodium iodate treatment. (A-C) Western blotting and dot quantification show a significant increase in SERPINE3 and a sharp decrease in EZRIN in iRPE cells after SI treatment (n=4). (D) Immunostaining images of anti-GFP and anti-ZO1 in RPE from 2-month-old *Serpine3*^{Gfp}/+ mice with saline (upper panels) or sodium iodate (lower panels) treatment. (E) Bar graphs show a decrease in GFP+ RPE cells in *Serpine3*^{Gfp}/+ mice after SI injury (n=4). (F, G) Retinal sectional immunostaining of anti-4-HNE and anti-GFP in the *Serpine3*^{Gfp}/+ mice (F) and the quantification of 4-HNE signal intensity in the peripheral and central RPE under physiologic or SI-injured conditions (G) (n=5). (H, I) Immunostaining images of anti-GFP and anti-OTX2 (H) or anti-Vimentin (I) with Edu staining in flat-mount RPE of *Serpine3*^{Gfp}/+ mice after SI treatment (n=4). Data presented as

mean ± SD. Statistics were calculated by unpaired 2-tailed Student's *t* test (B, C and E), or 2-way ANOVA followed by Tukey's test (G). **P<0.01, ***P<0.001.

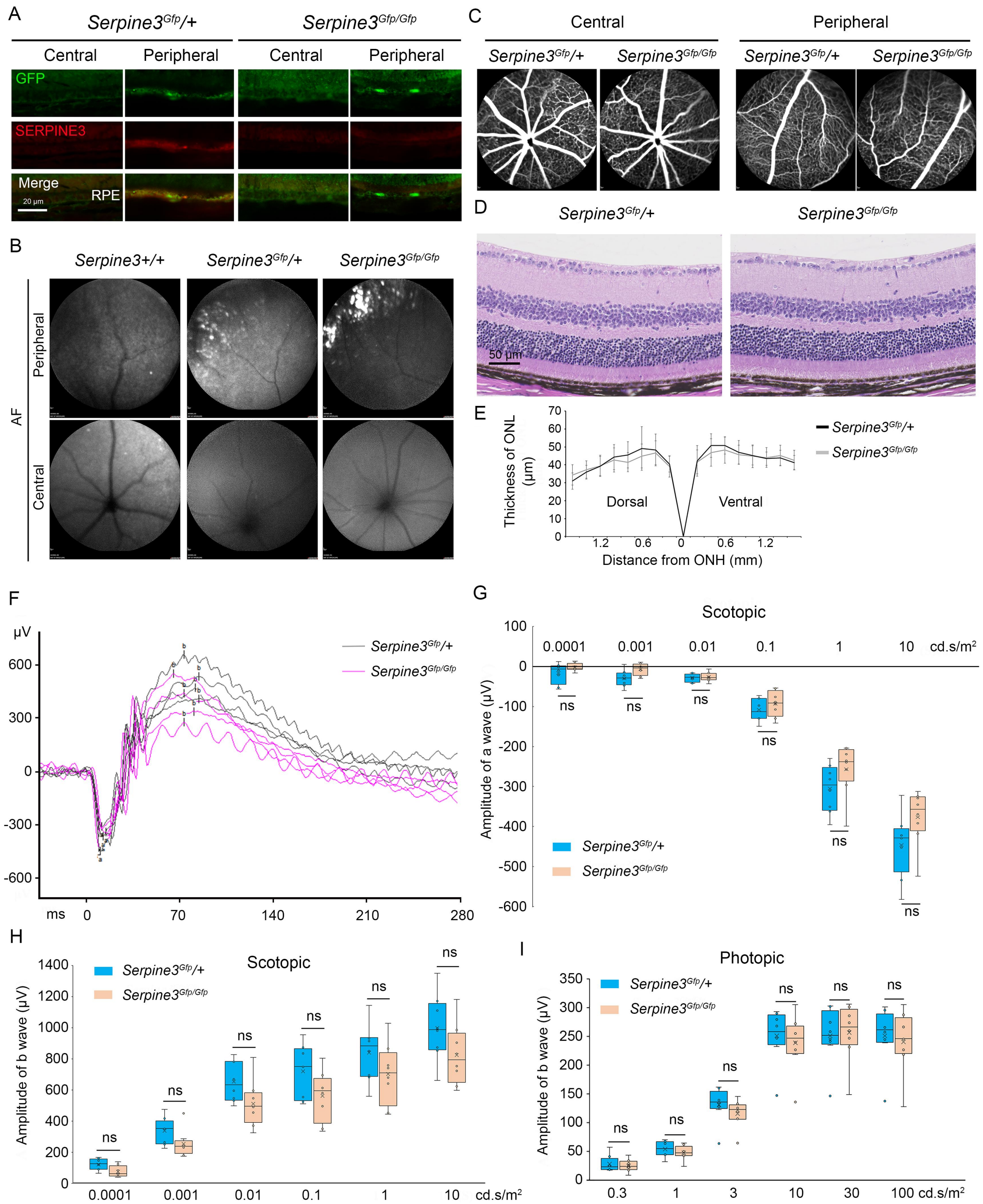

Supplemental Figure 5. Deletion of SERPINE3 does not affect the survival of GFP+-RPE cells, retinal delamination, pigmentation, or retinal function in young mice. (A) Retinal sectional immunostaining of anti-GFP and anti-SERPINE3 in the *Serpine3*^{Gfp}/+ and *Serpine3*$^{Gfp/Gfp}$ mice. (B) Autofluorescence fundus images show peripheral and central retinas from *Serpine3+/+*, *Serpine3*^{Gfp}*/+,* or *Serpine3*$^{Gfp/Gfp}$ mice at 2 months. (C) Autofluorescence fundus images of *Serpine3*^{Gfp}/+ and *Serpine3*$^{Gfp/Gfp}$ mice after 5 minutes of fluorescence sodium

injection. (D and E) Histological images of the retinas (D) and quantification of outer nuclear layer thickness (E) from *Serpine3*$^{Gfp/+}$ and *Serpine3*$^{Gfp/Gfp}$ mice at 2 months. (F) Scotopic ERG traces of *Serpine3Gfp/+* and *Serpine3*$^{Gfp/Gfp}$ mice at 2 months with flashlight stimulation (10(S)cd.s/m2). (G-I) Quantification of the amplitude of scotopic a wave (G), scotopic b wave (H) and photopic b wave (I) in *Serpine3*^{Gfp}/+ and *Serpine3*$^{Gfp/Gfp}$ mice at 2 months under the indicated condition. Data presented as mean ± SD. Statistics were calculated by unpaired 2-tailed Student's *t* test (E, G and H).

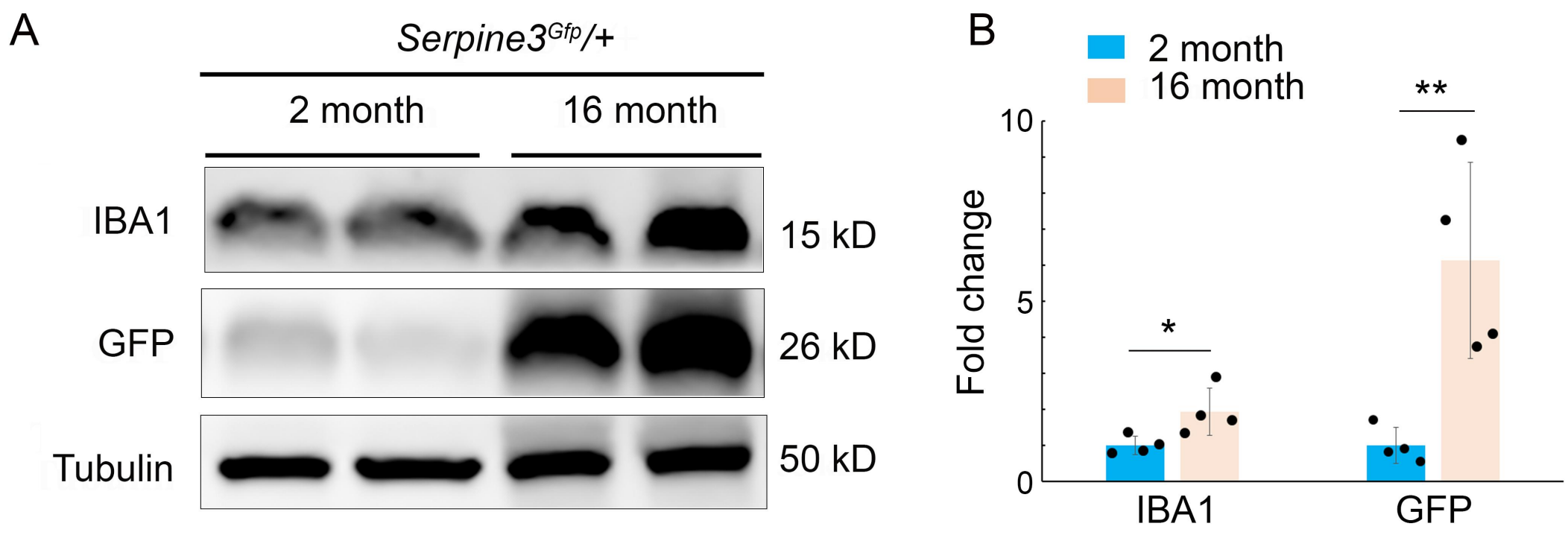

Supplemental Figure 6. GFP levels increase in aged *Serpine3^{Gfp}/+* mice. (A) Western blotting images showing EZRIN, IBA1 and GFP proteins in the RPE tissue from *Serpine3^{Gfp}/+* mice at 2 and 16-months old. (B) Quantification of dot bolts of IBA1 and GFP signals in the RPE from the *Serpine3^{Gfp}/+* mice at 2 and 16-months old (n=4). Data presented as mean ± SD. Statistics were calculated by unpaired 2-tailed Student's *t* test (B). *P<0.05, **P<0.01.

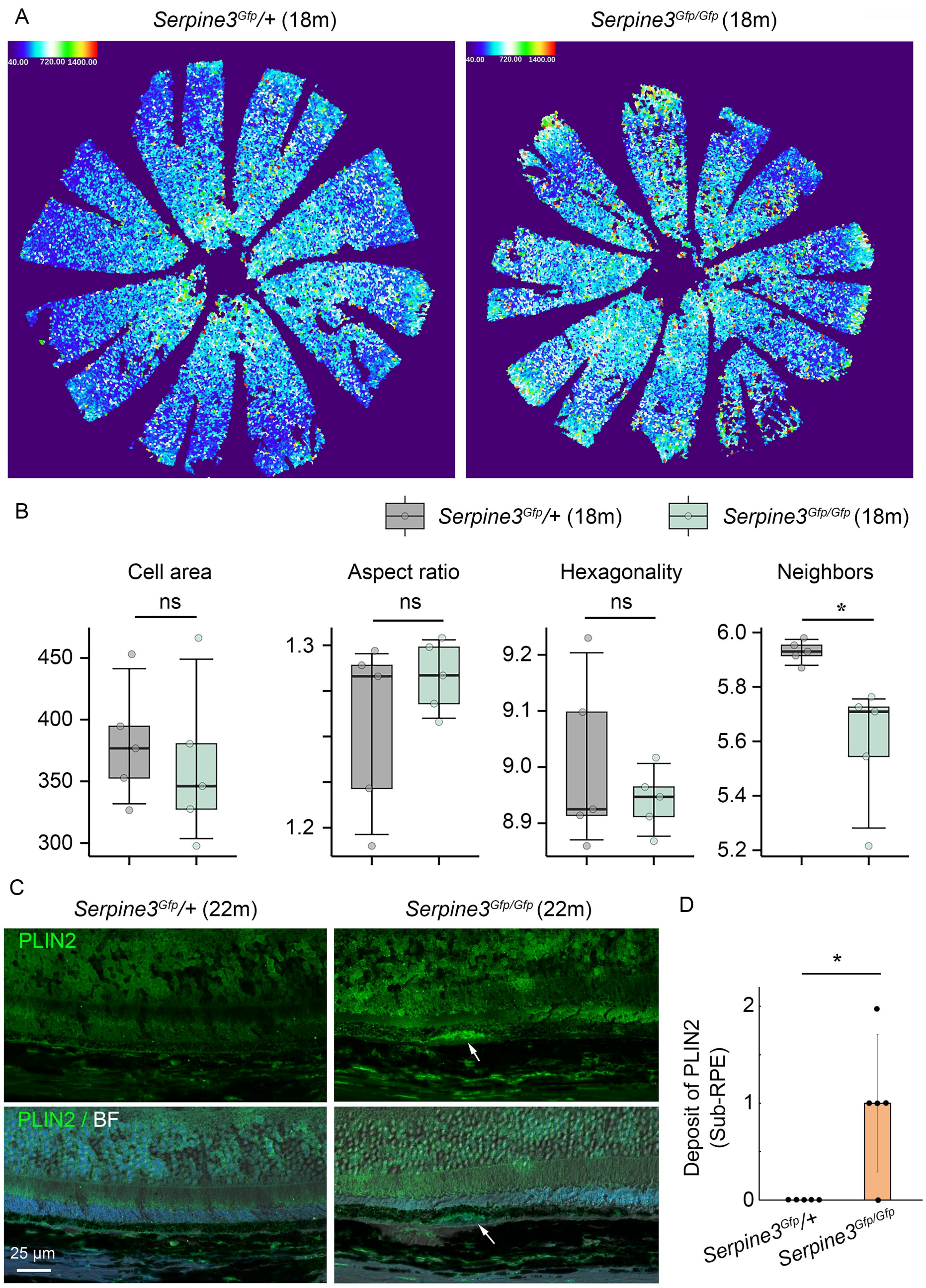

Supplemental Figure 7. Reshape analysis of the aged *Serpine3*^{Gfp}*/+* and *Serpine3*$^{\mathit{Gfp/Gfp}}$ RPE. (A) The reshaped images of RPE flat-mount immunostaining of anti-ZO1 in the *Serpine3*^{Gfp}*/+* and *Serpine3*$^{\mathit{Gfp/Gfp}}$ mice at 18 months old. (B) The statistical analysis of the RPE cells from the aged *Serpine3*^{Gfp}*/+* and *Serpine3*$^{\mathit{Gfp/Gfp}}$ mice for the cell area, aspect ratio, hexagonality and neighbors in the dorsal area. (C and D) Immunostaining for anti-PLIN2 on retinal section from 22-month-old *Serpine3*^{Gfp}*/+* and *Serpine3*$^{\mathit{Gfp/Gfp}}$ mice (C) and the quantification of PLIN2 deposits

at the sub-RPE space (D). Data presented as mean ± SD. Statistics were calculated by unpaired 2-tailed Student's *t* test (B, D). *P<0.05.

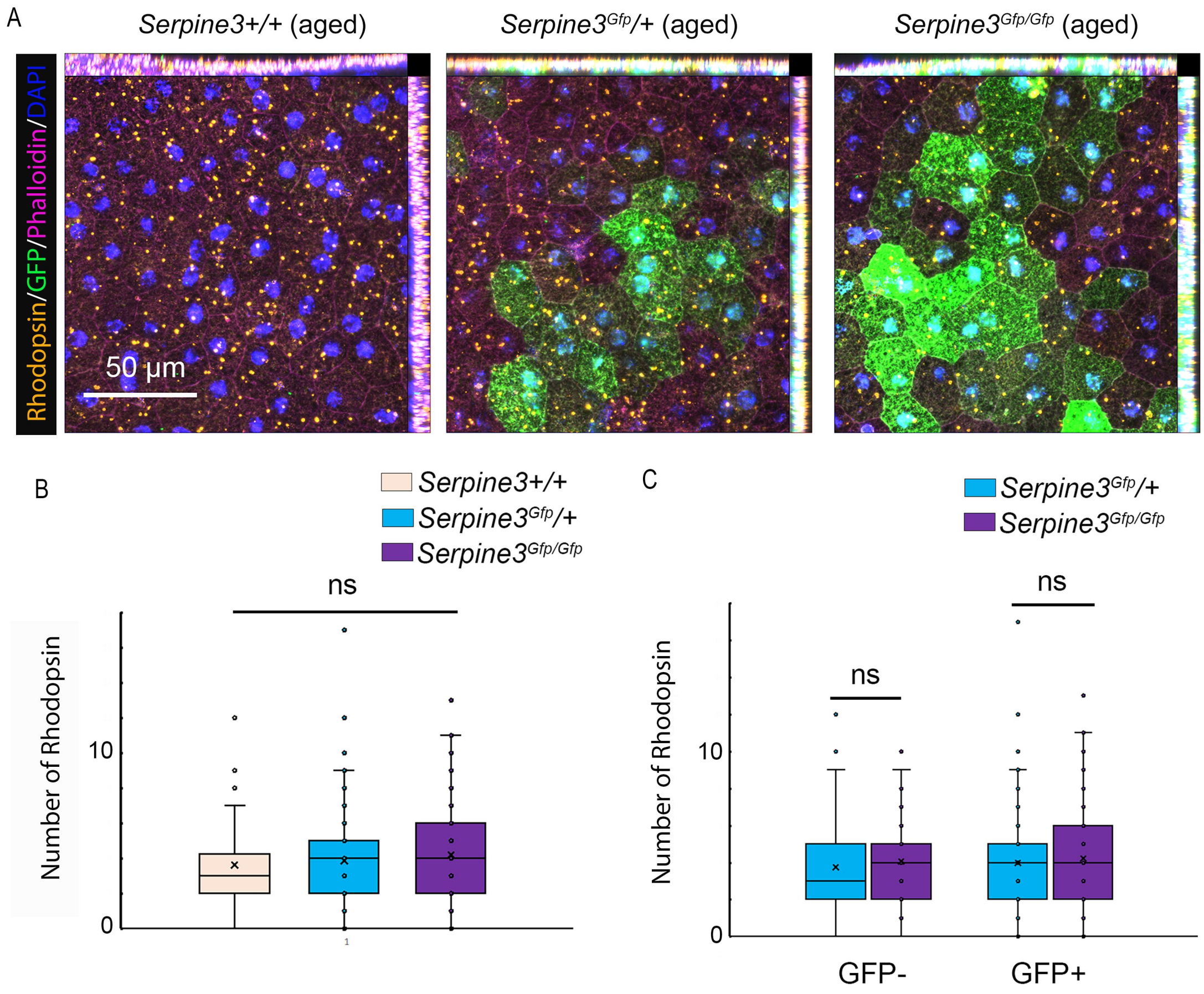

Supplemental Figure 8. Deficiency of SERPINE3 does not affect RPE phagocytosis. (A) RPE flat-mount immunostaining images of anti-GFP and anti-Rhodopsin combined with phalloidin staining in 16-month-old wildtype, *Serpine3*^{Gfp}/+ and *Serpine3*$^{Gfp/Gfp}$ mice (n=5). (B) Quantification of the number of Rhodopsin^{+} particles in each single RPE cell from the aged wildtype, *Serpine3*^{Gfp}/+ and *Serpine3*$^{Gfp/Gfp}$ mice (n=5). (C) Quantification of the number of Rhodopsin^{+} particles in each single GFP^{+} or GFP^{-} RPE cells from the aged *Serpine3*^{Gfp}/+ and *Serpine3*$^{Gfp/Gfp}$ mice (n=5). Data presented as mean ± SD. Statistics were calculated by 2-way ANOVA followed by Tukey's test (B).

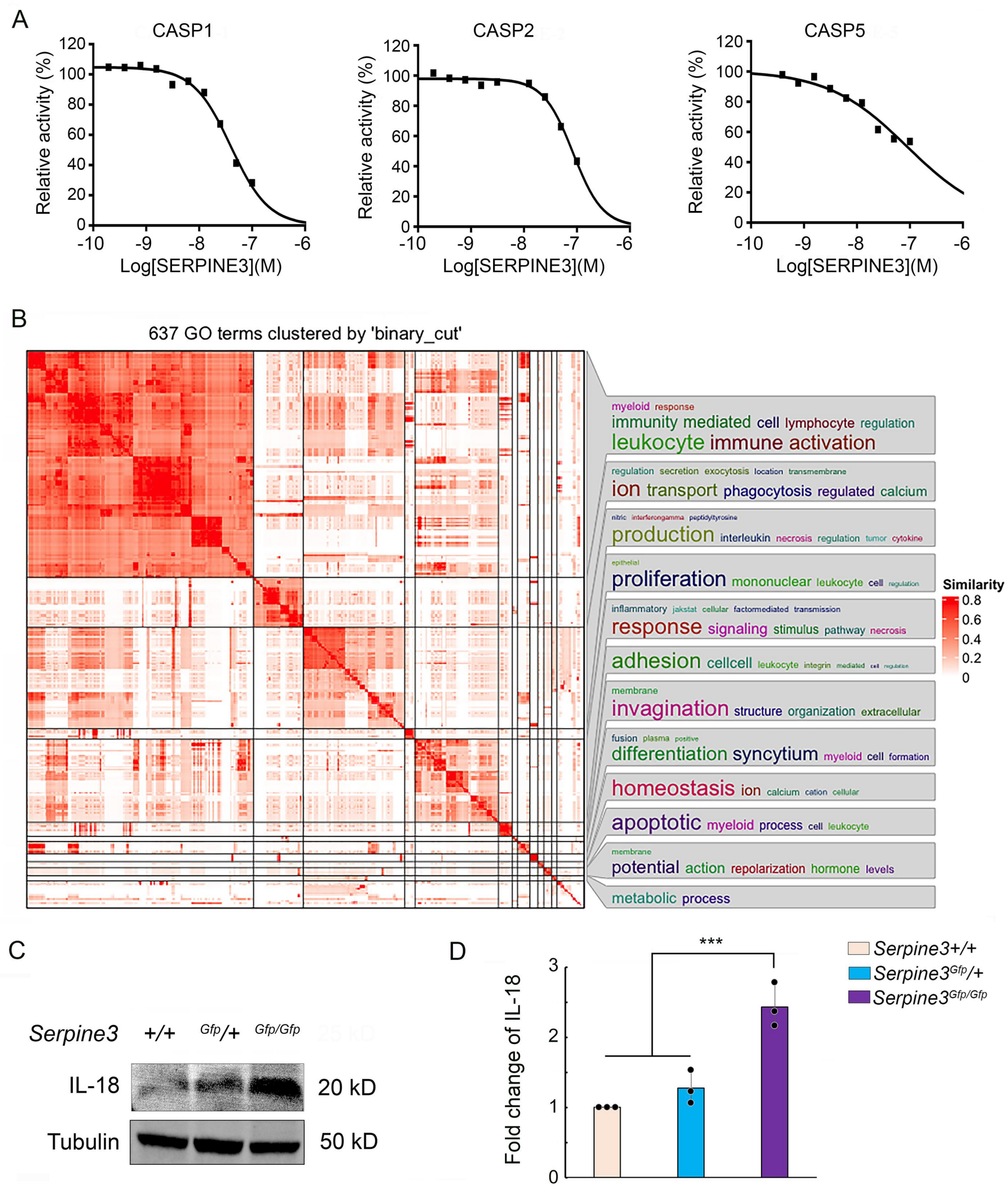

Supplemental Figure 9. SERPINE3 binds Caspase-1 and inhibits Caspase-1 activity. (A) Curve graphs show the activity of CASPASE1, CASPASE2 and CASPASE5 under the treatment of SERPINE3 at different dosages. (B) Heatmap shows the GO analysis of bulk RNA-seq from the RPE/choroidal tissue of 18-month-old *Serpine3*^{Gfp}/+ and *Serpine3*$^{Gfp/Gfp}$ mice. (C and D) Western blotting (C) and quantification of the dots (D) show a significant increase in IL-18 in aged *Serpine3*$^{Gfp/Gfp}$ mice (n=3). ***<0.001. Data presented as mean ± SD. Statistics were calculated by 2-way ANOVA followed by Tukey's test (D).

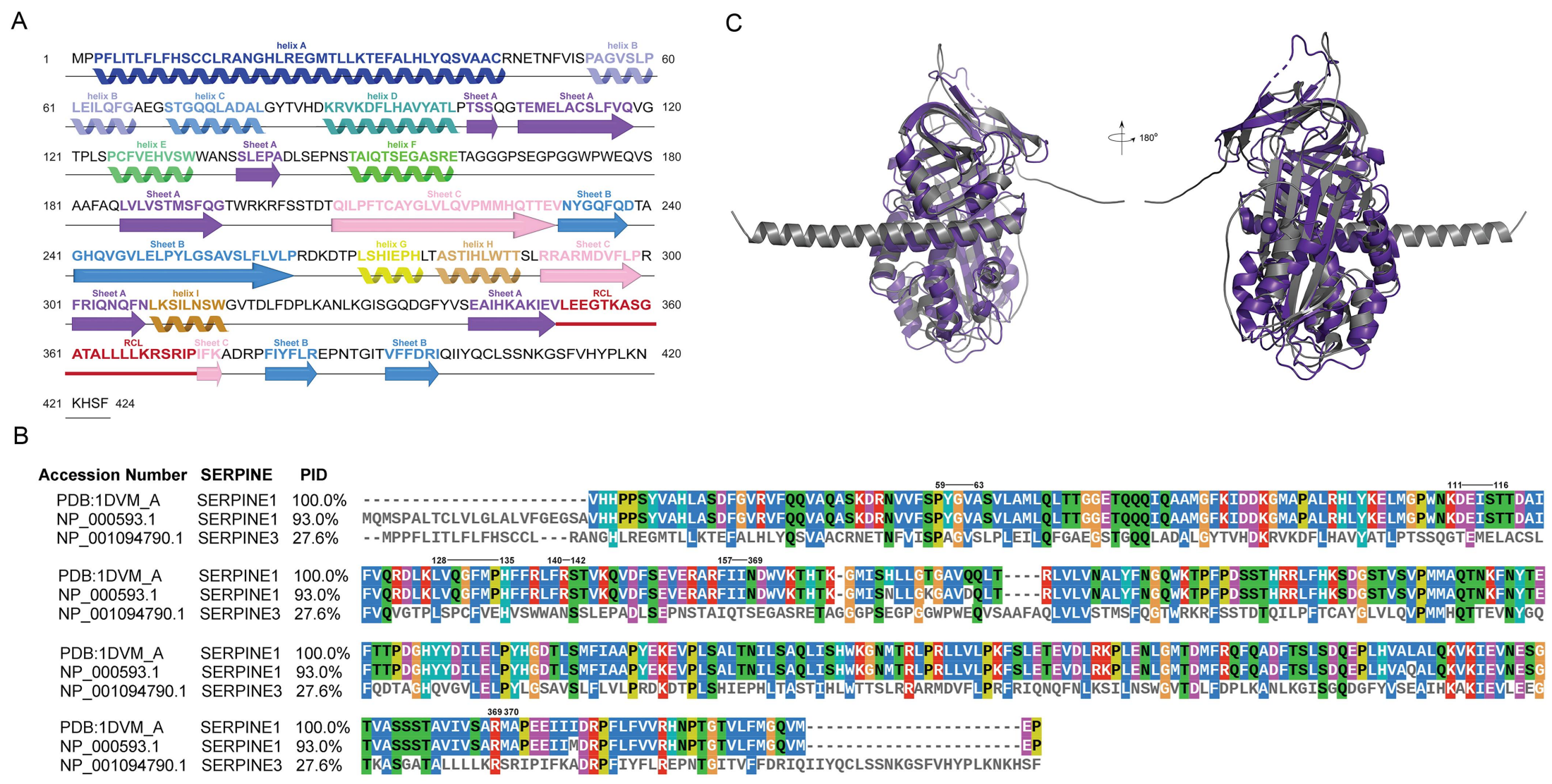

Supplemental Figure 10. Alignment analysis between SERPINE1 and SERPINE3. (A) Multiple sequence alignment among SERPINE1 and SERPINE3 sequences. PID indicates percentage identity. (B) Structure alignment between the SERPINE3 predicted protein structure (gray) and the SERPINE1 protein structure (purple).

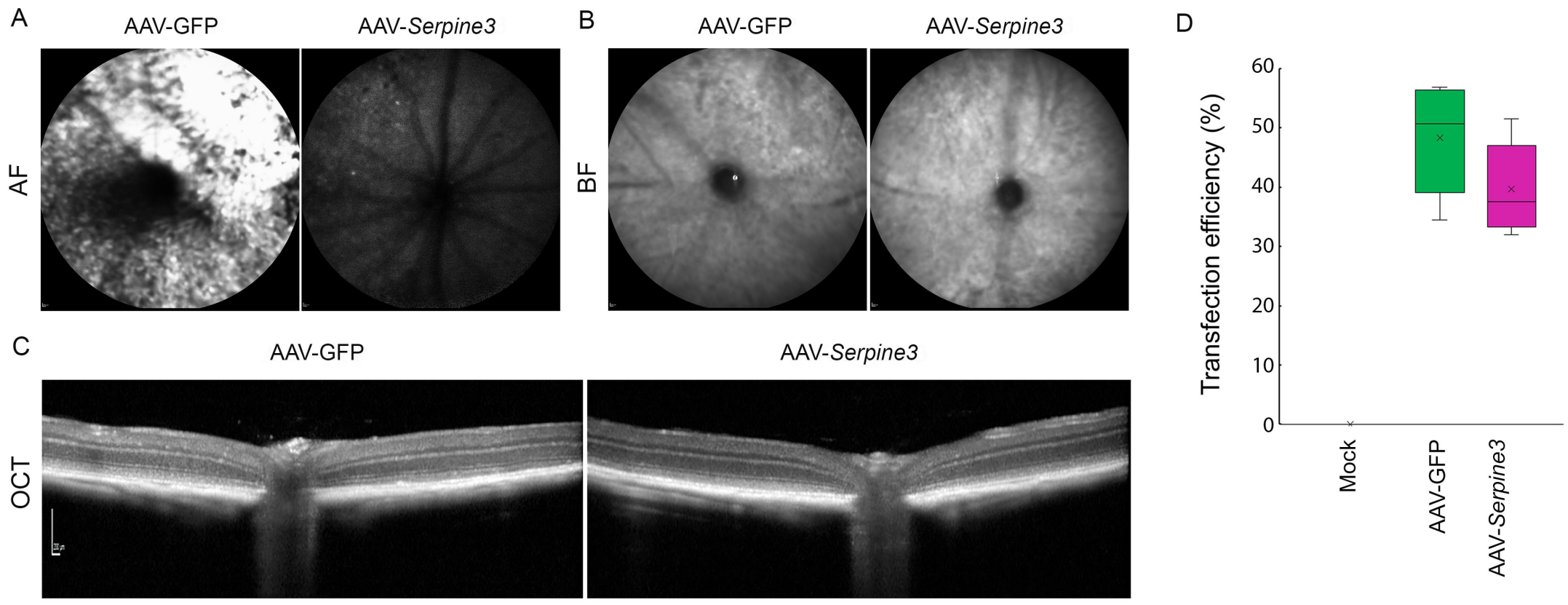

Supplemental Figure 11. AAV-Serpine3 transduction does not cause any detectable changes in 2-month-old C57BL/6J mice. (A, B) Four weeks after AAV transduction, autofluorescence (A) and bright field (B) fundus images are shown. (C) OCT images of retinal structures in mice transduced with AAV-GFP or AAV-SERPINE3 for 4 weeks. (D) Bar graphs show transduction efficiency for both vectors at 4 weeks post-injection.

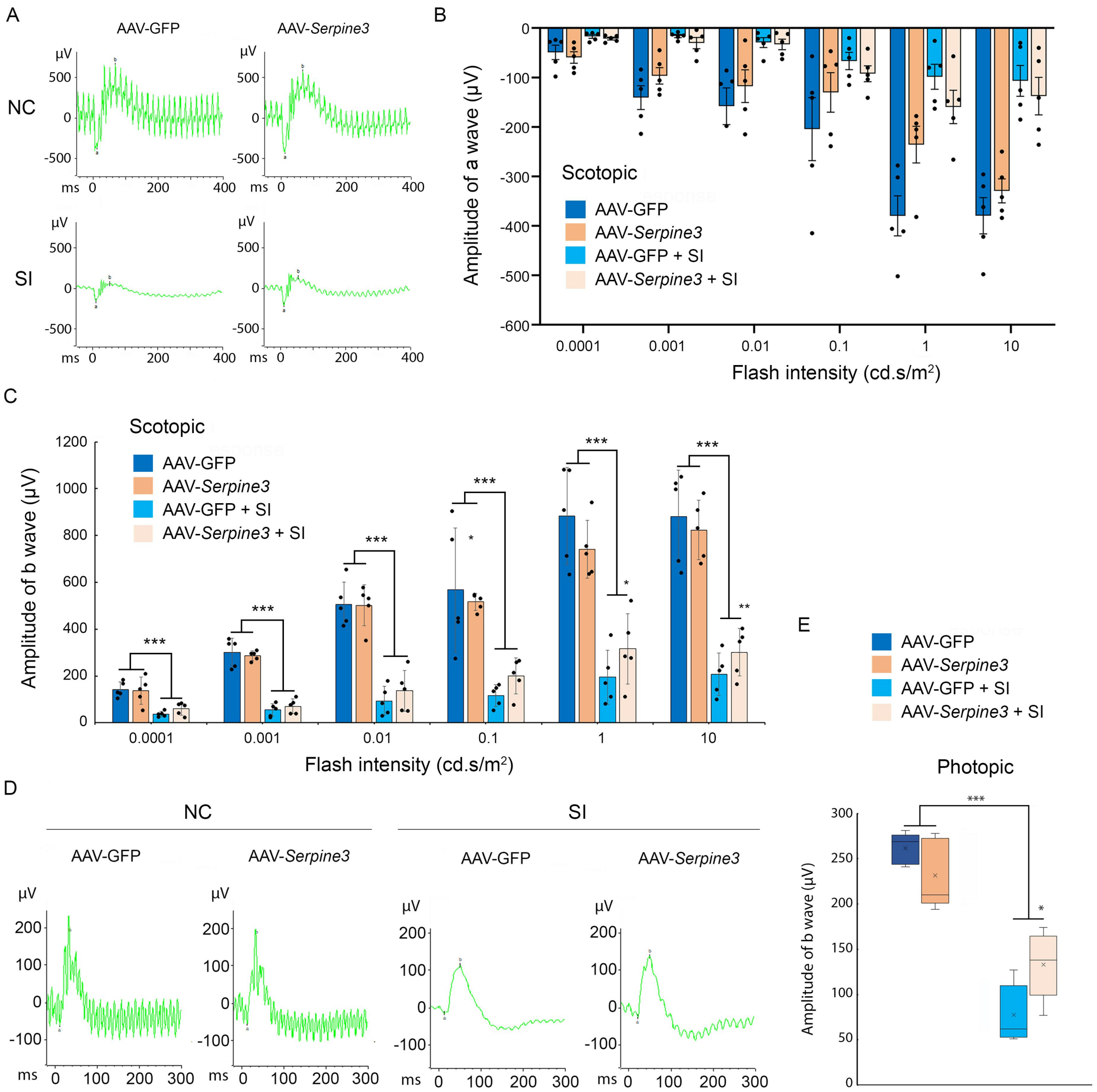

Supplemental Figure 12. AAV-SERPINE3 confers marginal preservation of retinal function in the SI injury model. (A) Scotopic ERG traces at 10 cd·s/m² from wildtype mice transduced with AAV-GFP or AAV-Serpine3, with or without SI treatment. (B, C) Quantification of a-wave (B) or b-wave (C) amplitude at flash intensities from 0.0001 to 10 cd·s/m². (D, E) Photopic ERG traces (D) and b-wave amplitude quantification (E) at 30 cd·s/m² (n=5). Data presented as mean ± SD. Statistics were calculated by paired 2-tailed Student's t test (B, C and E). *<0.05, **<0.01, ***<0.001.

Key resource table

REAGENT or RESOURCE	SOURCE	IDENTIFIER
**Antibodies**		
Rabbit anti-SERPINE3	Abcam	ab188155 (IF, 1:50; WB, 1:500)
Mouse anti-PMEL	Invitrogen	MA5-16712 (IF, 1:100)
Mouse anti-RPE65	Abcam	Ab231782 (IF, 1:100)
Rabbit anti-ZO1	Invitrogen	WK338145 (IF, 1:100)
Mouse anti-EZRIN	Abcam	Ab4069 (WB, 1:1000)
Rabbit anti-MITF	Sigma	HPA003259 (IF, 1:100)
Mouse anti-MITF	Abcam	Ab12039 (IF, 1:1000)
Rabbit anti-GFP conjugated with Alexa Fluor™ 488	Invitrogen	A-21311 (IF, 1:100)
Rabbit anti-GFP	Abcam	AB290 (WB, 1:1000)
Mouse anti-GFP	Proteintech	66002-1-Ig (1:100)
Mouse anti-4HNE	Proteintech	68538-1-Ig (1:100)
Goat anti-OTX2	R&D	AF1979 (IF, 1:200)
Rabbit anti-IBA1	Wako	019-19741 (IF, 1:300; WB, 1:1000)
Rabbit anti-Caspase1	Cell Signal Technology	24232 (WB, 1:1000)
Rabbit anti-Cleaved-caspase1	Cell Signal Technology	89332 (WB, 1:1000)
Rabbit anti-Myc	Abcam	ab9106 (co-IP, 1:10)
Rabbit anti-Flag	Cell Signal Technology	14793 (IF, 1:100; WB, 1:1000)
Mouse anti-Flag	Sigma	F1804 (IF, 1:100; WB, 1:1000)
Mouse anti-PKCa	Santa Cruz	Sc-8393 (IF, 1:100)
Rabbit anti-Recoverin	Invitrogen	PA5-110287 (IF, 1:300; WB, 1:2000)
Alexa Fluor 594 Phalloidin	Thermofish	A12381 (1:300)
Rabbit anti-HA	Abcam	Ab9110 (co-IP, 1:10)
Rabbit anti-TOMM20	Proteintech	11802-1-AP (1:200)
Rabbit anti-Vimentin	Abcam	AB92547 (1:200)
Mouse anti-Rhodopsin	Abcam	Ab98887 (1:300)
Rabbit anti-SERPINE3	Thermofish	PA5-62514 (1:30)
**Plasmids and virus strain**		
Caspase 1 (CASP1) (NM_033292) Human Tagged ORF Clone	Origene	RC218364
SERPINE3 (NM_001101320) Human Tagged ORF Clone	Origene	RC225688
pCMV6-Entry, mammalian vector with C-terminal Myc-DDK Tag	Origene	PS100001
AAV5-BEST1-SERPINE3	NEI virus core	N/A
AAV5-BEST1-GFP	NEI virus core	N/A
Lenti-Scramble-GFP	Merck	SHC216V
Lenti-SERPINE3-GFP	Merck	TRCN0000263006
**Chemicals, peptides, and recombinant proteins**		
Mouse FGF-basic (FGF-2/bFGF) Recombinant Protein	Gbico	450-33-1MG
Recombinant Caspase 1 human	Sigma	C5482-100UN

Recombinant Human Serpin E3(SERPINE3)	Cedarlane	116484
Bovine serum albumin	Sigma-Aldrich	A7906
Paraformaldehyde	Sigma-Aldrich	158127
40,6-diamidino-2-phenylindole (DAPI)	Invitrogen	D1306
Kanamycin Sulfate	ThermoFisher	15160054
DH5α Competent Cells	ThermoFisher	EC0112
LB medium	KD medical	BLE-3030
Phosphate buffered saline	Sigma	P3812
Pen-strep	life tech	15070063
Hyaluronidase	Sigma	HX0514-1
Bolt™ Bis-Tris Plus Mini Protein Gels, 4-12%, 1.0 mm, WedgeWell™ format	Thermofisher	NW04120BOX
**Critical commercial assays**		
Unique Dual Index Kit (1–24)	Takara	634756
SMART-Seq® mRNA LP	Takara	634768
NucleoMag® NGS Clean-up and Size Select	Takara	744970.50
Agilent High Sensitivity DNA Kit	Agilent	5067-4626
Caspase-Glo® 1 Inflammasome Assay (10ml)	Promega	G9951
Click-iT™ Plus TUNEL Assay Kits for In Situ Apoptosis Detection	ThermoFisher	C10618
Pierce™ Co-Immunoprecipitation Kit	Thermo Scientific	26149
EdU (5-ethynyl-2'-deoxyuridine)	Thermofish Scientific	A10044
Click-iT™ EdU Cell Proliferation Kit for Imaging, Alexa Fluor™ 594 dye	Thermofish Scientific	C10339
RNAscope-Serpine3 probe	ACD	1287098-C1
TrueBlack	Biotium	23007
Proteinase assay	Reaction biology Corp	N/A
Mutagenesis	LifeSct	N/A
Genotyping	Transnetyx	N/A
**Experimental models: Cell lines**		
Human embryonic kidney 293 cells (HEK293)	ATCC	CRL-1573
**Experimental models: Organisms/strains**		
Serpine3-Gfp KI mouse	Genetic Engineering Core, NEI	N/A
**Oligonucleotides**		
Serpine-LF1	Transnetyx	CACATCCCTGGTAGTCATGGCAGG
Serpine-LR1	Transnetyx	GAAGATGGTGCGCTCCTGGAC
Serpine-RF1	Transnetyx	CGGCCCTAGAGCTCGCTGAT
Serpine-RR1	Transnetyx	CCTGACCAGCTTGGATGTATTCTC
Gfp-F1	Transnetyx	GTACTTCCCCTCCCTCTGAACT
Gfp-R1	Transnetyx	ACCACCCCGGTGAACAG
WT-F2:	Transnetyx	GAACTGCAGGTGTCCAGTCTCC
WT-R2:	Transnetyx	AGCCACAGGCCTTCACTGA

Serpine3-F1	Life Science	AAGGCCAAGATGGAGCTTTCA
Serpine3-R1	Life Science	TACTGAAGACAAACCCTGTGCT
Actin-F1	Life Science	CACTGTCGAGTCGCGTCC
Actin-R1	Life Science	TCATCCATGGCGAACTGGTG
