# Supplementary material for: A peripheral subpopulation of retinal pigment epithelium resists oxidative damage through SERPINE3-mediated caspase-1 inhibition
Source: J Clin Invest. 2026 Jul 7;136(17):e191272. doi: 10.1172/JCI191272 (PMC13528924; doi:10.1172/JCI191272)
Supplement: Supplementary file 2 [file jci-136-191272-s137.pdf]

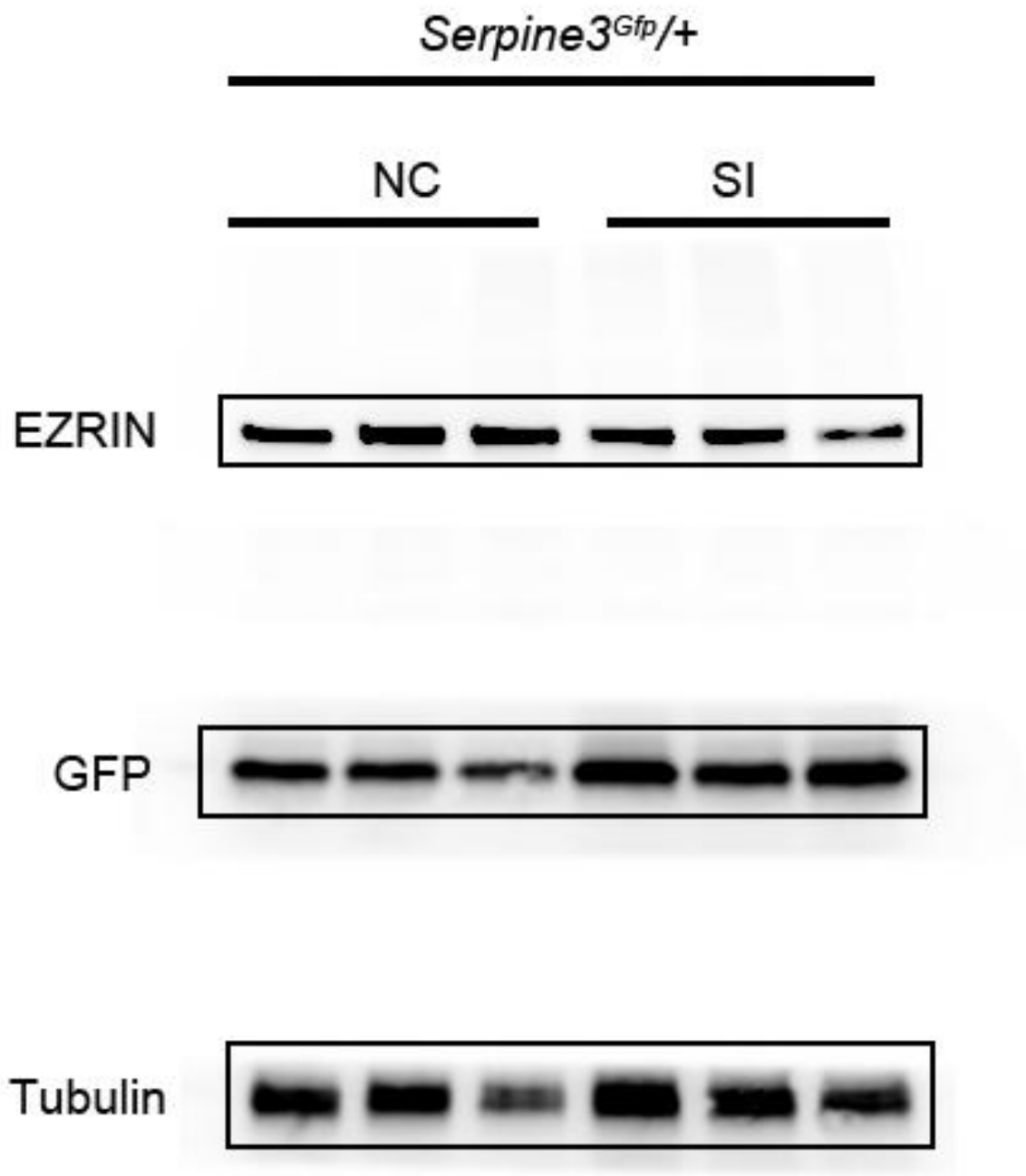

Figure 3B

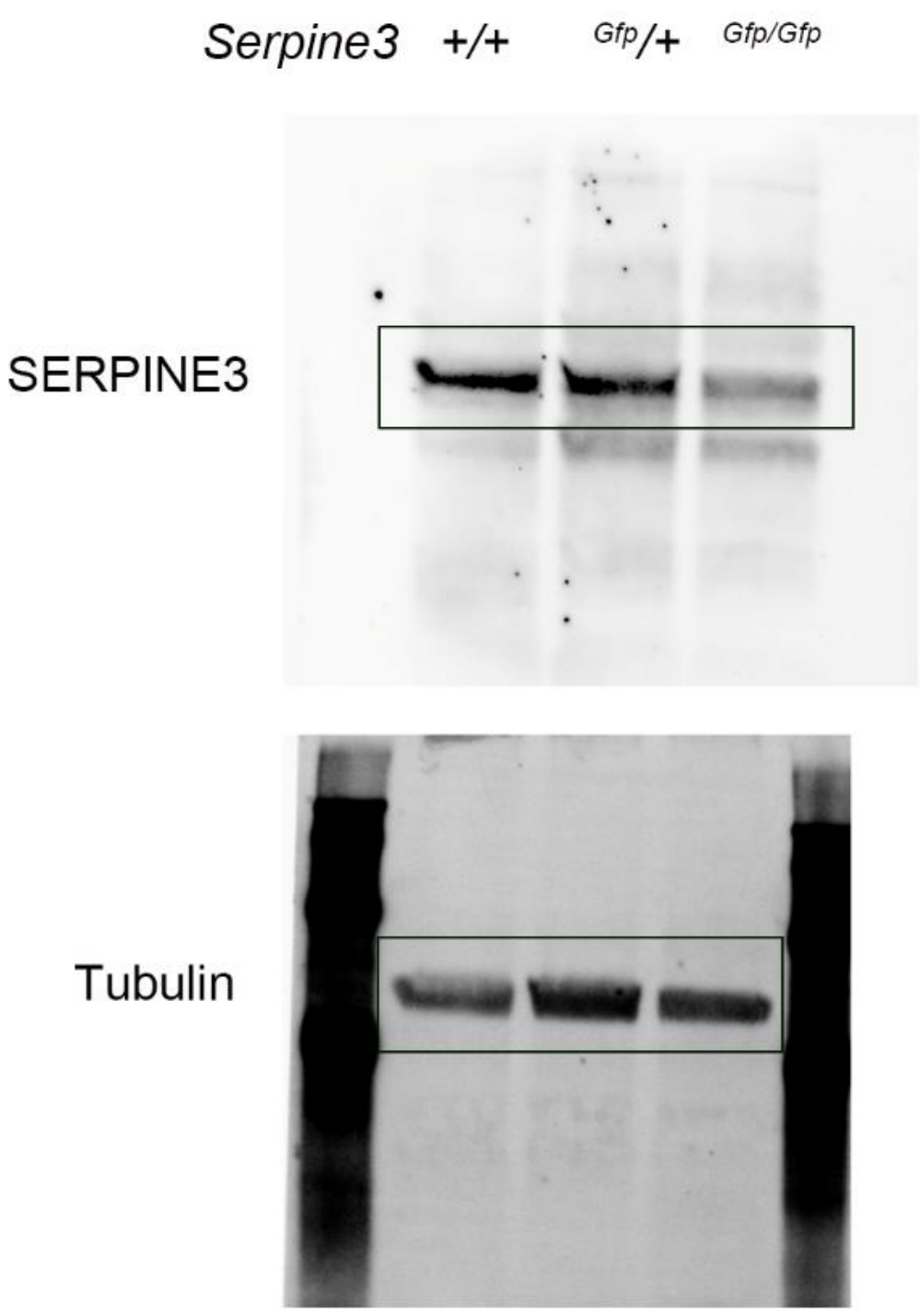

Figure 4C

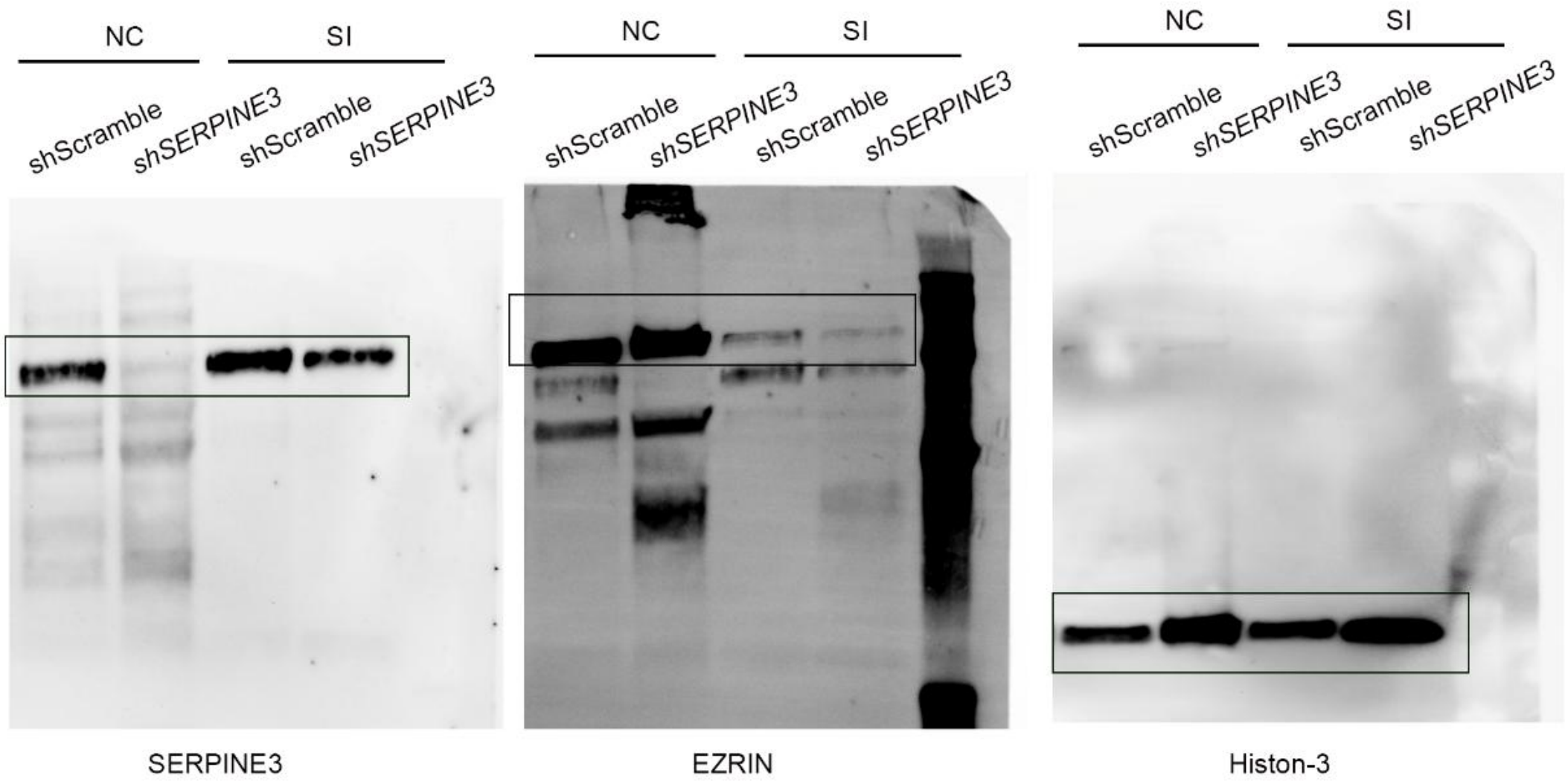
NC
SI
shScramble
shSERPINE3
shScramble
shSERPINE3
SERPINE3
EZRIN
Histon-3

Figure 4K

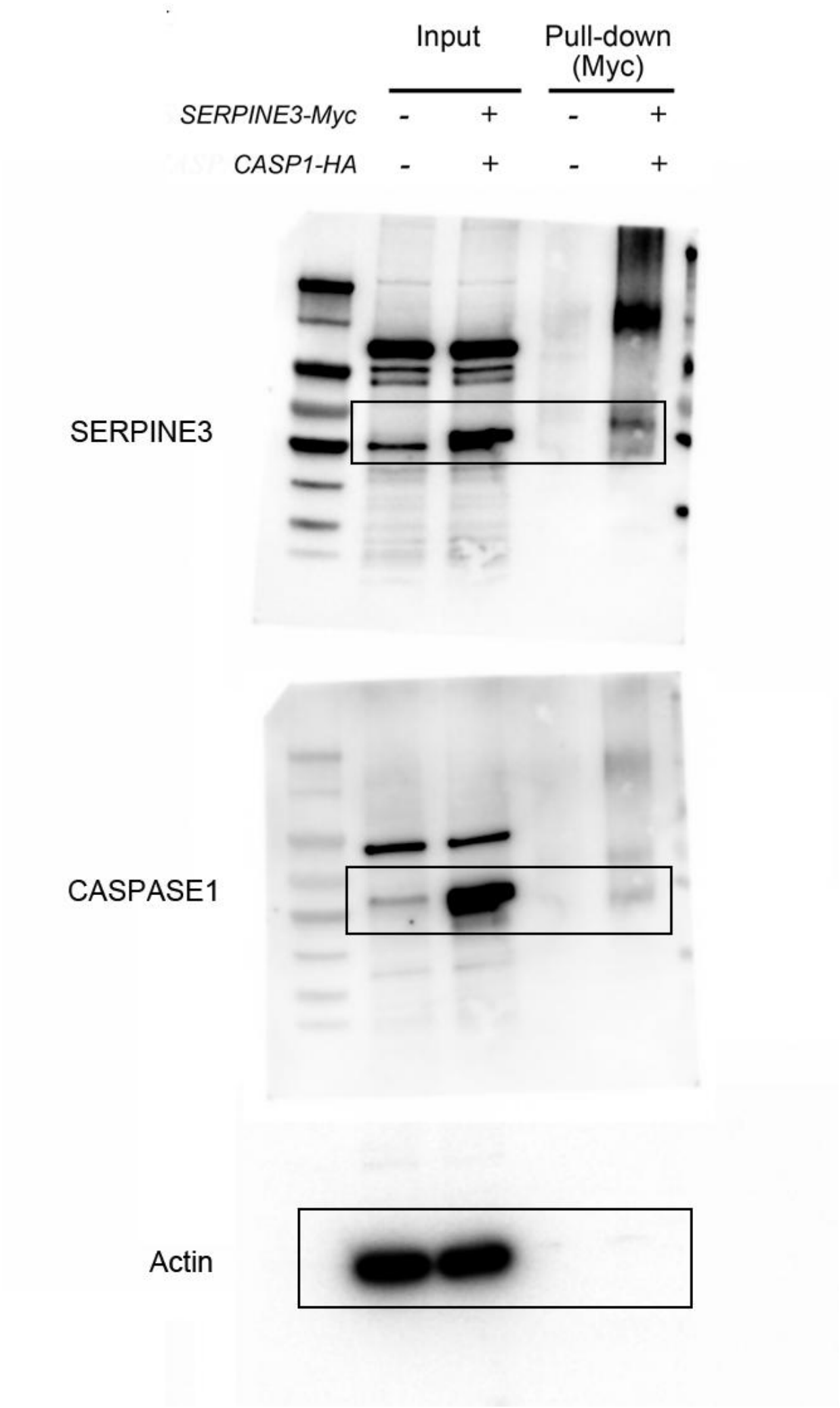
Input
Pull-down (Myc)
SERPINE3-Myc - + - +
CASP1-HA - + - +
SERPINE3
CASPASE1
Actin

Figure 6F

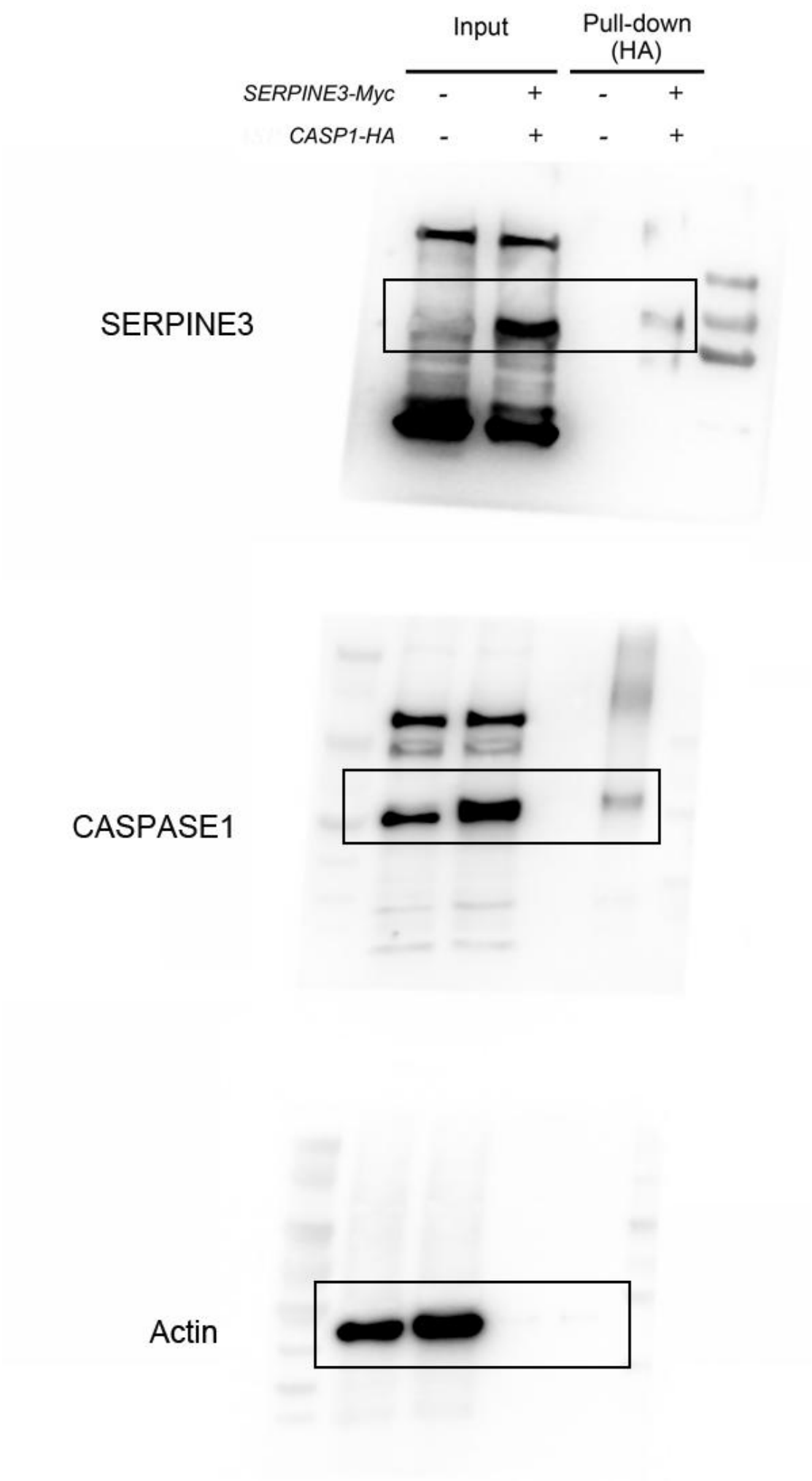
Input
Pull-down (HA)
SERPINE3-Myc - + - +
CASP1-HA - + - +
SERPINE3
CASPASE1
Actin

Figure 6G

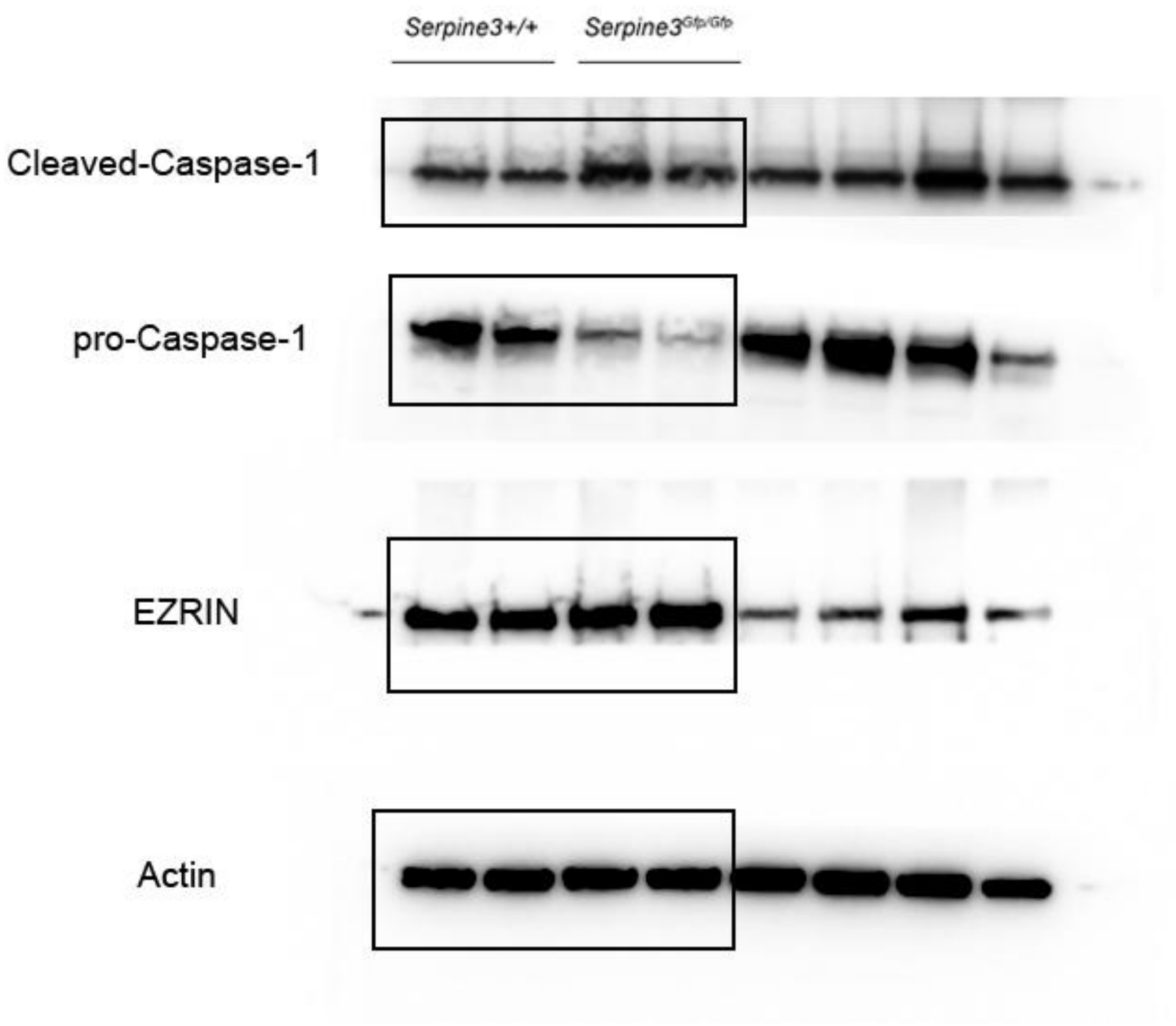
Serpine3+/+
Serpine3Gfp/Gfp
Cleaved-Caspase-1
pro-Caspase-1
EZRIN
Actin

Figure 6H

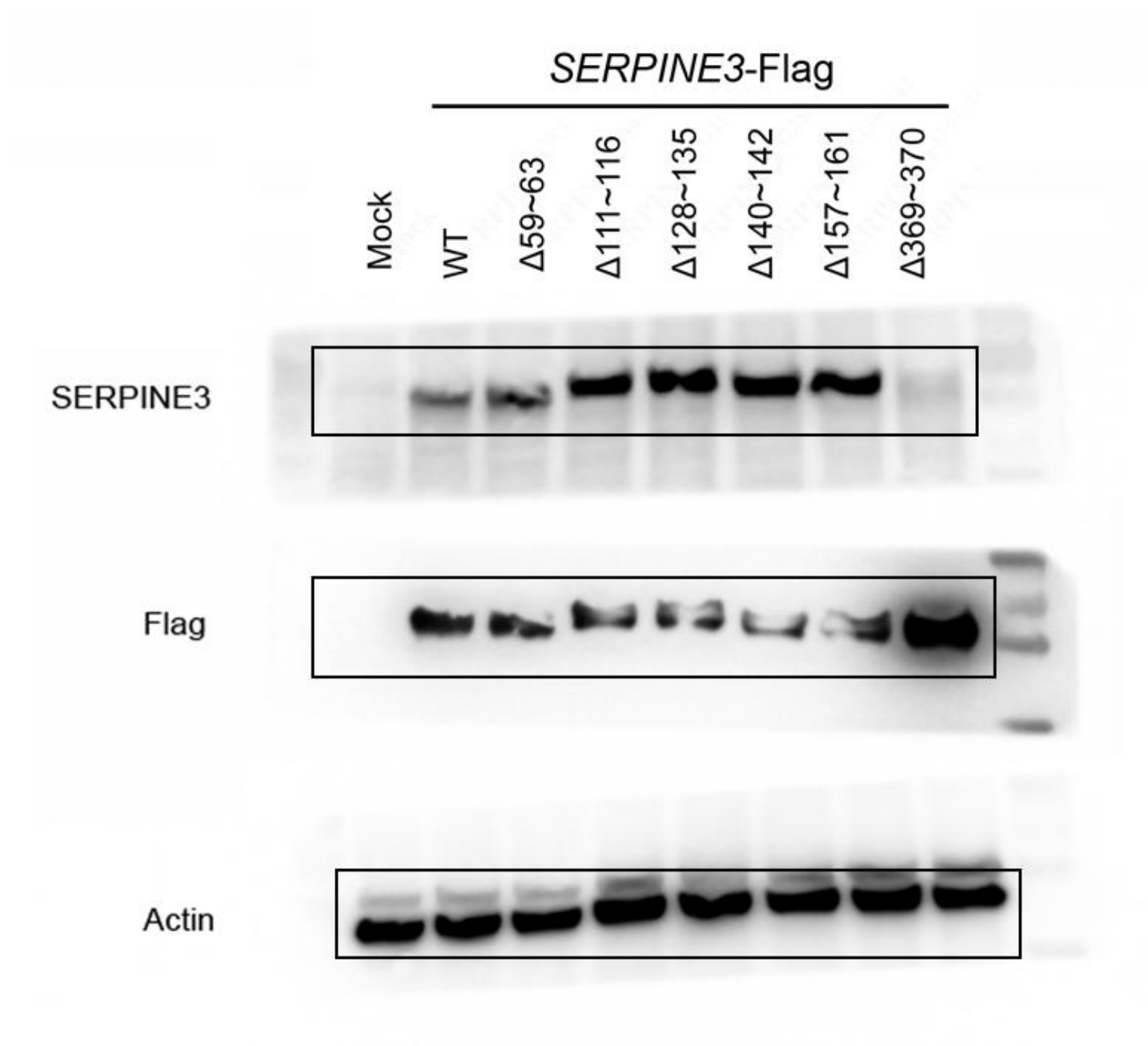
SERPINE3-Flag
Mock
WT
Δ59~63
Δ111~116
Δ128~135
Δ140~142
Δ157~161
Δ369~370
SERPINE3
Flag
Actin

Figure 7B

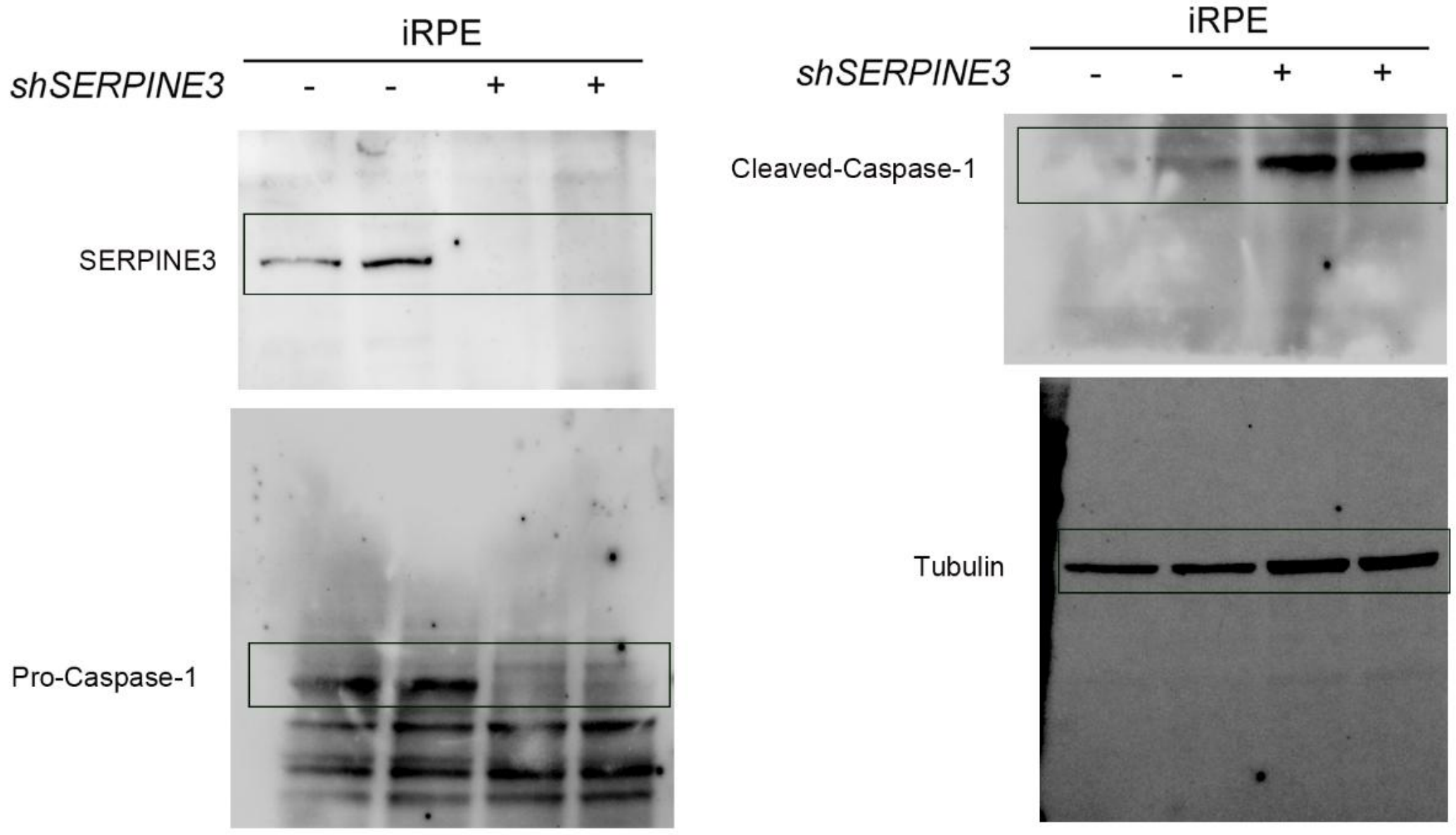
iRPE
shSERPINE3 - - + +
SERPINE3
Pro-Caspase-1
iRPE
shSERPINE3 - - + +
Cleaved-Caspase-1
Tubulin

Figure 8A

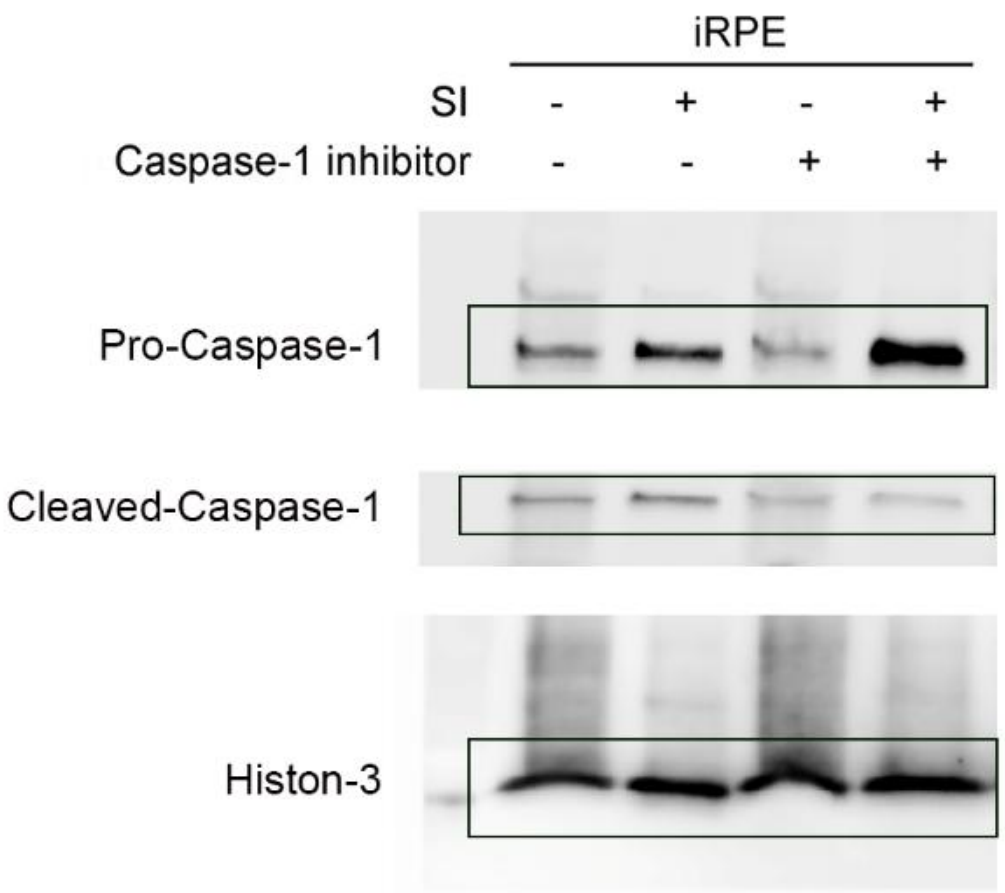
iRPE
SI - + - +
Caspase-1 inhibitor - - + +
Pro-Caspase-1
Cleaved-Caspase-1
Histon-3

Figure 8C

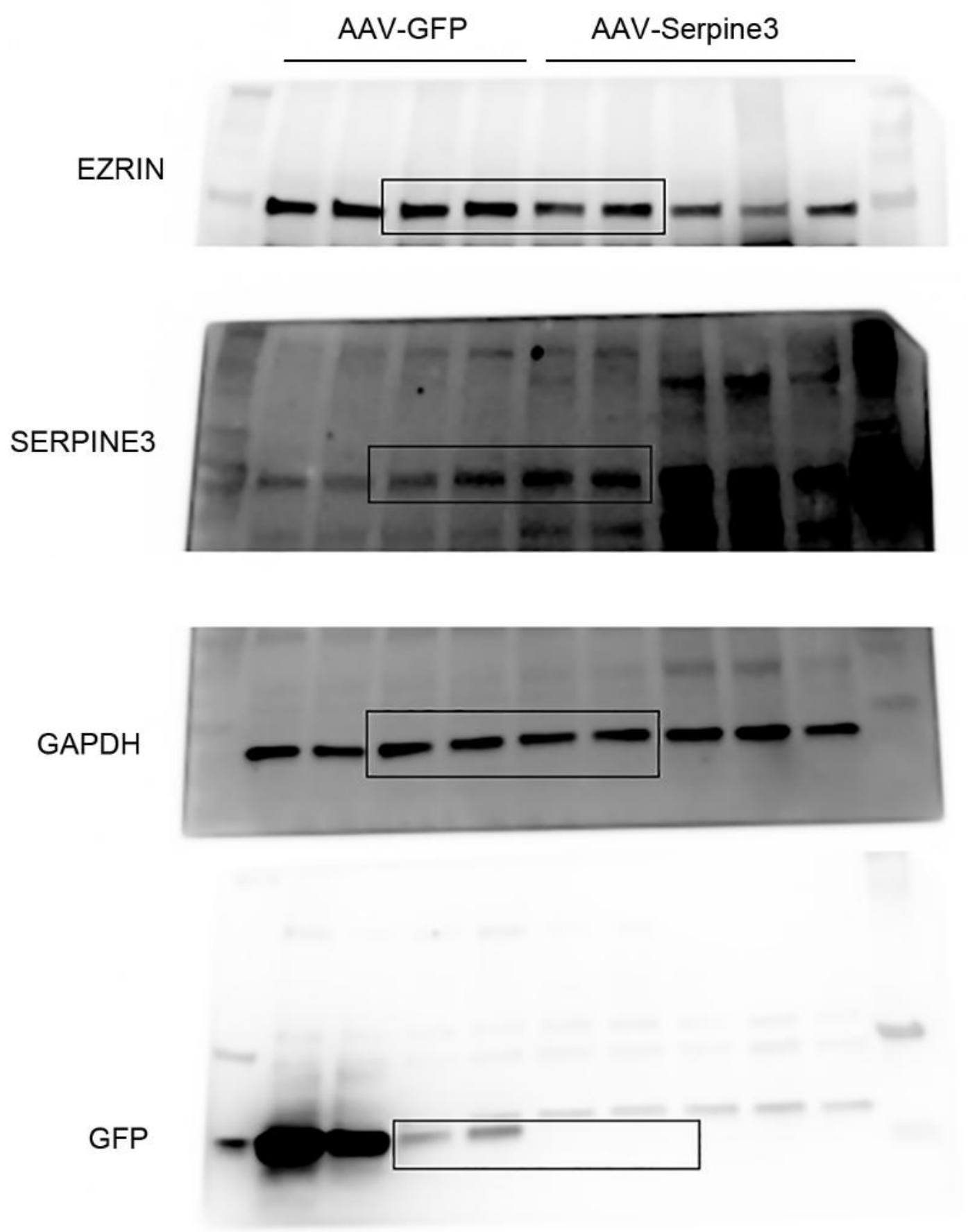

Figure 9B

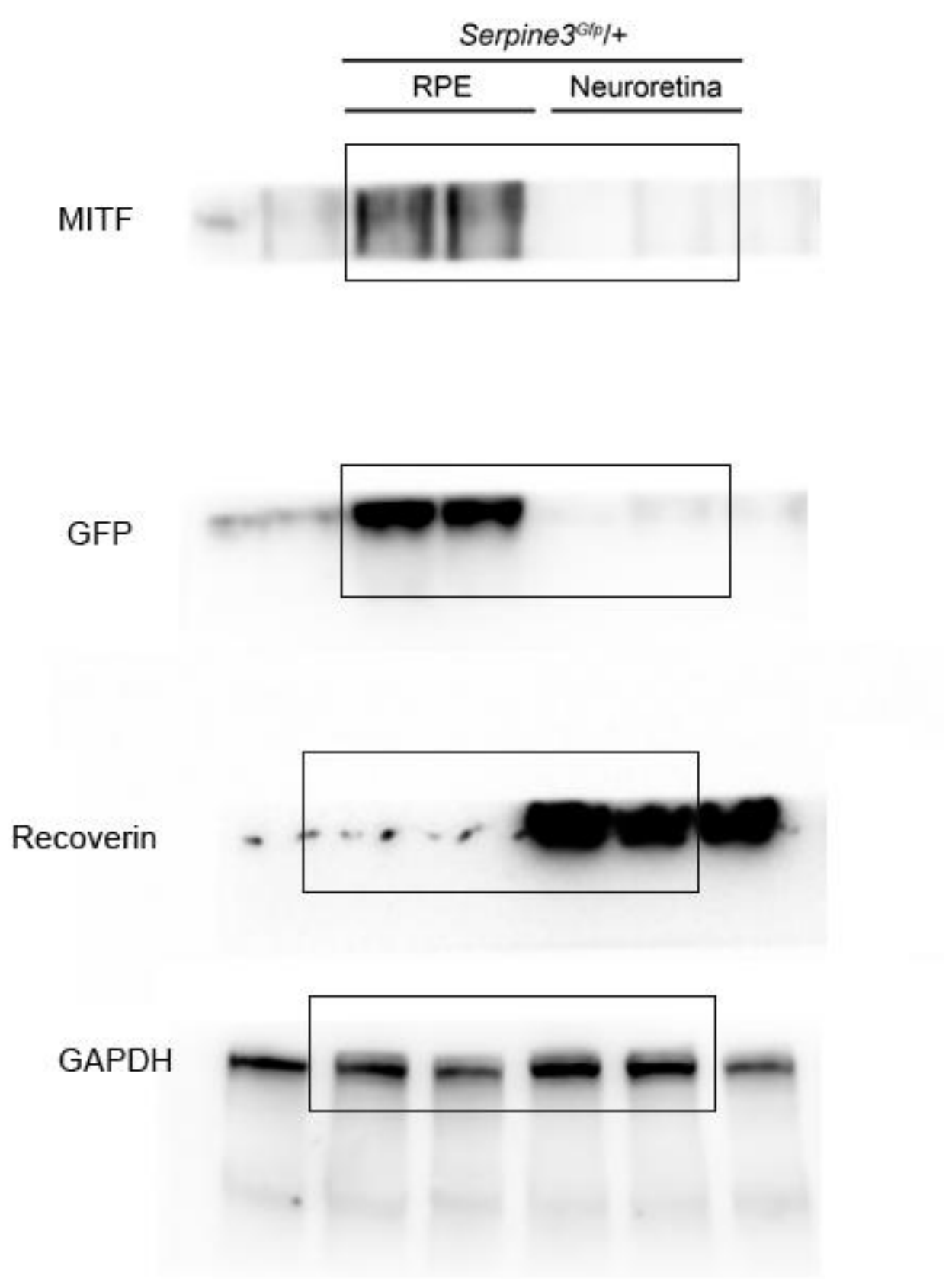

Supplemental Figure 1B

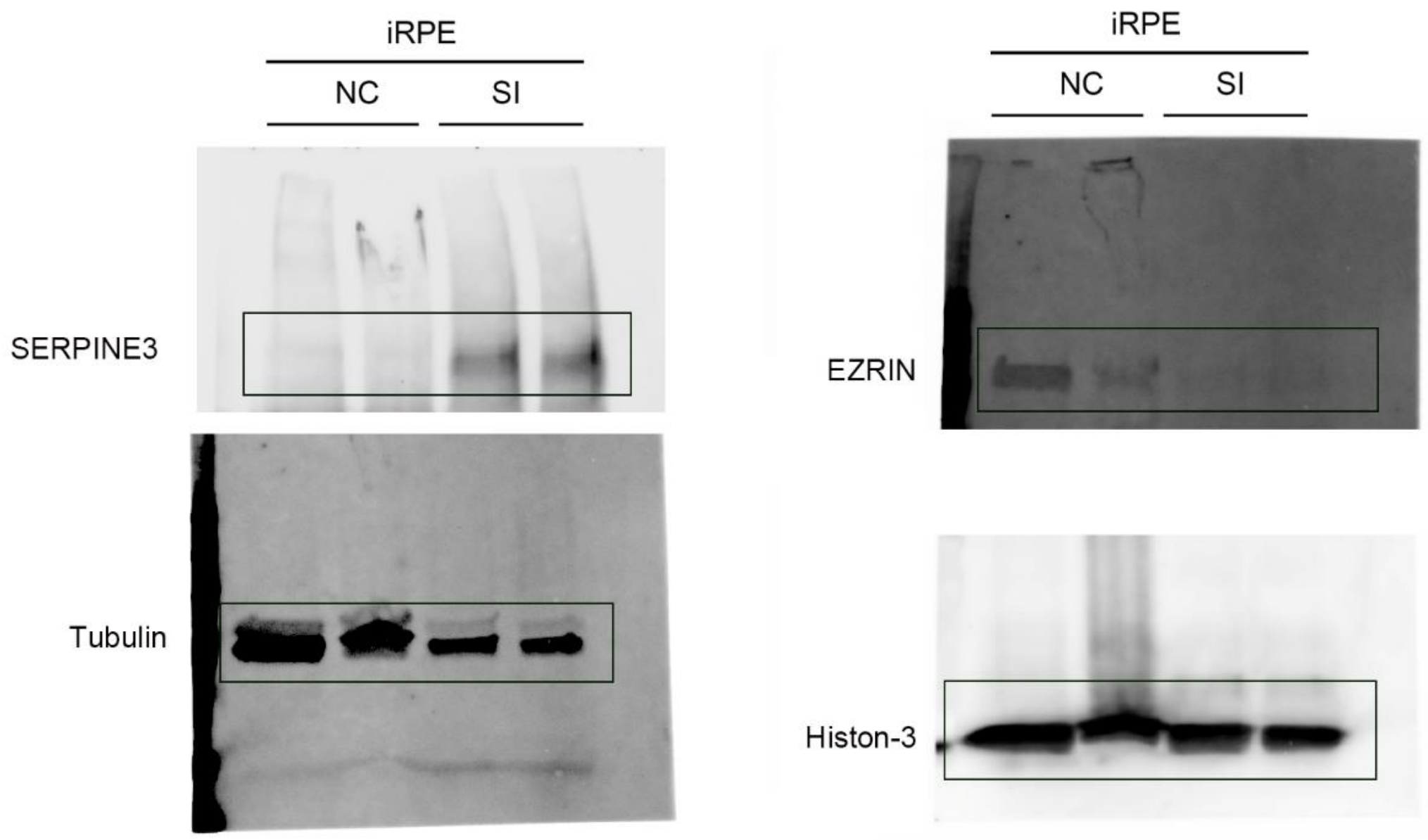

Supplemental Figure 3A

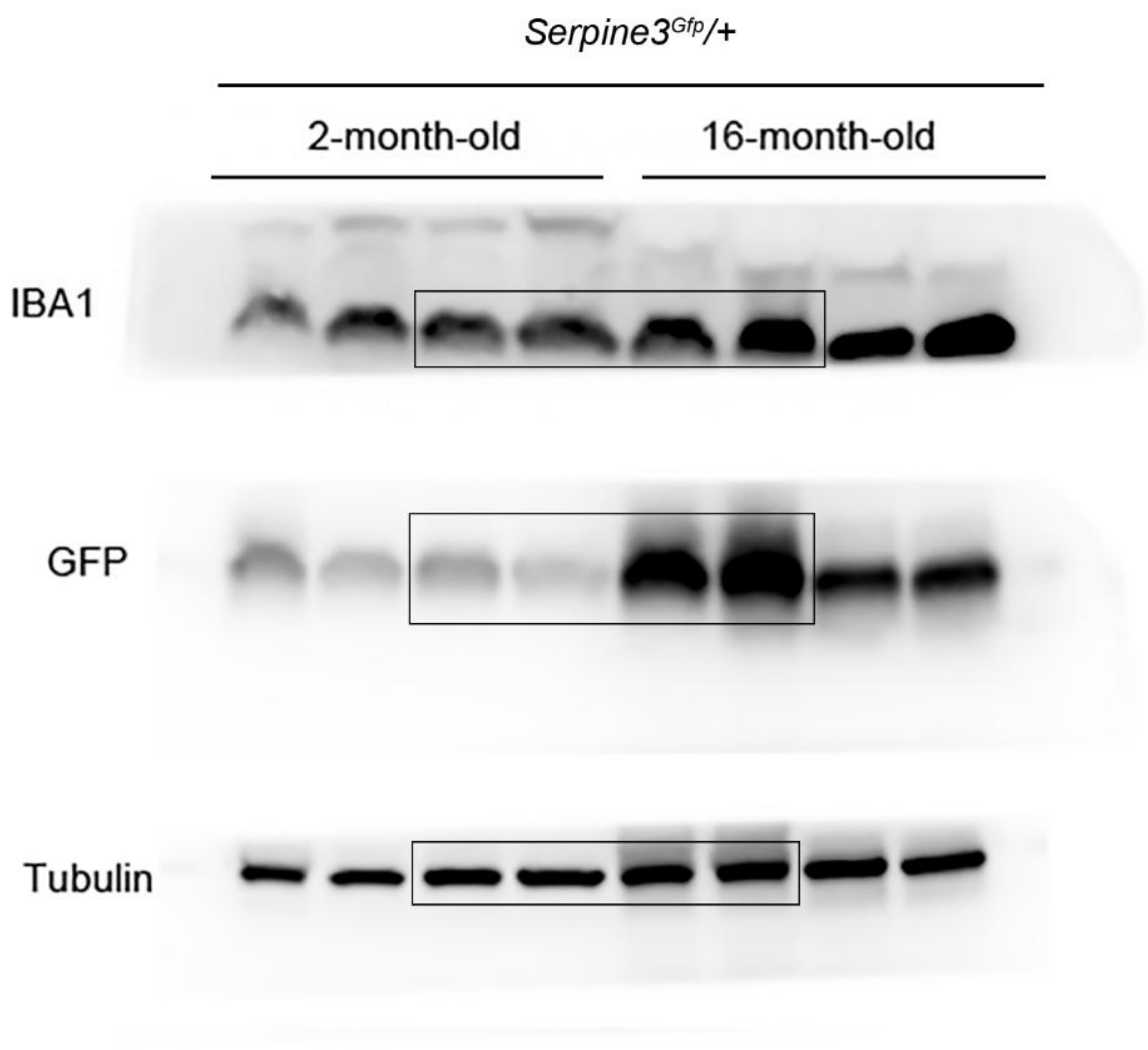

Supplemental Figure 6

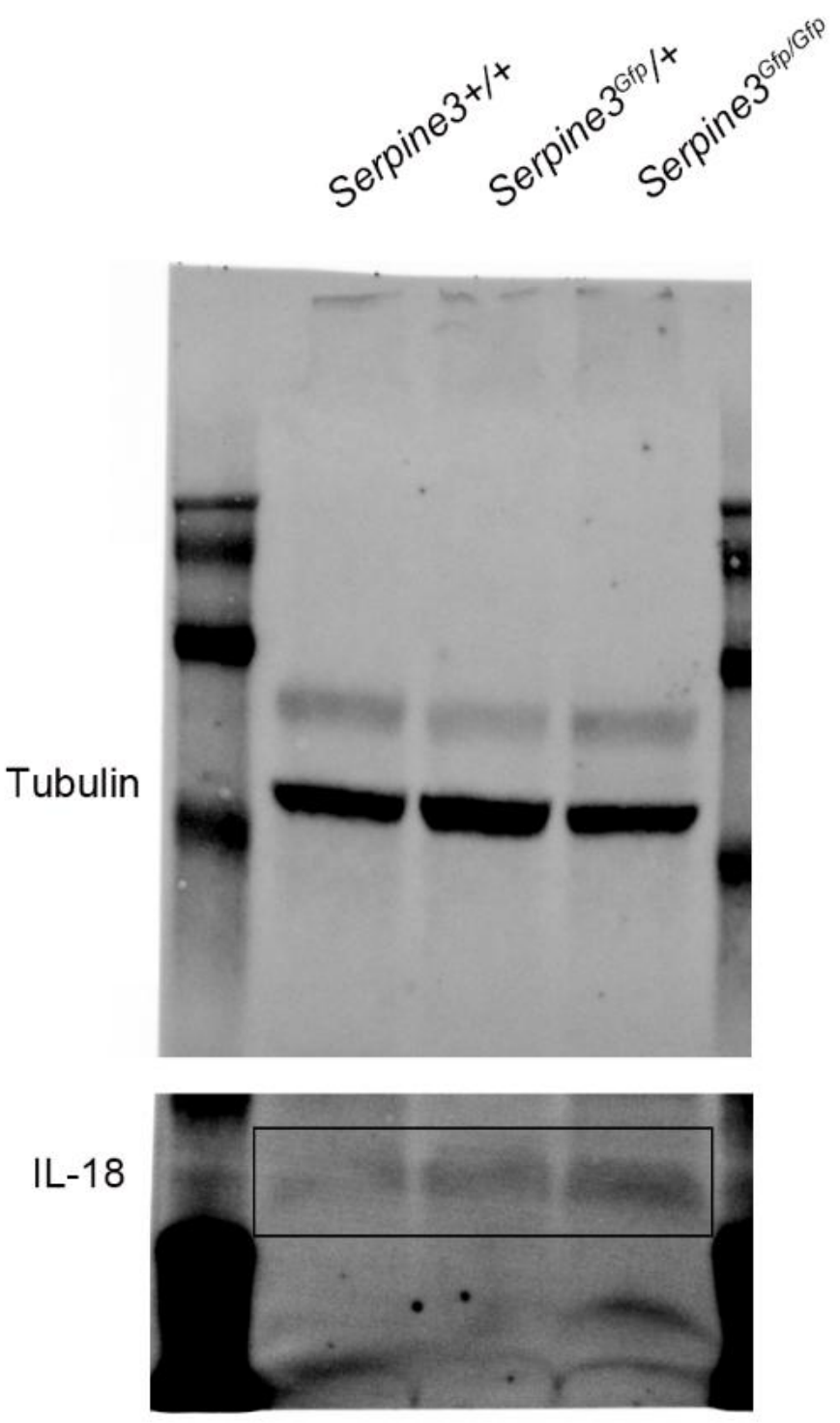

Supplemental Figure 8C
